# FeCl_3_‐Catalyzed Synthesis of Ynones from Silylated Alkynes and Acetic Anhydride

**DOI:** 10.1002/open.202500402

**Published:** 2025-10-27

**Authors:** Paul Charki, Urs Gellrich, Daniel S. Müller

**Affiliations:** ^1^ CNRS Institut des Sciences Chimiques de Rennes‐UMR6226 Univ Rennes F‐35000 Rennes France; ^2^ Department of Organic Chemistry University of Hohenheim Garbenstrasse 30 70599 Stuttgart Germany

**Keywords:** alkynes, anhydrides, enynones acid, iron catalysis, lewis, silicon

## Abstract

The reaction of silylated alkynes with acid chlorides in the presence of Lewis acids, first described by Birkhofer in 1963, has since emerged as a valuable method for the synthesis of alkynones. Despite its broad synthetic utility, the original protocol suffers from notable drawbacks, including the use of toxic solvents, stoichiometric Lewis acids, and corrosive acylating agents. In light of growing environmental concerns, a more sustainable alternative is developed. Herein, catalytic amounts of iron(III) chloride in combination with biodegradable acetic anhydride enable the efficient synthesis of alkynones under mild conditions.

## Graphical abstract

The synthesis of alkynones from silylated alkynes and acid chlorides, first reported by Birkhofer in 1963, remains a valuable transformation. However, its reliance on stoichiometric AlCl_3_ and corrosive reagents raises environmental and practical concerns. To address this, a greener protocol using catalytic FeCl_3_ and acetic anhydride is developed. The method avoids toxic solvents and harsh reagents, delivering alkynones efficiently under mild conditions.

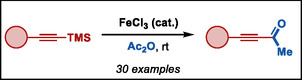

## 1 Introduction

**Scheme 1:**
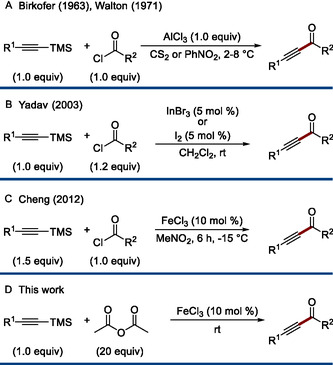
Previous work and this work.

Alkynones are versatile synthetic intermediates in organic chemistry,^[^ ^1^ ^–^ ^4^ ^]^ particularly valued for their ability to access structurally diverse heterocycles.^[^ ^5^ ^]^ Accordingly, numerous methods for their preparation have been developed,^[^ ^2^, ^3^ ^,^ ^6^ ^]^ including nucleophilic addition of metal acetylides to carboxylic acid derivatives (Previous strategies were summarized in ref. [7]),^[^ ^8^ ^]^ palladium‐catalyzed coupling reactions,^[^ ^9^ ^]^ and carbonylative protocols.^[^ ^10^ ^]^ A conceptually distinct strategy was introduced by Birkofer and co‐workers, who demonstrated that silylated alkynes undergo Friedel–Crafts‐type acylation with acid chlorides in the presence of stoichiometric amounts of AlCl_3_ (**Scheme** **1**A).^[^ ^11^ ^]^ This approach was subsequently optimized by Walton and co‐workers,^[^ ^12^ ^]^ who found that the use of dichloromethane as solvent significantly improved the yields of this transformation for certain products. The Birkofer–Walton protocol has since seen broad application in synthesis, including total synthesis of natural products.^[^ ^13^, ^14^, ^15^ ^–^ ^16^ ^]^

However, its reliance on stoichiometric aluminum trichloride and corrosive acid chlorides raises concerns regarding environmental impact and operational safety, underscoring the need for a more modern and environmentally responsible approach.

In response to these drawbacks, Yadav introduced a modified variant employing catalytic amounts of InBr_3_ or iodine with dichloromethane as a solvent (Scheme 1B).^[^ ^17^ ^,^ ^18^ ^]^ More recently, Cheng and co‐workers demonstrated that inexpensive FeCl_3_ serves as a viable and environmentally benign catalyst (Scheme 1C). It should be noted that FeCl_3_ is frequently described as an environmentally benign catalyst, see ref. [19]. However, the compound is inherently corrosive and its relative benignity arises primarily from its effective catalytic activity, which enables its use in substoichiometric amounts. In this respect, FeCl_3_ presents a more favorable environmentally profile compared to stoichiometric AlCl_3_. ^[^ ^20^, ^21^ ^–^ ^24^ ^]^ However, the use of nitromethane as solvent—due to its propensity for highly exothermic decomposition^[^ ^25^ ^]^—raises significant safety concerns, particularly in the context of scale‐up. Motivated by these advances and by Birkhofer's early observations on the use of acetic anhydride as a more benign acyl source,^[^ ^11^ ^]^ we decided to reinvestigate this reaction. It is of interest that over the past decades, numerous alternatives to acyl chlorides have been investigated for the synthesis of enyones, including esters,^[^ ^26^ ^,^ ^27^ ^]^ acid anhydrides,^[^ ^28^ ^]^ in situ activated acids,^[^ ^29^ ^,^ ^30^ ^]^ and aldehydes.^[^ ^31^ ^]^

Herein, we describe an operationally simple and efficient FeCl_3_‐catalyzed acetylation with acetic acid anhydride^[^ ^32^, ^33^, ^34^, ^35^ ^]^ and silylalkynes^[^ ^36^ ^]^ that proceeds under mild conditions with good yields (Scheme 1D). (For the synthesis of silyl alkynes from lithium acetylides, see ref. [37]. For our recent work with alkynyl silanes, see ref. [38]. For a review on alkynyl silanes: See ref. [39]. For a recent review on the synthesis of alkynyl silanes: See ref. [40]).

## 2 Results and Discussion

We initiated our investigations with scandium triflate, a Lewis acid previously shown to catalyze the acylation of anisole with acetic anhydride **2** under mild conditions.^[^ ^41^, ^42^, ^43^ ^]^ After some experimentation, we found that 10 mol% of Sc(OTf)_3_ efficiently promoted the acylation of **1** at room temperature using an excess of acetic anhydride under neat conditions (entry 1, **Table** **1**). According to Cheng's report,^[^ ^22^, ^23^ ^]^ we next examined inexpensive FeCl_3_ as Lewis acid and were pleased to observe improved yields relative to Sc(OTf)_3_ (entry 4, Table 1). The use of freshly distilled Ac_2_O showed no improvement (cf. entries 4 and 5); thus, technical grade Ac_2_O was employed throughout the rest of the study. Interestingly, InCl_3_ and BF_3_^.^OEt_2_ also afforded enynone **3** in good yields (entries 7–8). For a complete evaluation of Lewis acids and additional optimization reactions, see Table S1 in the supporting information. It is noteworthy that stirring phenyltrimethylsilylacetylene **1** with acetic anhydride **2** and acetic acid at 20 °C for 1 h resulted in no observable conversion of **1** or formation of product **4** (see Supporting Information for details).

**Table 1:** Optimization reactions for the acylation of silyl alkyne 1 with acetic acid anhydride.

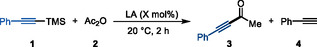					
entry	2 [equiv.]	Lewis acid [mol%]	conv. **1** a)	yield **3** a)	yield **4** a)
1	10	Sc(OTf)_3_ (10)	100	68	10
2	2	FeCl_3_ (10)	88	66	14
3	5	FeCl_3_ (10)	100	76	4
4	10	FeCl_3_ (10)	100	87	5
5b)	10	FeCl_3_ (10)	100	86	4
6	10	FeCl_3_ (5)	93	76	12
7	10	InCl_3_	95	81	10
8	10	BF_3_·OEt_2_	100	69	4

a) Determined by GC with *n*–decane as an external standard. Values reported as percentages;

b) Distilled Ac_2_O was used.

**Scheme 2:**
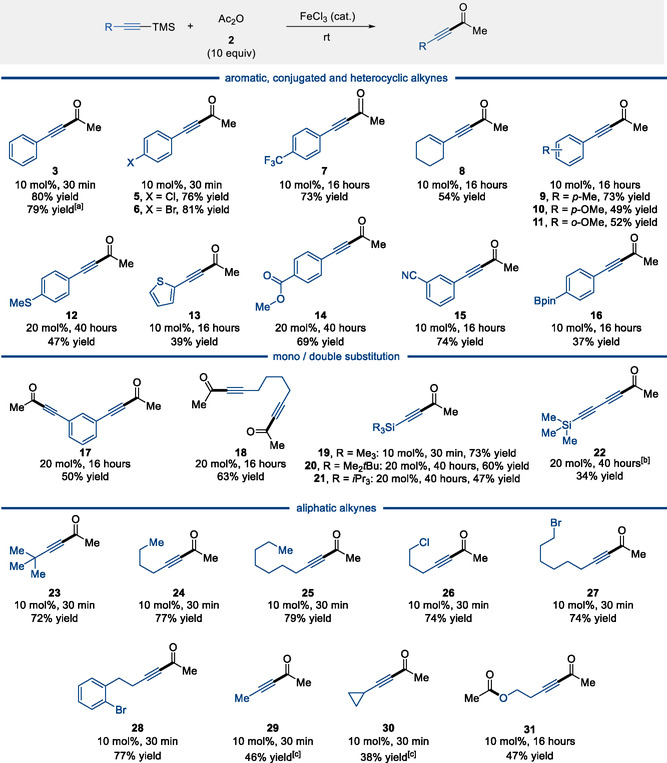
Scope of silylated alkynes. All reactions were performed on 1.0 mmol. ^[a]^Reaction performed on 15.0 mmol scale. ^[b]^Nitromethane was added to the reaction. ^[c]^Volatile compounds.

With the optimized conditions in hand (entry 4, Table 1), compound **2** was isolated in 80% yield on 0.5 mmol scale and 79% yield on 15 mmol scale (**Scheme** **2**). We next examined the scope of the transformation. Aryl‐substituted silyl alkynes bearing electron‐withdrawing groups (Cl, Br, CF_3_) were efficiently converted to the corresponding ynones (**5**–**7**). The conjugated alkyne **8** was obtained in slightly diminished yield, whereas electron‐rich alkyne **9** delivered the product in 73% yield. Anisole derivatives **10** and **11** were also tolerated, affording products in 49 and 52% yield, respectively. Substrates bearing Lewis‐basic functionality (e.g. **12**–**15**) required 20 mol% FeCl_3_ and/or prolonged reaction times for full conversion.

The pinacol borane‐substituted product **16** was obtained alongside 21% of the corresponding terminal alkyne, indicating that protodesilylation constitutes a significant side reaction in this case. Steric effects were also found to influence reactivity: while the bis‐TMS‐substituted substrate was efficiently converted to **19** in 73% yield using 10 mol% FeCl_3_, a marked decrease in yield was observed for more hindered analogs **20** and **21**. Notably, the use of nitromethane as a cosolvent proved critical for the formation of **22**; no reaction occurred in its absence. Methyl‐ and cyclopropyl‐substituted enynones (**29**, **30**) were obtained in somewhat diminished yields. Acylation of 4‐(trimethylsilyl)but‐3‐yn‐1‐ol led to concomitant esterification of the homopropargylic alcohol, affording the corresponding acetate **31**.

**Scheme 3:**
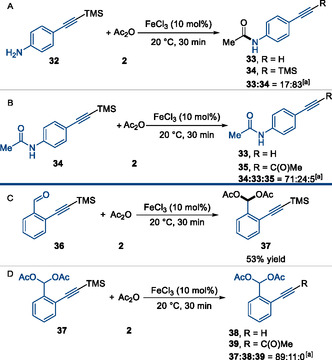
Undesired acylation of functional groups. ^[a]^Ratio estimated by GC analysis.

Substrates bearing amine **32** or benzaldehyde **36** functionalities proved incompatible with the reaction conditions, suffering from undesired side reactions such as acylation of the heteroatom^[^ ^32^, ^33^ ^–^ ^35^ ^,^ ^41^ ^]^ and partial protodesilylation (**Scheme** **3**). In both cases, no formation of the desired alkynone product was observed, even upon modification of the reaction conditions (e.g., 1.1 equiv FeCl_3_, extended reaction time). These results suggest that either the resulting intermediates (**34** and **37**) are rendered too electron poor to undergo further acylation or, alternatively, that coordination of the amide or diacetate to the Lewis acid deactivates the catalyst. We then attempted to use the protected products **34** and **37** directly in the reaction, but this resulted in only partial conversion of the starting materials, with the major products being the protodesilylated compounds **33** and **38**. Only traces of product **35** were detected at 10 mol% FeCl_3_, with no improvement observed even at 110 mol%.

**Scheme 4:**
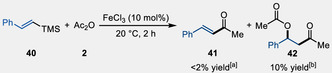
FeCl_3_‐promoted reaction of alkenyl silane **40** with Ac_2_O. ^[a]^Percentage estimated by GC analysis. ^[b]^Isolated yield.

The synthesis of enones from styrenylsilanes using acetyl chloride and AlCl_3_ as a Lewis acid has been documented in previous studies (See ref. [44]).^[^ ^45^, ^46^ ^–^ ^47^ ^]^ An investigation was conducted to determine if the established reaction conditions could be applied to yield the corresponding enone. Upon subjecting alkenyl silane **40** to the reaction conditions, only trace quantities of the desired enone **41** were observed (see gas chromatography (GC) trace in the Supporting Information). The primary product isolated was identified as 3‐oxo‐1‐phenylbutyl acetate (**42**), obtained in a modest 10% yield (**Scheme** **4**).

**Scheme 5:**
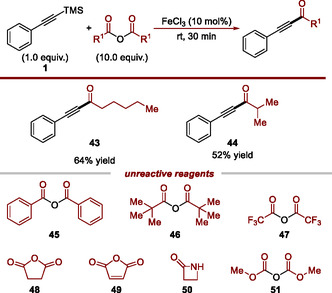
Reagent scope.

Next, we investigated the scope of anhydrides as acylating agents under our standard protocol (**Scheme** **5**). Among commercially available anhydrides tested, only hexanoic and isobutyric anhydrides successfully afforded the corresponding ynones **43** and **44** in 64 and 52% yields, respectively. Benzoic and pivalic anhydride (**45** and **46**) gave only traces of product (significant differences in reactivity between Ac_2_O and Bz_2_O were previously reported, see: ref. [48]). Similarly, trifluoroacetic anhydride, a strongly electron‐withdrawing acyl source, was unreactive. This lack of reactivity is likely due to the lack of stabilization of the corresponding acylium ion intermediate which probably does not form under the reaction conditions. We further extended our scope to cyclic anhydrides (**48** and **49**), cylic amides **50,** and carbonates **51** which are all known to undergo addition reaction with silylated alkynes in the presence of AlCl_3_.^[^ ^49^ ^,^ ^50^ ^]^ Unfortunately, none of these substrates reacted even under more forcing conditions.

### 2.1 Mechanism

Experimental elucidation of the FeCl_3_‐promoted reaction between acetic anhydride and alkynylsilanes is hindered by the paramagnetic nature of FeCl_3_, which complicates NMR analysis. The sole spectroscopic evidence was provided by Mihara,^[^ ^34^ ^]^ who reported that mixing equimolar amounts of FeCl_3_ and Ac_2_O produced a new carbonyl absorption band at 1802 cm^−1^ in the IR spectrum, consistent with a new iron complex. Consequently, we decided to further study the mechanism of the FeCl_3_‐catalyzed reaction between trimethyl(phenylethynyl)silane (1) and acetic anhydride (2) through density functional theory (DFT) computations. Structure optimizations were carried out with the three‐fold corrected r2SCAN‐3c method.^[^ ^51^ ^]^ Final single‐point energy calculations employed the *ω*B97M‐V functional in combination with the large def2‐QZVPP basis set.^[^ ^52^ ^,^ ^53^ ^]^ To approximate acetic anhydride as the solvent, the conductor‐like polarizable continuum model (CPCM) for acetone was used, which is reasonable given the similar dielectric constants of acetic anhydride and acetone.^[^ ^54^ ^]^ All computations were performed with the ORCA program package.^[^ ^55^ ^,^ ^56^ ^]^

**Figure 1:**
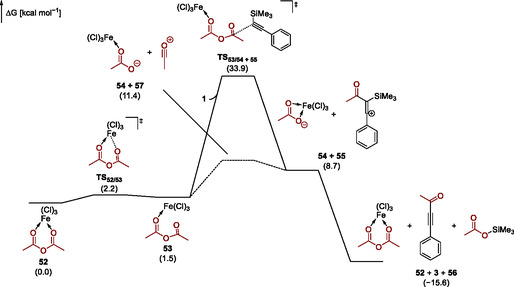
Potential energy surfaces of two mechanistic scenarios: addition of trimethyl(phenylethynyl)silane (**1**) to the Ac_2_O–FeCl_3_ complex **53** (solid line) and a dissociative pathway involving the formation of acylium ion **57** (dashed line). All Gibbs free energies were computed at the CPCM(acetone)‐wB97M‐V/def2‐QZVPP//r^2^SCAN‐3c level of theory.

One possible mechanism involves an inner‐sphere addition of **1** to acetic anhydride coordinated to FeCl_3_ (**Figure** **1**). According to our computations, this pathway commences with the cleavage of one oxygen–iron bond in the Ac_2_O–FeCl_3_ complex **52**, resulting in complex **53**, in which acetic anhydride is bound as a monodentate ligand. This intramolecular rearrangement requires overcoming a modest barrier of 2.2 kcal· mol^−1^. The subsequent addition of **1** to complex **53** forms the ion pair consisting of **55** and FeCl_3_–OAc **54** by nucleophilic displacement of the latter, imposing an overall barrier of 33.2 kcal· mol^−1^ on this pathway. The transfer of the silyl group between **54** and **55** is computed to be exergonic. We also included regeneration of the Ac_2_O–FeCl_3_ complex **52** in the computation of this step, since free FeCl_3_ is unlikely under the reaction conditions.

Because an overall barrier exceeding 30 kcal· mol^−1^ is too high for a reaction taking place at room temperature, we also considered a dissociative mechanism involving the liberation of acylium ion **57** from complex **53**. The computed Gibbs free energy for this dissociation is 11.4 kcal· mol^−1^. However, a detailed potential energy surface scan indicates that the acylium ion remains coordinated to the FeCl_3_–OAc complex even after cleavage of the carbon–oxygen bond in the anhydride. We therefore conclude that both the inner‐sphere substitution shown in Figure 1 and the dissociative pathway represent limiting mechanistic scenarios of a process that most likely involves an acylium ion bound either to the FeCl_3_–OAc complex or to acetic anhydride solvent molecules.

## 3 Conclusion

In conclusion, we have developed a mild and sustainable variant of the Birkofer protocol for alkynone synthesis, replacing stoichiometric AlCl_3_ and corrosive acid chlorides with catalytic FeCl_3_ and acetic anhydride. This approach complements the contributions of Yadav and Cheng and works particularly well with acetic anhydride. However, its extension to other anhydrides remains challenging. Further efforts are required to broaden the scope and reinforce the synthetic utility of this transformation. DFT studies of the mechanism delineated two mechanistic scenarios, with the actual pathway likely representing a combination of both.

## Supporting Information

The supporting information file includes additional optimization reactions, comprehensive characterization data, and NMR spectra for all relevant compounds.

## Conflict of Interest

The authors declare no conflict of interest.

## Supporting information

Supplementary Material

## Data Availability

The data that support the findings of this study are available in the supplementary material of this article.
