# Supplementary material for: FeCl3‐Catalyzed Synthesis of Ynones from Silylated Alkynes and Acetic Anhydride
Source: ChemistryOpen. 2025 Oct 27;15(4):e202500402. doi: 10.1002/open.202500402 (PMC13052263; doi:10.1002/open.202500402)

Table of contents

General Experimental Methods	S2
Reagents used	S3
Optimization reactions as described	S4
Determination of response factors	S5
Evaluation of Lewis acids	S7
Screening conditions for scandium triflate	S7
Screening conditions for iron chloride	S8
GC trace for deprotection of **1** in presence of acetic acid	S9
GC trace for (attempted) synthesis of compounds **41**	S10
Unreactive silylated alkynes	S11
Acetylation reactions between silylated alkynes and anhydride acid in presence of iron (III) chloride.	S11
Computational methods	S25
References	S32
NMR spectra	S34

■ General Experimental Methods

If not otherwise noted technical grade solvents were used (no pretreatment, degassing etc.) for the reactions. Iron chloride was directly used from the flask and weighed quickly due to its hydroscopic nature. Column chromatography separations were achieved on silica gel (40-63 μm). All Thin Layer Chromatographies (TLC) were performed on aluminium backed plates pre-coated with silica gel (Merck, Silica Gel 60 F254). They were visualized by exposure to UV light or stained with $KMnO_4$. ^1H and ^{13}C NMR spectra were recorded on a Bruker Avance 300 instrument. Chemical shifts (δ) are reported in part per million (ppm) relatively to TMS and residual solvent as internal standard. The following abbreviations are used for multiplicities: s, singlet; d, doublet; t, triplet; dd, doublet of doublets; td, triplet of doublets; m, multiplet and bs (broad singlet). Coupling constants (*J*) are reported in Hertz (Hz). HRMS analyses were obtained on a Bruker Maxis 4G instrument. Melting points were measured on a Stuart© (smp10) melting point apparatus. GC-traces were recorded on a Agilent 8890 instrument equipped with a HP-5 column (30 m × 320 μm × 0.25 μm) as stationary phase. The following temperature protocol was used: 70 °C initial temperature (Hold time 3 min), Ramp 1 (10 °C/ min) from 70 -130 °C, then Ramp 2 (20 °C/ min) from 130 °C - 300 °C (Hold time 3 minutes). Flow rate (H_2) = 3.5 mL / min; Average velocity = 63.72 cm / sec.

■ Reagents used

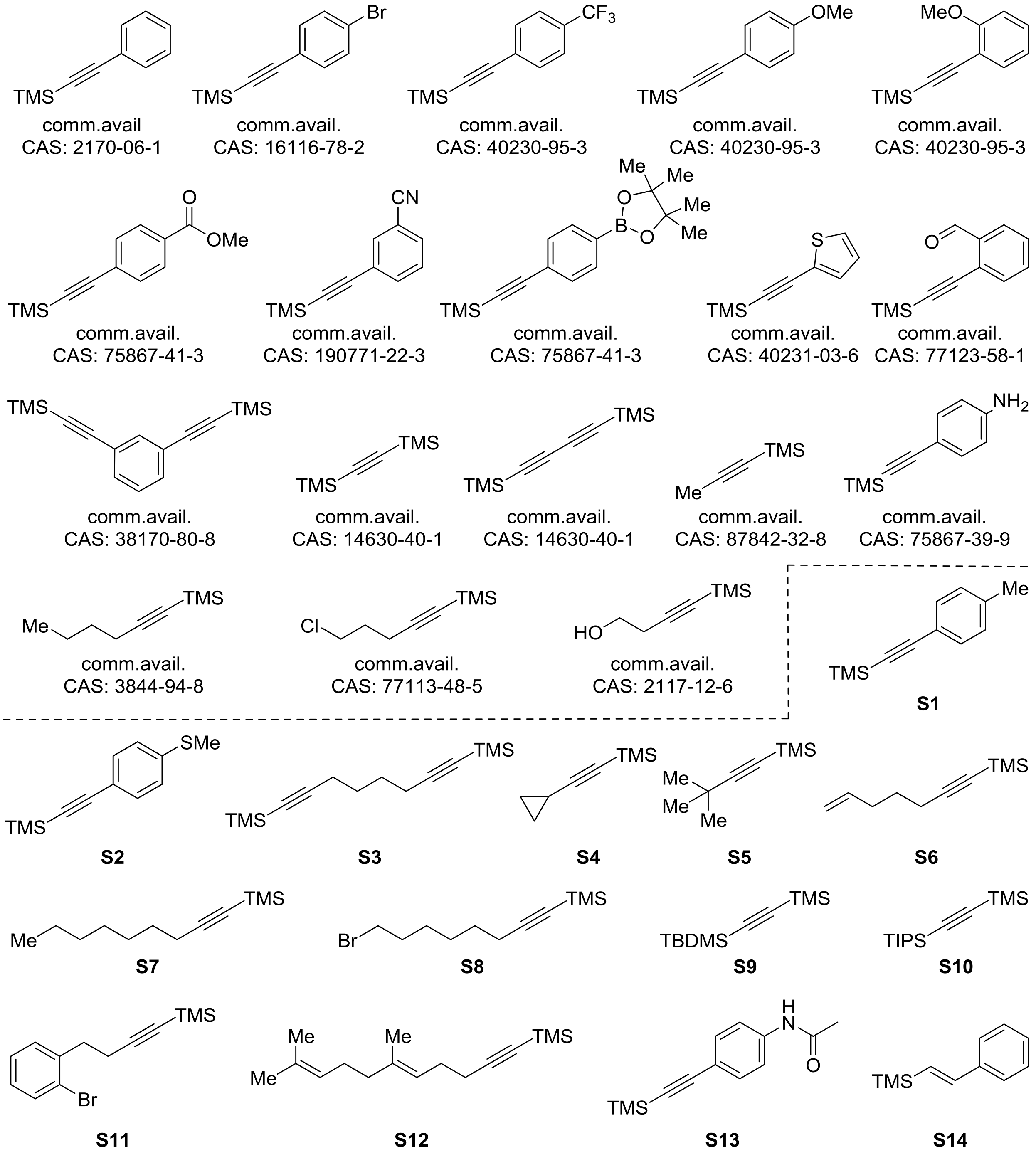

Figure S1: Reagents used.

Compounds **S1-6**,[1] **S7-8**,[2] **S9-10**,[3] **S11**[4], **S12**[5], **S13**[6] and **S14**[7] were prepared according to the literature.

■ Optimization reactions as described

With $Sc(OTf)_3$: A 4 mL vial equipped with a stirring bar was charged with 1-phenyl-2-trimethylsilylacetylene (**1**) (200 µL, 174 mg, 1.00 mmol, 1.00 equiv) and acetic anhydride (**2**) (190 µL, 204 mg, 2.00 mmol 2.00 equiv) and finally nitromethane (2.0 mL). $Sc(OTf_3)$ is added in one time and the reaction is heated to 50 °C in a heating block and stirred for 4 hours. Then the reaction was treated with H_2O (2 mL), transferred in a test tube and a solution on *n*-decane in toluene (1.0 mL, 0.5 M, 0.50 mmol, 0.50 equiv) is added. The crude reaction was analyzed by GC.

Note: When other temperatures where tried a cryocooler allowed to reach -15 °C and for 0 °C an ice bath was used.

With $FeCl_3$: A 4 mL vial equipped with a stirring bar was charged with 1-phenyl-2-trimethylsilylacetylene (**1**) (200 µL, 174 mg, 1.00 mmol, 1.00 equiv) and acetic anhydride (**2**) (950 µL, 1.02 g, 10.0 mmol 10.0 equiv). $FeCl_3$ is added in one time and the reaction is stirred at 20 °C for 4 hours. Then the reaction was treated with H_2O (2 mL), transferred in a test tube and a solution on *n*-decane in toluene (1.0 mL, 0.5 M, 0.50 mmol, 0.50 equiv) is added. The crude reaction was analyzed by GC.

Note 1: When evaluating different temperatures, a cryocooler was employed to achieve –15 °C, and an ice bath was utilized to maintain 0 °C.

Note 2: $FeCl_3$ was used directly from the flask without purification. Weighing and addition were completed within less than one minute to prevent hydrolysis, which was observed easily by a color change from black to a brown solid, accompanied by yellow droplets, as depicted in pictures A and B. (Figure S2).

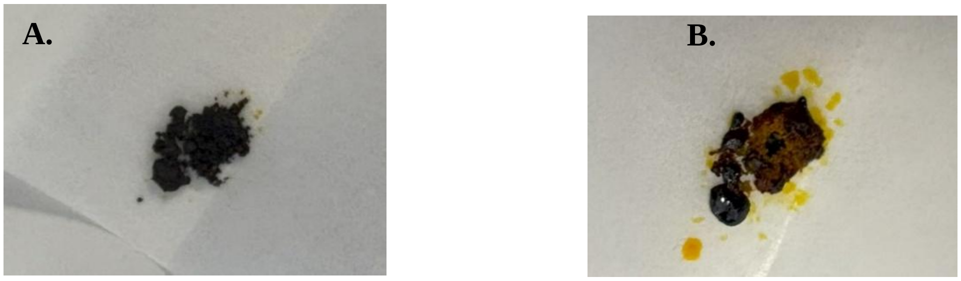

Figure S2. A. Picture of freshly weighed $FeCl_3$. **B.** Picture of $FeCl_3$ 5 mins after weighing.

■ Determination of response factors

1-phenyl-2-trimethylsilylacetylene (**1**)

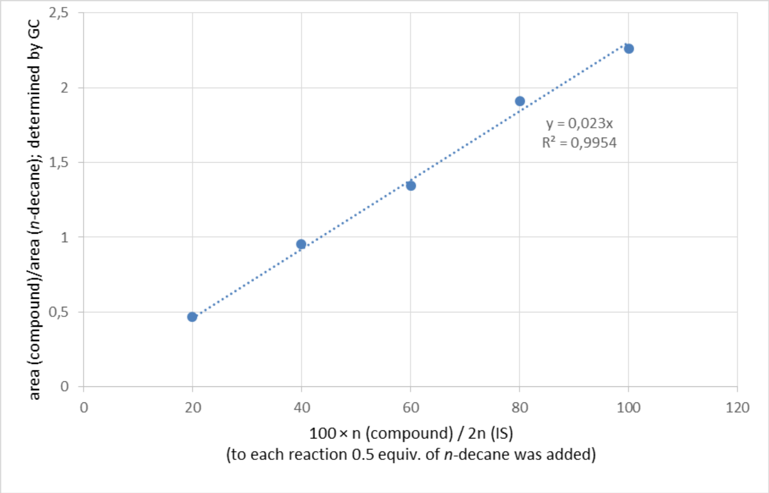

The GC conversion and yield were calculated according to the following equations with *n*-decane (0.5 equiv.) as external standard (ES) in comparison with the product **1**.

$$\text{Yield of } \mathbf{1} = \frac{\text{area of } \mathbf{1} \text{ / area of } n\text{-decane}}{0.023}$$

4-phenylbut-3-yn-2-one (**3**)

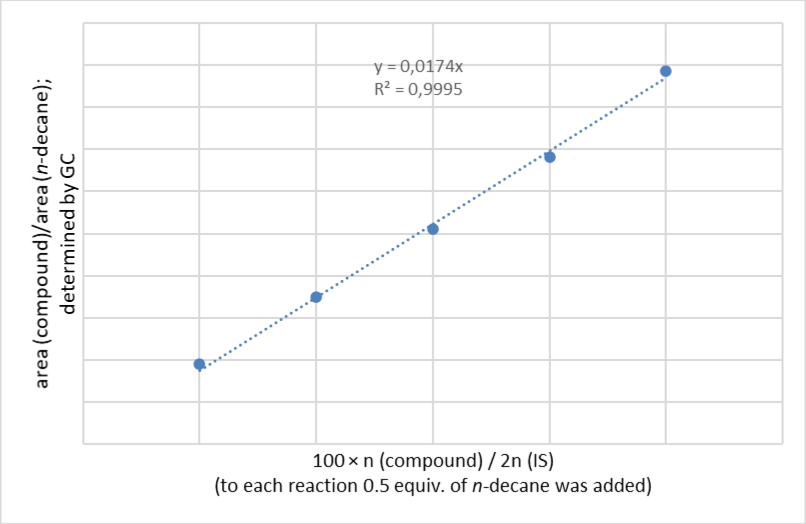

The GC conversion and yield were calculated according to the following equations with *n*-decane (0.5 equiv.) as ES in comparison with the product **3**.

$$\text{Yield of } \mathbf{3} = \frac{\text{area of } \mathbf{3} \text{ / area of } n\text{-decane}}{0.0174}$$

Phenylacetylene (**4**)

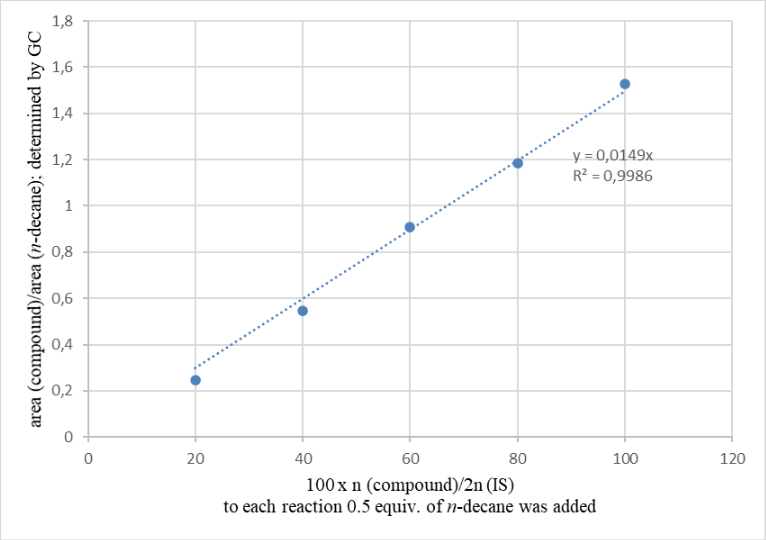

The GC conversion and yield were calculated according to the following equations with *n*-decane (0.5 equiv.) as ES in comparison with the product **4**.

$$\text{Yield of } \mathbf{4} = \frac{\text{area of } \mathbf{4} \text{ / area of } n\text{-decane}}{0.0149}$$

■ Evaluation of Lewis acids

Table S1: Additional Lewis acids tested.[a]

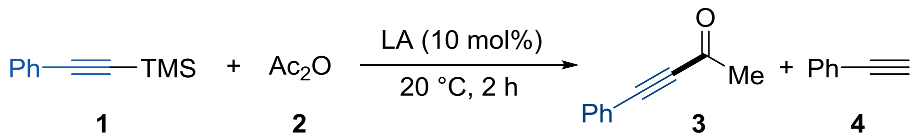

entry	Lewis acid	conv. **1** (%)[b]	yield **3** (%)[b]	yield **4** (%)[b]
1	$AlCl_3$	0	0	0
2	$Fe(OTf)_3$	97	54	14
3	$ZnCl_2$	57	50	3
4	$ZnBr_2$	60	40	8
5	$Zn(OTf)_2$	36	18	17
6	$TiCl_4$	0	0	0

[a]The reactions were performed on 1.0 mmol.

■ Screening conditions for scandium triflate

Table S2: Additional optimization with $Sc(OTf)_3$.[a]

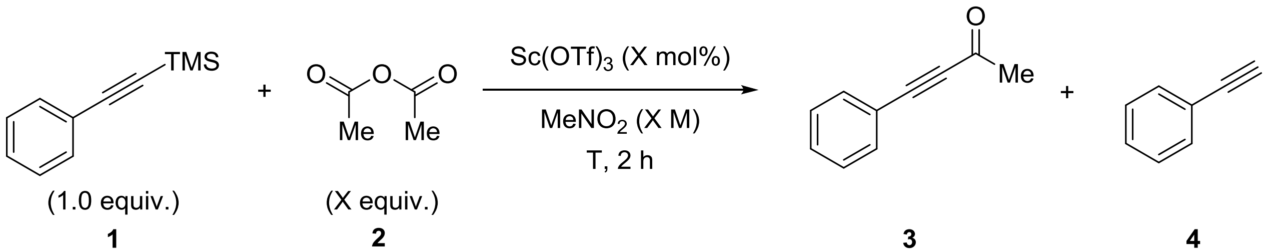

entry	X mol%	**2** equiv.	T (°C)	X M	conv. **1** (%)[b]	yield **3** (%)[b]	yield **4** (%)[b]
1	4.0	2.0	90	0.33	100	67	15
2	4.0	2.0	50	0.33	100	70	23
3	4.0	2.0	20	0.33	89	37	40
4	2.0	2.0	50	0.33	100	60	33
5	4.0	5.0	50	0.33	100	70	24
6[c]	4.0	2.0	50	0.33	100	0	73
7	4.0	2.0	50	1.0	100	67	23
8[d]	4.0	2.0	50	0.33	100	71	25

[a]The reactions were performed on 1.0 mmol. [b]Yield determined with *n*-decane as an external standard. [c]Addition de 2.0 equiv of LiO_4Cl. [d]Reaction done with anhydrous nitromethane.

■ **Screening conditions for iron chloride**

Table S3: Additional optimization with $FeCl_3$. [a]

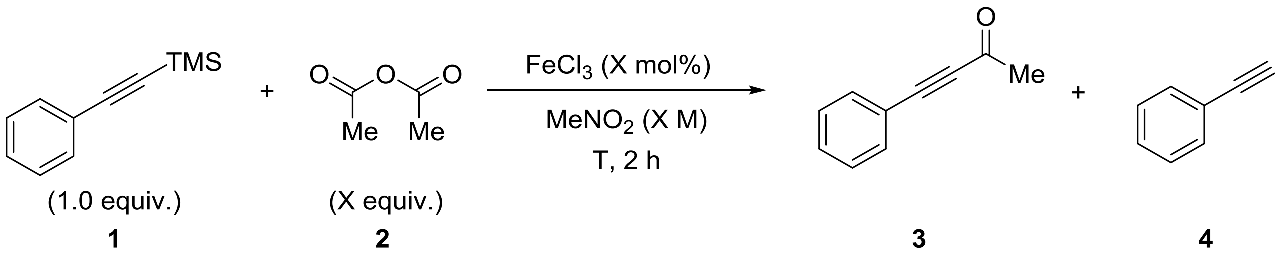

entry	X mol%	**2** equiv.	T (°C)	X M	conv. **1** (%)[b]	yield **3** (%)[b]	yield **4** (%)[b]
1[c]	10	1.0	-15	0.5	--	76	--
2	10	1.2	20	0.5	100	71	5
3	10	1.2	20	1.0	100	70	5
4	10	1.2	50	1.0	100	60	5
5	5	1.2	20	1.0	89	65	11
6	10	2.0	20	--	88	66	14
7	10	5.0	20	--	100	76	4
8	10	10.0	20	--	100	87	5
9[d]	10	10.0	20	--	100	86	7
10[d]	10	10.0	0	--	97	77	13

[a]The reactions were performed on 1.0 mmol. [b]Yield determined with decane as an external standard. [c]1.5 equiv of **1** was used and the reaction was stirred for 6 hours. [d]The reaction was stirred for 30 min.

■ GC trace for deprotection of 1 in presence of acetic acid

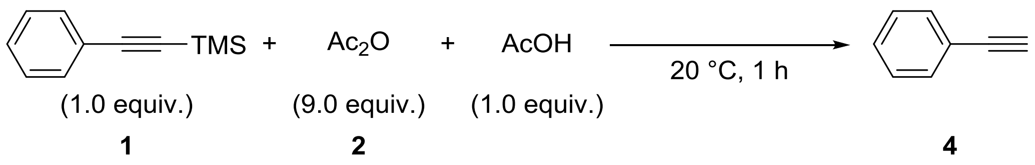

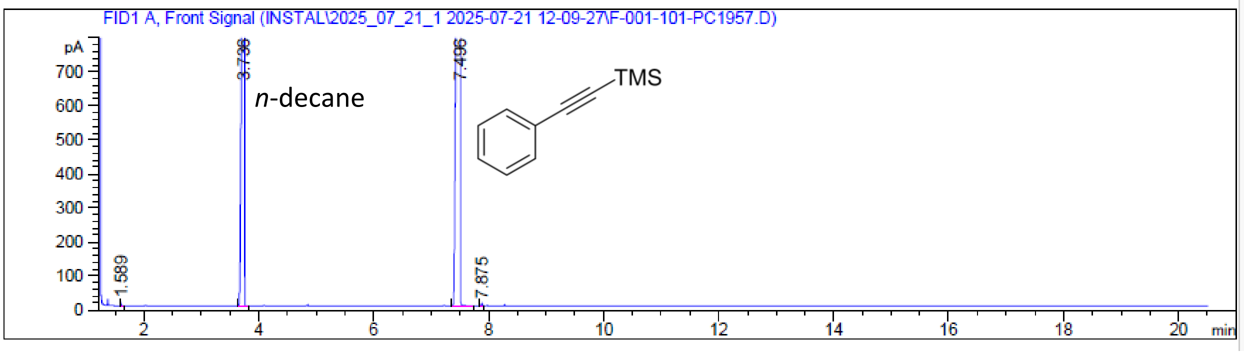

Peak #	RetTime [min]	Type	Width [min]	Area [pA*s]	Height [pA]	Area %
1	1.589	BB	0.0160	10.22960	9.94020	0.06235
2	3.736	BB	0.0366	5180.10449	1973.61560	31.57443
3	7.496	BB	0.0465	1.12059e4	3077.72021	68.30335
4	7.875	BB	0.0225	9.82272	6.96092	0.05987

■ GC trace for (attempted) synthesis of compounds 41

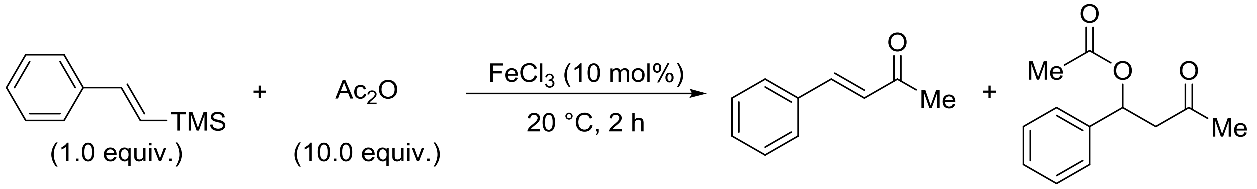

40 **2** **41** **42**

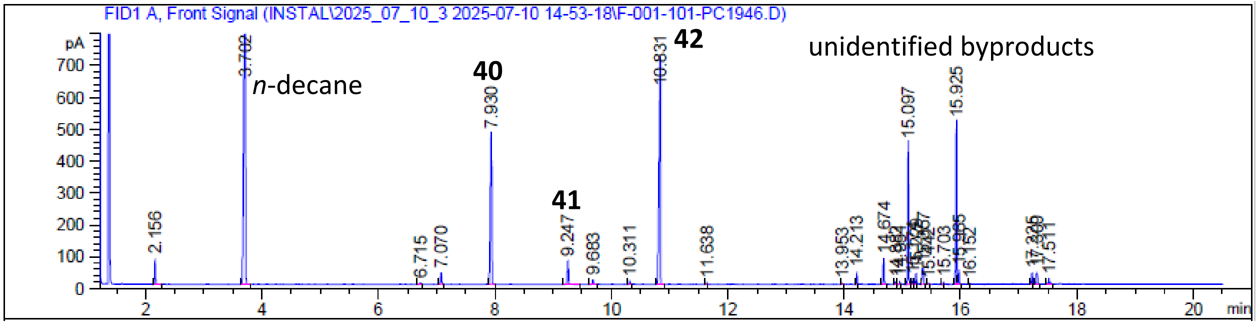

Peak #	RetTime [min]	Type	Width [min]	Area [pA*s]	Height [pA]	Area %
1	2.156	BB	0.0191	98.81248	81.48113	1.71261
2	3.702	BB	0.0276	2072.73120	1069.87903	35.92431
3	6.715	BB	0.0254	10.39170	6.58499	0.18011
4	7.070	BB	0.0237	56.86397	37.41356	0.98556
5	7.930	BB	0.0261	777.30200	475.36551	13.47210
6	9.247	BB	0.0234	110.91805	74.53409	1.92242
7	9.683	BB	0.0215	22.67230	16.05642	0.39295
8	10.311	BB	0.0182	13.57134	11.95893	0.23522
9	10.831	BB	0.0217	1093.55713	719.10541	18.95339
10	11.638	BB	0.0156	6.90905	6.97867	0.11975
11	13.953	BB	0.0168	5.10729	4.66293	0.08852
12	14.213	BB	0.0150	38.06642	40.38226	0.65976
13	14.674	BB	0.0155	81.88827	83.09129	1.41928
14	14.882	BB	0.0230	12.93499	9.43293	0.22419
15	14.951	BB	0.0153	10.67840	11.02737	0.18508
16	15.097	BB	0.0162	466.34305	445.49365	8.08260
17	15.174	BV	0.0167	19.74880	18.15417	0.34228
18	15.229	VB	0.0156	32.70257	32.84369	0.56680
19	15.357	BB	0.0316	100.11446	54.07122	1.73517
20	15.442	BB	0.0165	4.59987	4.67030	0.07972
21	15.703	BB	0.0155	9.34651	9.47564	0.16199
22	15.925	BB R	0.0162	530.73517	509.40637	9.19863
23	15.965	VB E	0.0160	47.24936	46.10960	0.81892
24	16.152	BB	0.0150	4.17206	4.42795	0.07231
25	17.225	BB	0.0175	38.32715	35.89023	0.66428
26	17.300	BB	0.0239	70.40350	39.36411	1.22022
27	17.511	BB	0.0252	33.57085	18.50787	0.58185

■ Unreactive silylated alkynes

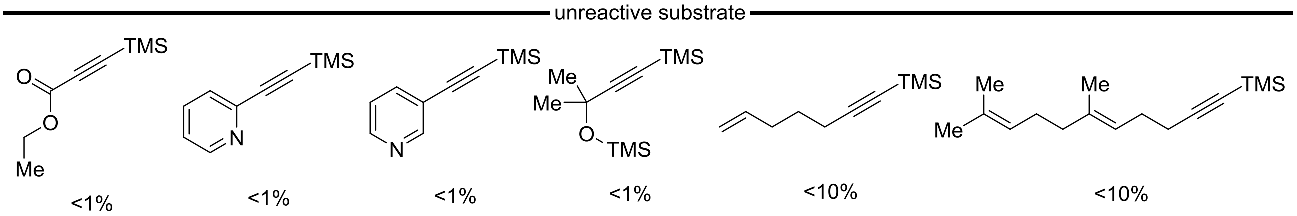

Scheme S1: Unreactive substrates

■ Acetylation reactions between silylated alkynes and anhydride acid in presence of iron (III) chloride.

4-phenylbut-3-yn-2-one (3): General procedure A

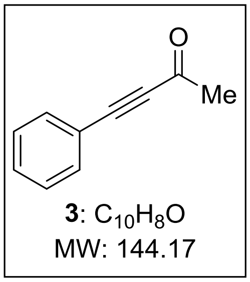

In a 4.0 mL vial were mixed trimethyl(phenylethynyl)silane (**1**) (197 µL, 174 mg, 1.00 mmol, 1.00 equiv) and anhydride acetic (**2**) (940 µL, 1.02 g, 10.0 mmol, 10.0 equiv). The mixture was stirred at 20 °C and $FeCl_3$ (16.2 mg, 0.10 mmol, 0.10 equiv) was added in one time. Upon addition the reaction turns from colorless to brown/black. The reaction was quenched after 30 min with H_2O (15 mL) and extracted with ether (3 × 15 mL). The organic phases were dried over $MgSO_4$ and concentrated *in vacuo*. The resultant residue was purified by flash column chromatography (pentane/ether : 19/1) to yield **3** as a yellow liquid (115 mg, 0.80 mmol, 80% yield).

R_f = 0.28 (pentane/ether : 19/1); **^{1}H NMR** (300 MHz, $CDCl_3$) δ: 7.60 – 7.54 (m, 2H), 7.50 – 7.35 (m, 3H), 2.46 (s, 3H); **^{13}C NMR** (75 MHz, $CDCl_3$) δ: 184.5, 133.0, 130.7, 128.6, 119.9, 90.3, 88.3, 32.7.

Spectral data were consistent with those previously reported.[8]

Note 1: Some substrates form a heterogeneous suspension initially due to polarity differences, but the mixture becomes a black homogeneous solution upon $FeCl_3$ addition.

Note 2: The reaction was conducted on a 15 mmol scale, yielding 79%. It is exothermic at this scale, reaching 40 °C, cooling with a water bath is recommended.

4-(4-chlorophenyl)but-3-yn-2-one (5)

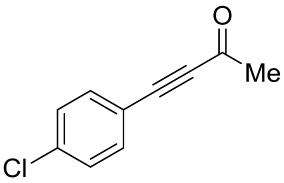

5: $C_{10}H_7ClO$
MW: 178.62

Synthesis according to general procedure A: ((4-chlorophenyl)ethynyl)trimethylsilane (209 mg, 1.00 mmol, 1.00 equiv), anhydride acetic (**2**) (940 µL, 1.02 g, 10.0 mmol, 10.0 equiv), $FeCl_3$ (16.2 mg, 0.10 mmol, 0.10 equiv), 30 min. The product was obtained after purification by column chromatography (pentane/Et_2O : 19/1) to yield **5** as a yellow solid (136 mg, 0.76 mmol, 76% yield).

$\mathbf{R}_{\boldsymbol{f}}$ = 0.31 (pentane/ether : 19/1); **^{1}H NMR** (300 MHz, $CDCl_3$) δ: 7.53 – 7.47 (m, 2H), 7.39 – 7.34 (m, 2H), 2.45 (s, 3H); **^{13}C NMR** (75 MHz, $CDCl_3$) δ: 184.2, 137.1, 134.2, 129.1, 118.4, 89.0, 88.7, 32.7.

Spectral data were consistent with those previously reported.[9]

4-(4-bromophenyl)but-3-yn-2-one (6)

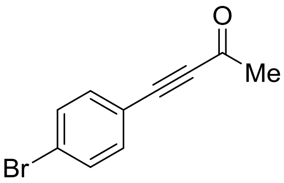

6: $C_{10}H_7BrO$
MW: 223.07

Synthesis according to general procedure A: ((4-bromophenyl)ethynyl)trimethylsilane (253 mg, 1.00 mmol, 1.00 equiv), anhydride acetic (**2**) (940 µL, 1.02 g, 10.0 mmol, 10.0 equiv), $FeCl_3$ (16.2 mg, 0.10 mmol, 0.10 equiv), 30 min. The product was obtained after purification by column chromatography (pentane/Et_2O : 19/1) to yield **6** as a yellow solid (182 mg, 81 mmol, 81% yield).

$\mathbf{R}_{\boldsymbol{f}}$ = 0.29 (pentane/ether : 19/1); **^{1}H NMR** (300 MHz, $CDCl_3$) δ: 7.56 – 7.50 (m, 2H), 7.45 – 7.39 (m, 2H), 2.45 (s, 3H); **^{13}C NMR** (75 MHz, $CDCl_3$) δ: 184.2, 134.3, 132.0, 125.5, 118.9, 89.1, 88.8, 32.7.

Spectral data were consistent with those previously reported.[10]

4-(4-(trifluoromethyl)phenyl)but-3-yn-2-one (7)

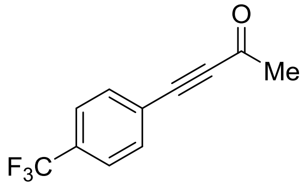

7: $C_{11}H_7F_3O$
MW: 212.17

Synthesis according to general procedure A: trimethyl((4-(trifluoromethyl)phenyl)ethynyl)silane (242 mg, 1.00 mmol, 1.00 equiv), anhydride acetic (**2**) (940 µL, 1.02 g, 10.0 mmol, 10.0 equiv), $FeCl_3$ (16.2 mg, 0.10 mmol, 0.10 equiv), 16 hours. The product was obtained after purification by column chromatography (pentane/Et_2O :19/1) to yield **7** as a yellow liquid (155 mg,

0.73 mmol, 73 % yield).

R*f* = 0.28 (pentane/ether : 19/1); **^{1}H NMR** (300 MHz, $CDCl_3$) δ: 7.74 – 7.62 (m, 4H), 2.47 (s, 3H); **^{13}C NMR** (75 MHz, $CDCl_3$) δ:184.2, 133.2, 132.3 (q, *J* = 33.0 Hz), 125.7 (q, *J* = 3.8 Hz), 123.9, 123.7 (q, *J* = 272.6 Hz), 89.5, 87.7, 32.8; **^{19}F NMR** (282 MHz, $CDCl_3$) δ: -63.20

Spectral data were consistent with those previously reported.[9]

4-(cyclohex-1-en-1-yl)but-3-yn-2-one (8)

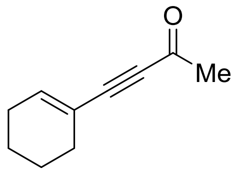

8: $C_{10}H_{12}O$
MW: 148.20

Synthesis according to general procedure A: (cyclohex-1-en-1-ylethynyl)trimethylsilane (178 mg, 1.00 mmol, 1.00 equiv), anhydride acetic (**2**) (940 µL, 1.02 g, 10.0 mmol, 10.0 equiv), $FeCl_3$ (16.2 mg, 0.10 mmol, 0.10 equiv), 16 hours. The product was obtained after purification by column chromatography (pentane/Et_2O : 19/1) to yield **8** as a yellow liquid (80 mg, 0.54 mmol, 54 % yield).

R*f* = 0.29 (pentane/ether : 19/1); **^{1}H NMR** (300 MHz, $CDCl_3$) δ: 6.45 (p, *J* = 2.1 Hz, 1H), 2.35 (s, 3H), 2.21 – 2.12 (m, 4H), 1.72 – 1.57 (m, 4H); **^{13}C NMR** (75 MHz, $CDCl_3$) δ: 184.6, 142.3, 118.9, 92.8, 86.7, 32.6, 28.3, 26.1, 21.9, 21.1.

Spectral data were consistent with those previously reported.[11]

4-(p-tolyl)but-3-yn-2-one (9)

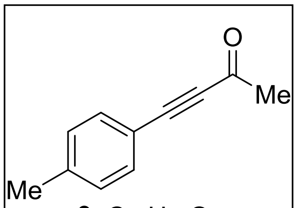

9: $C_{11}H_{10}O$
MW: 158.20

Synthesis according to general procedure A: trimethyl(p-tolylethynyl)silane (184 mg, 1.00 mmol, 1.00 equiv), anhydride acetic (**2**) (940 µL, 1.02 g, 10.0 mmol, 10.0 equiv), $FeCl_3$ (16.2 mg, 0.10 mmol, 0.10 equiv), 30 min. The product was obtained after purification by column chromatography (pentane/Et_2O : 19/1) to yield **9** as a yellow liquid (116 mg, 0.73 mmol, 73 % yield).

R*f* = 0.30 (pentane/ether : 19/1); **^{1}H NMR** (300 MHz, $CDCl_3$) δ: 7.50 – 7.44 (m, 2H), 7.22 – 7.17 (m, 2H), 2.44 (s, 3H), 2.39 (s, 3H); **^{13}C NMR** (75 MHz, $CDCl_3$) δ: 184.6, 141.5, 133.2, 129.5, 116.9, 91.1, 88.3, 32.8, 21.8.

Spectral data were consistent with those previously reported.[10]

4-(4-methoxyphenyl)but-3-yn-2-one (10)

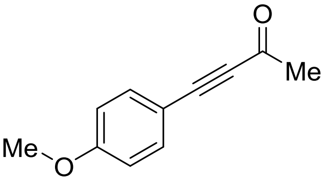

10: $C_{11}H_{10}O_2$
MW: 174.20

Synthesis according to general procedure A: ((4-methoxyphenyl)ethynyl)trimethylsilane (204 mg, 1.00 mmol, 1.00 equiv), anhydride acetic (**2**) (940 µL, 1.02 g, 10.0 mmol, 10.0 equiv), $FeCl_3$ (16.2 mg, 0.10 mmol, 0.10 equiv), 16 hours. The product was obtained after purification by column chromatography (pentane/Et_2O : 9/1) to yield **10** as a yellow liquid (86 mg, 0.49 mmol, 49% yield).

R*f* = 0.28 (pentane/ether : 9/1); **^{1}H NMR** (300 MHz, $CDCl_3$) δ: 7.54 – 7.45 (m, 2H), 6.92 – 6.82 (m, 2H), 3.82 (s, 3H), 2.41 (s, 3H); **^{13}C NMR** (75 MHz, $CDCl_3$) δ: 184.6, 161.8, 135.2, 114.5, 111.8, 91.5, 88.3, 55.5, 32.7.

Spectral data were consistent with those previously reported.[10]

4-(2-methoxyphenyl)but-3-yn-2-one (11)

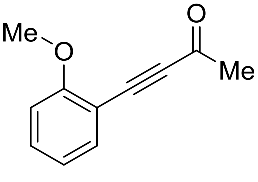

11: $C_{11}H_{10}O_2$
MW: 174.20

Synthesis according to general procedure A: ((2-methoxyphenyl)ethynyl)trimethylsilane (204 mg, 1.00 mmol, 1.00 equiv), anhydride acetic (**2**) (940 µL, 1.02 g, 10.0 mmol, 10.0 equiv), $FeCl_3$ (16.2 mg, 0.10 mmol, 0.10 equiv), 16 hours. The product was obtained after purification by column chromatography (pentane/Et_2O : 9/1) to yield **11** as a yellow liquid (92 mg, 0.52 mmol, 52% yield).

R*f* = 0.27 (pentane/ether : 9/1); **^{1}H NMR** (300 MHz, $CDCl_3$) δ: 7.54 – 7.45 (m, 2H), 6.92 – 6.82 (m, 2H), 3.82 (s, 3H), 2.41 (s, 3H); **^{13}C NMR** (75 MHz, $CDCl_3$) δ: 184.6, 161.5, 135.0, 132.5, 120.6, 111.0, 109.2, 92.5, 87.7, 55.9, 32.8.

Spectral data were consistent with those previously reported.[10]

4-(4-(methylthio)phenyl)but-3-yn-2-one (12)

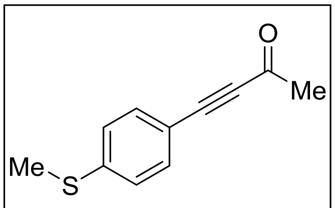

12: $C_{11}H_{10}OS$
MW: 190.26

Synthesis according to general procedure A: trimethyl((4-(methylthio)phenyl)ethynyl)silane (220 mg, 1.00 mmol, 1.00 equiv), anhydride acetic (**2**) (940 µL, 1.02 g, 10.0 mmol, 10.0 equiv), $FeCl_3$ (32.4 mg, 0.20 mmol, 0.20 equiv), 40 hours. The product was obtained after purification by column chromatography

(pentane/Et_2O : 9/1) to yield **12** as a yellowish solid (90 mg, 0.47 mmol, 47% yield).

R_f = 0.28 (pentane/ether : 9/1); **^{1}H NMR** (300 MHz, $CDCl_3$) δ: 7.52 – 7.44 (m, 2H), 7.24 – 7.18 (m, 2H), 2.50 (s, 3H), 2.44 (s, 3H); **^{13}C NMR** (75 MHz, $CDCl_3$) δ: 184.6, 143.5, 133.5, 125.6, 115.8, 90.7, 88.9, 32.8, 15.0.

Spectral data were consistent with those previously reported.[12]

4-(thiophen-2-yl)but-3-yn-2-one (13)

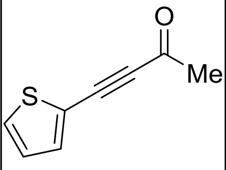

13: C_8H_6OS
MW: 150.20

Synthesis according to general procedure A: trimethyl(thiophen-2-ylethynyl)silane (180 mg, 1.00 mmol, 1.00 equiv), anhydride acetic (**2**) (940 µL, 1.02 g, 10.0 mmol, 10.0 equiv), $FeCl_3$ (16.2 mg, 0.10 mmol, 0.10 equiv), 16 hours. The product was obtained after purification by column chromatography (pentane/Et_2O : 9/1) to yield **13** as a brown liquid (59 mg, 0.39 mmol, 39% yield).

R_f = 0.33 (pentane/ether : 9/1); **^{1}H NMR** (300 MHz, $CDCl_3$) δ: 7.50 – 7.43 (m, 2H), 7.09 – 7.01 (m, 1H), 2.41 (s, 3H); **^{13}C NMR** (75 MHz, $CDCl_3$) δ: 184.0, 136.7, 131.7, 127.7, 119.7, 92.9, 84.3, 32.4.

Spectral data were consistent with those previously reported.[13]

methyl 4-(3-oxobut-1-yn-1-yl)benzoate (14)

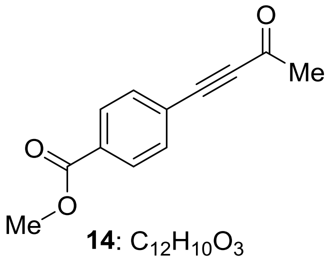

14: $C_{12}H_{10}O_3$
MW: 202.21

Synthesis according to general procedure A: methyl 4-((trimethylsilyl)ethynyl)benzoate (232 mg, 1.00 mmol, 1.00 equiv), anhydride acetic (**2**) (940 µL, 1.02 g, 10.0 mmol, 10.0 equiv), $FeCl_3$ (32.4 mg, 0.20 mmol, 0.20 equiv), 40 hours. The product was obtained after purification by column chromatography (pentane/Et_2O : 8/2) to yield **14** as a yellow solid (140 mg, 0.69 mmol, 69% yield).

R_f = 0.30 (pentane/ether : 8/2); **^{1}H NMR** (300 MHz, $CDCl_3$) δ: 8.07 – 8.03 (m, 2H), 7.65 – 7.61 (m, 2H), 3.94 (s, 3H), 2.47 (s, 3H); **^{13}C NMR** (75 MHz, $CDCl_3$) δ: 184.3, 166.1, 132.9, 131.8, 129.7, 124.5, 89.9, 88.5, 52.5, 32.8.

Spectral data were consistent with those previously reported.[9]

3-(3-oxobut-1-yn-1-yl)benzonitrile (15)

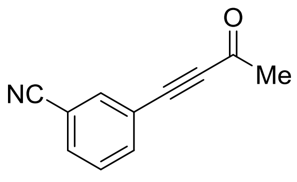

15: $C_{11}H_7NO$
MW: 169,18

Synthesis according to general procedure A: 3-((trimethylsilyl)ethynyl)benzonitrile (199 mg, 1.00 mmol, 1.00 equiv), anhydride acetic (**2**) (940 µL, 1.02 g, 10.0 mmol, 10.0 equiv), $FeCl_3$ (16.2 mg, 0.10 mmol, 0.10 equiv), 16 hours. The product was obtained after purification by column chromatography (pentane/Et_2O : 3/2) to yield **15** as a white solid (125 mg, 0.74 mmol, 74% yield).

$\mathbf{R}_f$ = 0.32 (pentane/ether : 3/2); **^{1}H NMR** (300 MHz, $CDCl_3$) δ: 7.77 – 7.74 (m, 1H), 7.74 – 7.63 (m, 2H), 7.52 – 7.44 (m, 1H), 2.40 (s, 3H); **^{13}C NMR** (75 MHz, $CDCl_3$) δ: 183.7, 136.6, 135.8, 133.6, 129.7, 121.5, 117.3, 113.2, 89.2, 86.3, 32.6. **HRMS** (ASAP) m/z calcd for C_{11} H_8 N O, $[M + H]^+$ 170.0600, found 170.0601, **m. p.** 78-80 °C.

4-(4-(4,4,5,5-tetramethyl-1,3,2-dioxaborolan-2-yl)phenyl)but-3-yn-2-one (16) and 2-(4-ethynylphenyl)-4,4,5,5-tetramethyl-1,3,2-dioxaborolane (51)

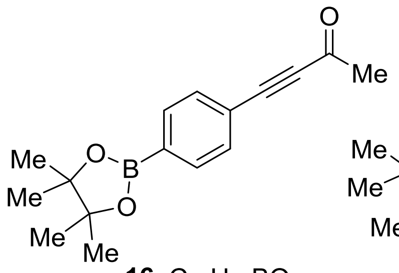

16: $C_{16}H_{19}BO_3$
MW: 270.14

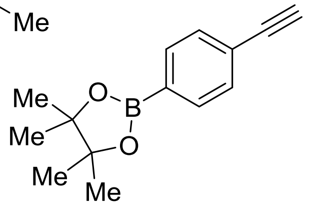

51: $C_{14}H_{17}BO_2$
M.W.: 228.10

Synthesis according to general procedure A: trimethyl((4-(4,4,5,5-tetramethyl-1,3,2-dioxaborolan-2-yl)phenyl)ethynyl)-silane (300 mg, 1.00 mmol, 1.00 equiv), anhydride acetic (940 µL, 1.02 g, 10.0 mmol, 10.0 equiv), $FeCl_3$ (16.2 mg, 0.10 mmol, 0.10 equiv), 16 hours. The two products were obtained after purification by column chromatography (Gradient pentane/Et_2O : 19/1 -> 9/1) to yield **16** as a yellowish liquid (100 mg, 0.37 mmol, 37% yield) and **51** as a colorless liquid (47.6 mg, 21 mmol, 21%) .

161: $\mathbf{R}_f$ = 0.29 (pentane/ether : 9/1); **^{1}H NMR** (300 MHz, $CDCl_3$) δ: 7.83 – 7.78 (m, 2H), 7.58 – 7.53 (m, 2H), 2.45 (s, 3H), 1.35 (s, 12H); **^{13}C NMR** (75 MHz, $CDCl_3$) δ: 184.5, 134.8, 132.1, 122.5, 90.2, 89.1, 84.3, 32.8, 25.0; **^{11}B NMR** (96 MHz, $CDCl_3$) δ: 31.36.

51 $\mathbf{R}_f$ = 0.35 (pentane/ether : 19/1); **^{1}H NMR** (300 MHz, $CDCl_3$) δ: 7.81 – 7.70 (m, 2H), 7.54 – 7.44 (m, 2H), 3.14 (s, 1H), 1.34 (s, 12H); **^{13}C NMR** (75 MHz, $CDCl_3$) δ: 134.6, 131.4, 124.8, 84.1, 83.8, 78.5, 24.9; **^{11}B NMR** (96 MHz, $CDCl_3$) δ: 30.48.

16 and **51** are known compounds.[14,15]

4,4'-(1,3-phenylene)bis(but-3-yn-2-one) (17)

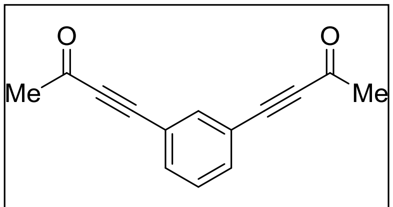

17: $C_{14}H_{10}O_2$
MW: 210.23

Synthesis according to general procedure A: 1,3-bis((trimethylsilyl)ethynyl)benzene (270 mg, 1.00 mmol, 1.00 equiv), anhydride acetic (**2**) (940 µL, 1.02 g, 10.0 mmol, 10.0 equiv), $FeCl_3$ (32.4 mg, 0.20 mmol, 0.20 equiv), 2 hours. The product was obtained after purification by column chromatography (pentane/Et_2O : 3/2) to yield **17** as a yellowish liquid (105 mg, 0.50 mmol, 50% yield).

$\mathbf{R}_{\boldsymbol{f}}$ = 0.31 (pentane/ether : 3/2); ^{1}**H NMR** (300 MHz, $CDCl_3$) δ: 7.78 – 7.71 (m, 1H), 7.69 – 7.56 (m, 2H), 7.47 – 7.34 (m, 1H), 2.45 (s, 5H); ^{13}**C NMR** (75 MHz, $CDCl_3$) δ: 184.4, 137.0, 134.8, 129.2, 120.9, 88.8, 88.0, 32.9.

Spectral data were consistent with those previously reported.[16]

dodeca-3,9-diyne-2,11-dione (18)

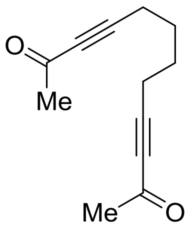

18: $C_{12}H_{14}O_2$
MW: 190.24

Synthesis according to general procedure A: 1,8-bis(trimethylsilyl)octa-1,7-diyne (250 mg, 1.00 mmol, 1.00 equiv), anhydride acetic (**2**) (940 µL, 1.02 g, 10.0 mmol, 10.0 equiv), $FeCl_3$ (32.4 mg, 0.20 mmol, 0.20 equiv), 16 hours. The product was obtained after purification by column chromatography (pentane/Et_2O : 3/2) to yield **18** as a yellow liquid (120 mg, 0.63 mmol, 63 % yield).

$\mathbf{R}_{\boldsymbol{f}}$ = 0.30 (pentane/ether : 3/2); ^{1}**H NMR** (300 MHz, $CDCl_3$) δ: 2.43 – 2.39 (m, 4H), 2.32 (s, 6H), 1.75 – 1.68 (m, 4H); ^{13}**C NMR** (75 MHz, $CDCl_3$) δ: 184.5, 92.6, 81.6, 32.6, 26.5, 18.3.

Spectral data were consistent with those previously reported.[17]

4-(trimethylsilyl)but-3-yn-2-one (19)

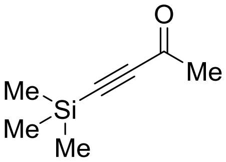

19: $C_7H_{12}OSi$
MW: 140.26

Synthesis according to general procedure A: 1,2-bis(trimethylsilyl)ethyne (170 mg, 1.00 mmol, 1.00 equiv), anhydride acetic (**2**) (940 µL, 1.02 g, 10.0 mmol, 10.0 equiv), $FeCl_3$ (16.2 mg, 0.10 mmol, 0.10 equiv), 1 hour. The organic phase was extracted with pentane. The desired product was obtained after purification by column chromatography (pentane/Et_2O : 19/1)

to yield **19** as a yellowish liquid (102 mg, 0.73 mmol, 73% yield).

R*f* = 0.40 (pentane/ether : 19/1); **¹H NMR** (300 MHz, $CDCl_3$) δ: 2.34 (s, 3H), 0.24 (s, 9H); **¹³C NMR** (75 MHz, $CDCl_3$) δ: 184.3, 102.5, 97.3, 32.5, -0.7.

Spectral data were consistent with those previously reported.[18]

4-(*tert*-butyldimethylsilyl)but-3-yn-2-one (20)

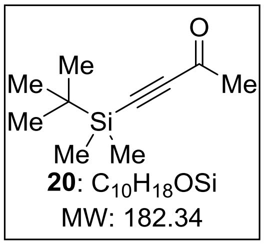

20: $C_{10}H_{18}OSi$
MW: 182.34

Synthesis according to general procedure A: *tert*-butyldimethyl((trimethylsilyl)ethynyl)silane (212 mg, 1.00 mmol, 1.00 equiv), anhydride acetic (**2**) (940 µL, 1.02 g, 10.0 mmol, 10.0 equiv), $FeCl_3$ (32.4 mg, 0.20 mmol, 0.20 equiv), 40 hours. The organic phase was extracted with pentane. The product was obtained after purification by column chromatography (pentane/Et_2O : 3/2) to yield **20** as a yellowish liquid (110 mg, 0.60 mmol, 60% yield).

R*f* = 0.39 (pentane/ether : 3/2); **¹H NMR** (300 MHz, $CDCl_3$) δ: 2.35 (s, 3H), 0.96 (s, 9H), 0.18 (s, 6H); **¹³C NMR** (75 MHz, $CDCl_3$) δ: 184.4, 103.3, 96.3, 32.7, 26.0, 16.6, -5.1.

Spectral data were consistent with those previously reported.[19]

4-(triisopropylsilyl)but-3-yn-2-one (21)

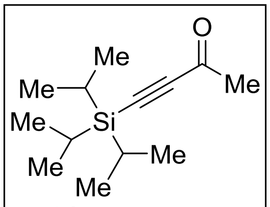

21: $C_{13}H_{24}OSi$
MW: 224.42

Synthesis according to general procedure A: triisopropyl((trimethylsilyl)ethynyl)silane (25 mg, 1.00 mmol, 1.00 equiv), anhydride acetic (**2**) (940 µL, 1.02 g, 10.0 mmol, 10.0 equiv), $FeCl_3$ (32.4 mg, 0.20 mmol, 0.20 equiv), 40 hours. The organic phase was extracted with pentane. The product was obtained after purification by column chromatography (pentane/Et_2O : 19/1) to yield **21** as a yellowish liquid (106 mg, 0.47 mmol, 47% yield).

R*f* = 0.38 (pentane/ether : 19/1); **¹H NMR** (300 MHz, $CDCl_3$) δ: 2.36 (s, 3H), 1.14 – 1.08 (m, 21H); **¹³C NMR** (75 MHz, $CDCl_3$) δ: 184.3, 104.9, 95.2, 32.9, 18.6, 11.1.

Spectral data were consistent with those previously reported.[20]

6-(trimethylsilyl)hexa-3,5-diyn-2-one (22)

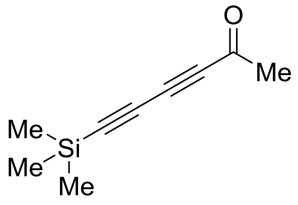

22: $C_9H_{12}OSi$
MW: 164.28

Synthesis according to general procedure A: 1,4-bis(trimethylsilyl)buta-1,3-diyne (194 mg, 1.00 mmol, 1.00 equiv), anhydride acetic (**2**) (940 µL, 1.02 g, 10.0 mmol, 10.0 equiv), $MeNO_2$ (2 mL), $FeCl_3$ (32.4 mg, 0.20 mmol, 0.20 equiv), 40 hours. The organic phase was extracted with pentane. The product was obtained after purification by column chromatography (pentane/Et_2O : 3/2) to yield **22** as a yellowish liquid (55.3 mg, 0.34 mmol, 34% yield).

R*f* = 0.40 (pentane/ether : 3/2); **^{1}H NMR** (300 MHz, $CDCl_3$) δ: 2.35 (s, 3H), 0.96 (s, 9H), 0.18 (s, 6H); **^{13}C NMR** (75 MHz, $CDCl_3$) δ: 184.4, 103.3, 96.3, 32.7, 26.0, 16.6, -5.1.

Spectral data were consistent with those previously reported.[21]

5,5-dimethylhex-3-yn-2-one (23)

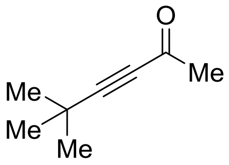

23: $C_8H_{12}O$
MW: 124.18

Synthesis according to general procedure A: (3,3-dimethylbut-1-yn-1-yl)trimethylsilane (154 mg, 1.00 mmol, 1.00 equiv), anhydride acetic (**2**) (940 µL, 1.02 g, 10.0 mmol, 10.0 equiv), $FeCl_3$ (16.2 mg, 0.10 mmol, 0.10 equiv), 30 min. After 30 min, the reaction was cooled to 0 °C using an ice bath. The reaction was quenched with a NaOH (2.0 M, 12 mL). The aqueous phase was extracted with Et_2O (3 × 5 mL). The combined organic phases were washed with sat. aqueous sol. $NaHCO_3$ (10 mL) and once more with water. The organic phase was concentrated (50 °C, 1 atm). The product was obtained after purification by column chromatography (pentane/Et_2O : 19/1) to yield **23** as a yellow liquid (90 mg, 0.72 mmol, 72% yield).

R*f* = 0.29 (pentane/ether : 19/1); **^{1}H NMR** (300 MHz, $CDCl_3$) δ: 2.31 (s, 3H), 1.28 (s, 9H); **^{13}C NMR** (75 MHz, $CDCl_3$) δ: 185.2, 101.2, 80.0, 32.9, 30.2, 27.8.

Spectral data were consistent with those previously reported.[22]

oct-3-yn-2-one (24)

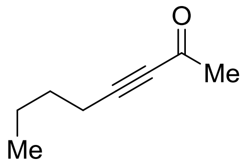

24: $C_8H_{12}O$
MW: 124.18

Synthesis according to general procedure A: hex-1-yn-1-yltrimethylsilane (200 µL, 154 mg, 1.00 mmol, 1.00 equiv), anhydride acetic (**2**) (940 µL, 1.02 g, 10.0 mmol, 10.0 equiv), $FeCl_3$ (16.2 mg, 0.10 mmol, 0.10 equiv), 30 min. The product was obtained after purification by column chromatography (pentane/Et_2O : 19/1) to yield **24** as a yellow solid (96 mg, 0.77 mmol, 77% yield).

R*f* = 0.29 (pentane/ether : 19/1); **^{1}H NMR** (300 MHz, $CDCl_3$) δ: 2.36 (t, *J* = 7.0 Hz, 2H), 2.32 (s, 3H), 1.62 – 1.51 (m, 2H), 1.49 – 1.38 (m, 2H), 0.93 (t, *J* = 7.2 Hz, 3H); **^{13}C NMR** (75 MHz, $CDCl_3$) δ: 184.7, 93.9, 81.4, 32.7, 29.7, 21.9, 18.5, 13.4.
Spectral data were consistent with those previously reported.[23]

undec-3-yn-2-one (25)

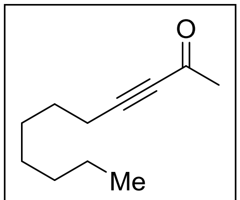

26: $C_{11}H_{18}O$
MW: 166.26

Synthesis according to general procedure A: trimethyl(non-1-yn-1-yl)silane (196 mg, 1.00 mmol, 1.00 equiv), anhydride acetic (**2**) (940 µL, 1.02 g, 10.0 mmol, 10.0 equiv), $FeCl_3$ (16.2 mg, 0.10 mmol, 0.10 equiv), 30 min. The product was obtained after purification by column chromatography (pentane/Et_2O : 19/1) to yield **26** as a yellow solid (131 mg, 79 mmol, 79% yield).

R*f* = 0.29 (pentane/ether : 19/1); **^{1}H NMR** (300 MHz, $CDCl_3$) δ: 2.35 (t, *J* = 7.1 Hz, 2H), 2.32 (s, 3H), 1.63 – 1.55 (m, 2H), 1.42 – 1.27 (m, 8H), 0.94 – 0.85 (m, 3H); **^{13}C NMR** (75 MHz, $CDCl_3$) δ: 184.7, 94.0, 81.4, 32.7, 31.6, 28.8, 28.7, 27.7, 22.6, 18.9, 14.0.
Spectral data were consistent with those previously reported.[24]

7-chlorohept-3-yn-2-one (26)

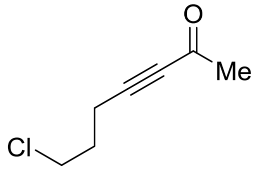

25: C_7H_9ClO
MW: 144.60

Synthesis according to general procedure A: (5-chloropent-1-yn-1-yl)trimethylsilane (175 mg, 1.00 mmol, 1.00 equiv), anhydride acetic (**2**) (940 µL, 1.02 g, 10.0 mmol, 10.0 equiv), $FeCl_3$ (16.2 mg, 0.10 mmol, 0.10 equiv), 30 min. After 30 min, the reaction was cooled to 0 °C using an ice bath. The reaction was quenched with a NaOH (2.0 M, 12 mL). The aqueous phase was extracted with Et_2O (3 × 5 mL). The combined

organic phases were washed with sat. aqueous sol. $NaHCO_3$ (10 mL) and once more with water. The organic phase was concentrated (50 °C, 1 atm). The product was obtained after purification by column chromatography (pentane/Et_2O : 9/1) to yield **25** as a yellow solid (107 mg, 0.74 mmol, 74% yield).

$\mathbf{R_f}$ = 0.32 (pentane/ether : 9/1); **^{1}H NMR** (300 MHz, $CDCl_3$) δ: 3.68 – 3.60 (m, 2H), 2.62 – 2.48 (m, 2H), 2.32 (s, 3H), 2.08 – 2.00 (m, 2H); **^{13}C NMR** (75 MHz, $CDCl_3$) δ: 184.5, 91.5, 81.9, 43.3, 32.7, 30.4, 16.3.

Spectral data were consistent with those previously reported.[25]

10-bromodec-3-yn-2-one (27)

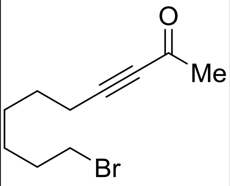

27: $C_{10}H_{15}BrO$
MW: 231.13

Synthesis according to general procedure A: (8-bromooct-1-yn-1-yl)trimethylsilane (203 mg, 0.78 mmol, 1.00 equiv), anhydride acetic (**2**) (740 µL, 796 mg, 7.80 mmol, 10.0 equiv), $FeCl_3$ (13.0 mg, 0.08 mmol, 0.08 equiv), 30 min. The product was obtained after purification by column chromatography (pentane/Et_2O : 9/1) to yield **27** as a yellow liquid (134 mg, 0.58 mmol, 74% yield).

$\mathbf{R_f}$ = 0.33 (pentane/ether : 9/1); **^{1}H NMR** (300 MHz, $CDCl_3$) δ: 3.41 (t, *J* = 6.7 Hz, 2H), 2.37 (t, *J* = 7.0 Hz, 2H), 2.32 (s, 3H), 1.94 – 1.82 (m, 2H), 1.65 – 1.57 (m, 2H), 1.51 – 1.41 (m, 4H); **^{13}C NMR** (75 MHz, $CDCl_3$) δ: 184.7, 93.6, 81.6, 33.7, 32.8, 32.5, 27.9, 27.6, 27.5, 18.8. **HRMS** (ASAP) m/z calcd for C_{10} H_{16} O ^{79}Br, $[M + H]^+$ 231.0379, found 231.0381.

6-(2-bromophenyl)hex-3-yn-2-one (28)

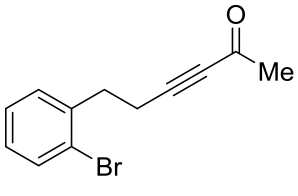

28: $C_{12}H_{11}BrO$
MW: 251.12

Synthesis according to general procedure A: (4-(2-bromophenyl)but-1-yn-1-yl)trimethylsilane (281 mg, 1.00 mmol, 1.00 equiv), anhydride acetic (**2**) (940 µL, 1.02 g, 10.0 mmol, 10.0 equiv), $FeCl_3$ (16.2 mg, 0.10 mmol, 0.10 equiv), 30 min. The product was obtained after purification by column chromatography (pentane/Et_2O : 9/1) to yield **28** as a yellowish liquid (194 mg, 0.77 mmol, 77% yield).

$\mathbf{R_f}$ = 0.33 (pentane/ether : 9/1); **^{1}H NMR** (300 MHz, $CDCl_3$) δ: 7.59 – 7.52 (m, 1H), 7.29 – 7.26 (m, 2H), 7.16 – 7.08 (m, 1H), 3.02 (t, *J* = 7.4 Hz, 2H), 2.69 (t, *J* = 7.4 Hz, 2H), 2.29 (s, 3H);

^{13}**C NMR** (75 MHz, $CDCl_3$) δ: 184.6, 138.7, 132.9, 130.7, 128.5, 127.5, 124.2, 92.2, 82.0, 34.2, 32.7, 19.2, **HRMS** (ASAP) m/z calcd for C_{12} H_{11} O ^{79}Br, $[M]^+$ 249.9987, found 249.9991.

pent-3-yn-2-one (29)

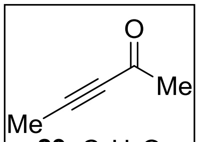

29: C_5H_6O
MW: 82.10

Synthesis according to general procedure A: trimethyl(prop-1-yn-1-yl)silane (112 mg, 1.00 mmol, 1.00 equiv), anhydride acetic (**2**) (940 µL, 1.02 g, 10.0 mmol, 10.0 equiv), $FeCl_3$ (16.2 mg, 0.10 mmol, 0.10 equiv), 30 min. After 30 min, the reaction was cooled to 0 °C using an ice bath. The reaction was quenched with a NaOH (2.0 M, 12 mL). The aqueous phase was extracted with Et_2O (3 × 5 mL). The combined organic phases were washed with sat. aqueous sol. $NaHCO_3$ (10 mL) and once more with water. The organic phase was concentrated (50 °C, 1 atm). The product was obtained after purification by column chromatography (pentane/Et_2O : 9/1) to yield **29** as a yellow liquid (38 mg, 0.46 mmol, 46% yield).

R*f* = 0.33 (pentane/ether : 9/1); ^{1}**H NMR** (300 MHz, $CDCl_3$) δ: 2.31 (s, 3H), 2.01 (s, 3H); ^{13}**C NMR** (75 MHz, $CDCl_3$) δ: 185.0, 89.9, 80.8, 32.7, 4.1.

Spectral data were consistent with those previously reported.[26]

4-cyclopropylbut-3-yn-2-one (30)

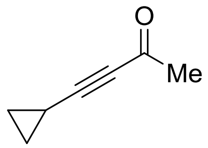

30: C_7H_8O
MW: 108.14

Synthesis according to general procedure A: (cyclopropylethynyl)trimethylsilane (138 mg, 1.00 mmol, 1.00 equiv), anhydride acetic (**2**) (940 µL, 1.02 g, 10.0 mmol, 10.0 equiv), $FeCl_3$ (16.2 mg, 0.10 mmol, 0.10 equiv), 30 min. After 30 min, the reaction was cooled to 0 °C using an ice bath. The reaction was quenched with a NaOH (2.0 M, 12 mL). The aqueous phase was extracted with Et_2O (3 × 5 mL). The combined organic phases were washed with sat. aqueous sol. $NaHCO_3$ (10 mL) and once more with water. The organic phase was concentrated (50 °C, 1 atm). The product was obtained after purification by column chromatography (pentane/Et_2O : 9/1) to yield **30** as a yellowish liquid (41.0 mg, 0.38 mmol, 38% yield).

R*f* = 0.33 (pentane/ether : 9/1); ^{1}**H NMR** (300 MHz, $CDCl_3$) δ: 2.29 (s, 3H), 1.44 – 1.34 (m, 1H), 1.02 – 0.86 (m, 4H); ^{13}**C NMR** (75 MHz, $CDCl_3$) δ: 184.7, 98.6, 77.3, 32.6, 9.8, -0.3.

Spectral data were consistent with those previously reported.[27]

5-oxohex-3-yn-1-yl acetate (31)

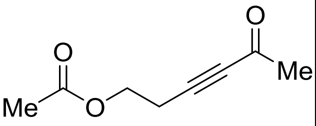

31: $C_8H_{10}O_3$
MW: 154.17

Synthesis according to general procedure 1: 4-(trimethylsilyl)but-3-yn-1-ol (142 mg, 1.00 mmol, 1.00 equiv), anhydride acetic (**2**) (940 µL, 1.02 g, 10.0 mmol, 10.0 equiv), $FeCl_3$ (16.2 mg, 0.10 mmol, 0.10 equiv), 16 hours. The product was obtained after purification by column chromatography (pentane/Et_2O : 3/2) to yield **31** as a yellow liquid (72 mg, 0.47 mmol, 47% yield).

R*f* = 0.30 (pentane/ether : 3/2); **^{1}H NMR** (300 MHz, $CDCl_3$) δ: 4.21 (t, *J* = 6.7 Hz, 2H), 2.71 (t, *J* = 6.7 Hz, 2H), 2.33 (s, 3H), 2.08 (s, 3H); **^{13}C NMR** (75 MHz, $CDCl_3$) δ: 184.3, 170.6, 88.7, 82.1, 61.1, 32.6, 20.7, 19.4. **HRMS** (ASAP) m/z calcd for C_8 H_{11} O_3, $[M + H]^+$ 155.0703, found 155.0703.

(2-((trimethylsilyl)ethynyl)phenyl)methylene diacetate (37)

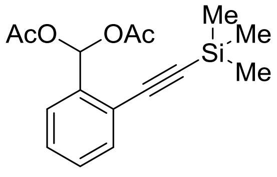

37: $C_{16}H_{20}O_4Si$
MW: 304.42

Synthesis according to general procedure A: 2-((trimethylsilyl)ethynyl)benzaldehyde (202 mg, 1.00 mmol, 1.00 equiv), anhydride acetic (**2**) (940 µL, 1.02 g, 10.0 mmol, 10.0 equiv), $FeCl_3$ (16.2 mg, 0.10 mmol, 0.10 equiv), 30 min. The product was obtained after purification by column chromatography (pentane/Et_2O : 9/1) to yield **37** as a white solid (160 mg, 1.94 mmol, 53% yield).

R*f* = 0.28 (pentane/ether : 9/1); **^{1}H NMR** (300 MHz, $CDCl_3$) δ: 8.03 (s, 1H), 7.52 – 7.48 (m, 2H), 7.39 – 7.31 (m, 2H), 2.13 (s, 6H), 0.25 (s, 9H); **^{13}C NMR** (75 MHz, $CDCl_3$) δ: 168.4, 137.6, 132.7, 129.3, 128.7, 125.7, 122.3, 101.0, 100.7, 88.2, 20.7, -0.2. **HRMS** (ASAP) m/z calcd for C_{16} H_{20} O_4 Na Si, $[M + Na]^+$ 327.1023, found 327.1026; **m. p.** 47-48 °C.

3-oxo-1-phenylbutyl acetate (42)

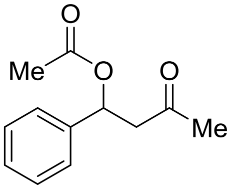

42: $C_{12}H_{14}O_3$
MW: 206.24

Synthesis according to general procedure A: (*E*)-trimethyl(styryl)silane (176 mg, 1.00 mmol, 1.00 equiv), anhydride acetic (**2**) (940 µL, 1.02 g, 10.0 mmol, 10.0 equiv), $FeCl_3$ (16.2 mg, 0.10 mmol, 0.10 equiv), 2 hours. The product was obtained after purification by column chromatography (pentane/Et_2O : 7/3) to yield **42** as a yellow liquid (20.6 mg, 0.10 mmol, 10% yield).

R$_f$ = 0.26 (pentane/ether : 7/3); **^{1}H NMR** (300 MHz, $CDCl_3$) δ: 7.43 – 7.31 (m, 5H), 6.24 (dd, *J* = 8.7, 5.0 Hz, 1H), 3.17 (dd, *J* = 16.6, 8.7 Hz, 1H), 2.88 (dd, *J* = 16.6, 5.0 Hz, 1H), 2.21 (s, 3H), 2.09 (s, 3H); **^{13}C NMR** (75 MHz, $CDCl_3$) δ: 204.8, 170.0, 139.8, 128.8, 128.4, 126.6, 71.7, 50.0, 30.6, 21.2.

Spectral data were consistent with those previously reported.[28]

1-phenyloct-1-yn-3-one (43)

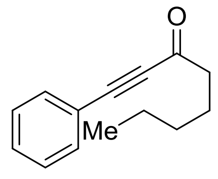

43: $C_{14}H_{16}O$
MW: 200.28

Synthesis according to general procedure A: trimethyl(phenylethynyl)silane (**1**) (197 µL, 174 mg, 1.00 mmol, 1.00 equiv), hexanoic anhydride (2.30 mL, 2.14 g, 10.0 mmol, 10.0 equiv), $FeCl_3$ (16.2 mg, 0.10 mmol, 0.10 equiv), 30 min. After 30 min, the reaction was cooled to 0 °C using an ice bath. The reaction was quenched with a NaOH (2.0 M, 15 mL) and stirred overnight. The aqueous phase was extracted with Et_2O (3 × 5 mL). The combined organic phases were washed water (2 × 10 mL). The organic phase was concentrated. The product was obtained after purification by column chromatography (pentane/Et_2O : 19/1) to yield **43** as a yellow liquid (129 mg, 0.64 mmol, 64% yield).

R$_f$ = 0.32 (pentane/ether : 19/1); **^{1}H NMR** (300 MHz, $CDCl_3$) δ: 7.61 – 7.53 (m, 2H), 7.48 – 7.42 (m, 1H), 7.41 – 7.35 (m, 2H), 2.65 (td, *J* = 7.5, 1.1 Hz, 2H), 1.81 – 1.67 (m, 2H), 1.42 – 1.30 (m, 4H), 0.94 – 0.89 (m, 3H); **^{13}C NMR** (75 MHz, $CDCl_3$) δ: 188.4, 133.1, 130.8, 128.7, 120.2, 90.7, 88.0, 45.6, 31.3, 24.0, 22.5, 14.0.

Spectral data were consistent with those previously reported.[29]

4-methyl-1-phenylpent-1-yn-3-one (44)

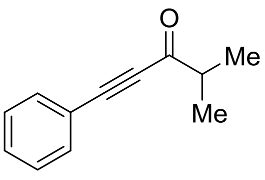

44: $C_{12}H_{12}O$
M.W.: 172.23

Synthesis according to general procedure A: trimethyl(phenylethynyl)silane (**1**) (197 µL, 174 mg, 1.00 mmol, 1.00 equiv), isobutyric anhydride (1.66 mL, 1.58 g, 10.0 mmol, 10.0 equiv), $FeCl_3$ (16.2 mg, 0.10 mmol, 0.10 equiv), 30 min. After 30 min, the reaction was cooled to 0 °C using an ice bath. The reaction was quenched with a NaOH (2.0 M, 15 mL) and stirred overnight. The aqueous phase was extracted with Et_2O (3 × 5 mL). The combined organic phases were washed with water (2 × 10 mL). The organic phase was concentrated. The product was obtained after

purification by column chromatography (pentane/Et_2O : 19/1) to yield **44** as a yellow liquid (89.5 mg, 0.52 mmol, 52% yield).

R_f = 0.32 (pentane/ether : 19/1); **^{1}H NMR** (300 MHz, $CDCl_3$) δ: 7.61 – 7.56 (m, 2H), 7.49 – 7.35 (m, 3H), 2.76 (hept, *J* = 6.9 Hz, 1H), 1.27 (d, J = 7.0 Hz, 6H); **^{13}C NMR** (75 MHz, $CDCl_3$) δ: 192.3, 133.1, 130.7, 128.7, 120.2, 91.7, 86.9, 43.2, 18.1.

Spectral data were consistent with those previously reported.[30]

■ Computational methods

All computations were performed with the ORCA program package (version 5.0).[31] Geometries were optimized using the r2SCAN-3c method.[32] Frequency calculations at the same level of theory confirmed the absence of imaginary frequencies for minima and the presence of a single imaginary frequency for transition states, and provided the thermochemical corrections. Final single-point energy calculations were carried out with the dispersion-corrected ωB97M-V functional in combination with the def2-QZVPP basis set.[33] The reaction solvent, acetic anhydride, was modeled using the CPCM model with parameters for acetone.[34] For all DFT calculations, the DefGrid3 option was applied.

Dissociation of acylium ion **57**

To assess the kinetic barrier for dissociation of acylium ion **57** from the $Ac_2O–FeCl_3$ complex **52**, we performed a relaxed potential energy surface scan of the C1–O17 bond length from 1.36 Å to 3.00 Å in 30 steps. However, at the end of this scan, the acylium ion was not released. Instead, it coordinated to oxygen O8.

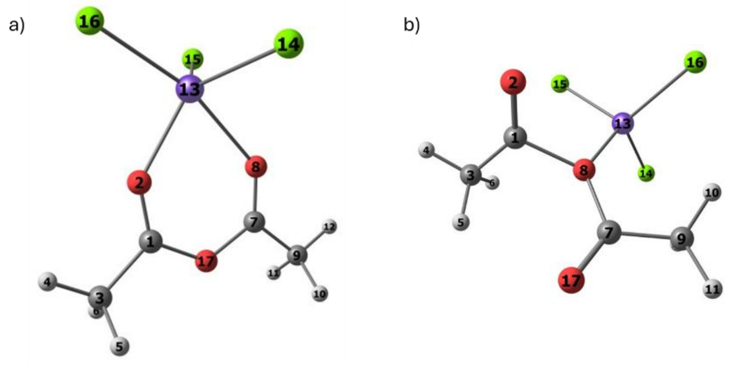

Figure S3. The structure of complex **53** (a) and the structure that was obtained at the end of a potential energy surface scan of the C1–O17 bond length (b).

Cartesian coordinates and energies

Table S4. Single point energies and relevant thermochemical contributions in Hartree.

	E wB97M-V/def2-QZVPP	G-E r2SCAN-3c
**52**	-3026.266349	0.05715061
**TS$_{52/53}$**	-3026.263315	0.05756134
**53**	-3026.263573	0.05683513
**1**	-717.0809527	0.16971153
**TS$_{53 + 1/54 + 55}$**	-3743.318277	0.25186175
**55**	-870.1532379	0.21347948
**54**	-2873.179687	0.0128739
**3**	-461.0726339	0.11027304
**56**	-637.8248822	0.12386307
**2**	-381.7916322	0.06427809
**57**	-153.0442912	0.02004233

Coordinates

52

C	-0.041537000	0.289393000	-1.816377000
O	-0.037286000	0.068527000	-3.020711000
C	-0.056811000	-0.768215000	-0.784322000
H	-0.079267000	-1.749362000	-1.255422000
H	-0.929903000	-0.623322000	-0.138491000
H	0.832920000	-0.658840000	-0.153783000
C	0.015898000	2.702674000	-2.005088000
O	0.051281000	2.721289000	-3.214887000
C	0.018157000	3.880861000	-1.113131000
H	-0.891127000	3.865495000	-0.501679000
H	0.870929000	3.812620000	-0.428877000
H	0.068417000	4.792388000	-1.706602000
Fe	-0.023477000	1.127902000	-4.814261000
Cl	-1.879078000	2.132396000	-5.513306000
Cl	1.873974000	2.003146000	-5.568473000
Cl	-0.106656000	-0.821706000	-5.932256000
O	-0.032256000	1.530076000	-1.261669000

TS$_{52/53}$

C	0.010664000	0.159863000	-1.767607000
O	0.628170000	-0.110798000	-2.757415000
C	-0.716645000	-0.740458000	-0.848225000
H	-0.581906000	-1.773412000	-1.166169000
H	-1.779457000	-0.470755000	-0.866868000
H	-0.361523000	-0.594748000	0.176604000

C	0.321882000	2.559624000	-2.004689000
O	0.416730000	2.570292000	-3.233521000
C	0.616622000	3.742928000	-1.168615000
H	-0.220856000	3.924769000	-0.487626000
H	1.492727000	3.516844000	-0.548569000
H	0.808455000	4.611303000	-1.796834000
Fe	-0.050289000	1.479189000	-4.860473000
Cl	-1.937410000	0.365203000	-4.604217000
Cl	-0.518909000	3.180734000	-6.203573000
Cl	1.605950000	0.386183000	-5.803522000
O	-0.080026000	1.498562000	-1.288017000

53

C	0.069390000	0.119335000	-1.692231000
O	0.934525000	-0.191247000	-2.450830000
C	-0.900877000	-0.718528000	-0.955008000
H	-0.793230000	-1.757495000	-1.264244000
H	-1.915669000	-0.356213000	-1.147450000
H	-0.714599000	-0.617998000	0.120066000
C	0.354461000	2.519914000	-2.012770000
O	0.494445000	2.481746000	-3.240564000
C	0.647232000	3.731201000	-1.216842000
H	-0.171555000	3.917336000	-0.515642000
H	1.545900000	3.532954000	-0.618853000
H	0.812035000	4.586021000	-1.870992000
Fe	-0.094622000	1.524430000	-4.883978000
Cl	-1.859086000	0.307914000	-4.384838000
Cl	-0.682198000	3.181564000	-6.207221000
Cl	1.564193000	0.400748000	-5.766276000
O	-0.094602000	1.495195000	-1.287481000

1

C	-3.182899000	2.046514000	0.212407000
C	-2.941226000	2.406753000	1.353463000
C	-2.656296000	2.823816000	2.680817000
C	-2.835038000	1.937737000	3.758361000
C	-2.189358000	4.126385000	2.932698000
C	-2.549749000	2.349462000	5.053695000
C	-1.907702000	4.526316000	4.232471000
C	-2.085936000	3.641920000	5.295570000
H	-3.195834000	0.931848000	3.566169000
H	-2.051192000	4.812273000	2.102371000
H	-2.690086000	1.657704000	5.879457000
H	-1.546727000	5.534157000	4.417040000
H	-1.863542000	3.959140000	6.310347000

Si	-3.541833000	1.498606000	-1.512324000
C	-5.401177000	1.526961000	-1.762419000
H	-5.648431000	1.205779000	-2.781403000
H	-5.902217000	0.851499000	-1.060564000
H	-5.803117000	2.535454000	-1.615214000
C	-2.871717000	-0.240614000	-1.724142000
H	-1.787903000	-0.266718000	-1.566627000
H	-3.339216000	-0.931307000	-1.013745000
H	-3.078178000	-0.602231000	-2.738575000
C	-2.694609000	2.687881000	-2.689905000
H	-1.610168000	2.690344000	-2.534184000
H	-2.889707000	2.392758000	-3.727830000
H	-3.063887000	3.709777000	-2.550724000

TS$_{53 + 1/54 + 55}$

C	-0.445347000	1.023091000	-0.081005000
O	-0.048986000	1.243909000	-1.176040000
C	-0.491076000	-0.244884000	0.702398000
H	-1.162563000	-0.934541000	0.184353000
H	-0.839703000	-0.076009000	1.719149000
H	0.522770000	-0.659789000	0.708380000
C	0.540574000	3.312583000	0.970765000
O	0.013033000	3.897611000	-0.032274000
C	1.365057000	4.082373000	1.952377000
H	0.793814000	4.943984000	2.310563000
H	1.665418000	3.449575000	2.787174000
H	2.251061000	4.468719000	1.436704000
Fe	-0.185307000	5.610681000	-0.864226000
Cl	-2.323879000	6.154358000	-0.660303000
Cl	1.079513000	7.162990000	0.066590000
Cl	0.382723000	5.334572000	-2.982981000
O	0.382935000	2.072705000	1.171845000
C	-2.318507000	1.717278000	0.128262000
C	-2.468979000	2.198706000	1.261462000
C	-2.507812000	2.714289000	2.561650000
C	-2.757595000	1.855243000	3.653838000
C	-2.277053000	4.089009000	2.784748000
C	-2.760459000	2.367167000	4.940433000
C	-2.286330000	4.584077000	4.078112000
C	-2.523856000	3.726658000	5.153708000
H	-2.938260000	0.800724000	3.470583000
H	-2.101336000	4.744747000	1.937412000
H	-2.947392000	1.708898000	5.783359000
H	-2.108269000	5.640788000	4.252677000
H	-2.527553000	4.121019000	6.165852000

Si	-3.276787000	1.430323000	-1.472779000
C	-5.072628000	1.758880000	-1.059637000
H	-5.692711000	1.624899000	-1.954061000
H	-5.431689000	1.070626000	-0.287010000
H	-5.206769000	2.784801000	-0.699564000
C	-3.021265000	-0.342019000	-2.016082000
H	-1.973495000	-0.548526000	-2.257242000
H	-3.350197000	-1.047627000	-1.245353000
H	-3.617807000	-0.525977000	-2.917870000
C	-2.632484000	2.647620000	-2.736471000
H	-1.563956000	2.508537000	-2.923643000
H	-3.170187000	2.503888000	-3.681879000
H	-2.794368000	3.678186000	-2.403298000

55

C	-0.418737000	0.843281000	-0.355449000
O	0.212449000	1.389033000	-1.220978000
C	-0.053170000	-0.420012000	0.346451000
H	-0.884990000	-1.130364000	0.280663000
H	0.086910000	-0.192995000	1.410816000
H	0.857579000	-0.840455000	-0.082772000
C	-1.759213000	1.453657000	0.095423000
C	-2.027917000	1.966492000	1.214068000
C	-2.391917000	2.562938000	2.397711000
C	-2.962270000	1.776546000	3.434317000
C	-2.203884000	3.960407000	2.570883000
C	-3.333742000	2.387566000	4.613640000
C	-2.584445000	4.546924000	3.759623000
C	-3.145661000	3.764529000	4.774693000
H	-3.097384000	0.710959000	3.279741000
H	-1.767147000	4.541425000	1.765155000
H	-3.771972000	1.802083000	5.415517000
H	-2.449445000	5.613320000	3.908036000
H	-3.442858000	4.235981000	5.707312000
Si	-3.208340000	1.470087000	-1.241297000
C	-4.752229000	2.021608000	-0.359840000
H	-5.587404000	2.017984000	-1.070516000
H	-5.005958000	1.347988000	0.465759000
H	-4.647727000	3.038423000	0.034428000
C	-3.285321000	-0.296288000	-1.833324000
H	-2.339294000	-0.611714000	-2.286179000
H	-3.532158000	-0.981009000	-1.015479000
H	-4.070541000	-0.375727000	-2.595361000
C	-2.630196000	2.673745000	-2.539961000
H	-1.686492000	2.358758000	-2.994336000
H	-3.394450000	2.721169000	-3.326801000
H	-2.505208000	3.677262000	-2.120313000

54

C	-0.423128000	2.521090000	-2.150900000
O	-0.188331000	3.034312000	-3.310427000
C	-0.617983000	3.420870000	-0.975220000
H	-1.475321000	4.075405000	-1.163240000
H	-0.782659000	2.842578000	-0.065657000
H	0.262351000	4.061256000	-0.860877000
Fe	-0.056880000	1.232857000	-4.261827000
Cl	0.342134000	2.215655000	-6.280640000
Cl	1.769756000	-0.038636000	-3.968610000
Cl	-1.915780000	0.040983000	-4.669273000
O	-0.478062000	1.265644000	-2.065132000

3

C	-0.184077000	0.748147000	-0.287105000
O	0.322699000	1.381408000	-1.210212000
C	-0.115079000	-0.749752000	-0.183408000
H	-1.125876000	-1.164866000	-0.103715000
H	0.417006000	-1.027687000	0.734044000
H	0.397989000	-1.163570000	-1.053522000
C	-0.881158000	1.422088000	0.771505000
C	-1.473726000	1.984206000	1.672397000
C	-2.161506000	2.649191000	2.712814000
C	-2.754658000	1.910571000	3.753920000
C	-2.258853000	4.053497000	2.712370000
C	-3.430476000	2.569502000	4.770714000
C	-2.937538000	4.699236000	3.735706000
C	-3.523656000	3.961139000	4.763977000
H	-2.676550000	0.827744000	3.750616000
H	-1.799563000	4.619225000	1.907664000
H	-3.887471000	1.996767000	5.572305000
H	-3.010944000	5.782710000	3.732518000
H	-4.054377000	4.471759000	5.562263000

56

C	-0.738480000	0.385232000	-0.457928000
O	-0.347390000	0.242648000	-1.604039000
C	0.079838000	0.085515000	0.763326000
H	-0.440416000	-0.653511000	1.380987000
H	0.182800000	0.994505000	1.364902000
H	1.063546000	-0.288217000	0.479615000
Si	-3.129578000	1.267838000	-1.356037000

C	-4.576264000	1.797980000	-0.305644000
H	-5.412422000	2.108884000	-0.942473000
H	-4.922991000	0.976858000	0.331669000
H	-4.307729000	2.643477000	0.337482000
C	-3.547474000	-0.233374000	-2.382867000
H	-2.715512000	-0.523045000	-3.031451000
H	-3.803136000	-1.085085000	-1.742288000
H	-4.417031000	-0.016401000	-3.014952000
C	-2.469696000	2.686939000	-2.372590000
H	-1.648459000	2.370566000	-3.022350000
H	-3.270339000	3.092583000	-3.002737000
H	-2.110667000	3.495329000	-1.725453000
O	-1.964881000	0.834873000	-0.158618000

2

C	-0.260764000	0.298538000	-1.791130000
O	-0.708750000	0.179814000	-2.899554000
C	0.025655000	-0.793357000	-0.815511000
H	-0.046637000	-1.760280000	-1.313069000
H	-0.709437000	-0.742759000	-0.003971000
H	1.014528000	-0.656051000	-0.369383000
C	0.232992000	2.681790000	-1.962384000
O	0.674718000	2.642131000	-3.078885000
C	-0.055306000	3.900323000	-1.151499000
H	-1.075446000	3.856204000	-0.758417000
H	0.623751000	3.927255000	-0.292145000
H	0.085267000	4.791857000	-1.762158000
O	-0.011156000	1.538119000	-1.202935000

57

C	-0.308352000	-0.732098000	-1.719209000
O	-1.228559000	-0.532216000	-2.326228000
C	0.842460000	-0.985363000	-0.958234000
H	1.112913000	-2.043406000	-1.091158000
H	0.607091000	-0.771516000	0.095036000
H	1.644641000	-0.325914000	-1.321375000

References

[1] P. Charki, M. Cordier, K. E. O. Ylijoki, D. S. Müller, *Chem. Eur. J.* **2025**, *31*, e202403979.
[2] M. Cordier, D. S. Müller, M. Devillard, *Organometallics* **2024**, *43*, 929–933.
[3] J. P. Brand, C. Chevalley, R. Scopelliti, J. Waser, *Chemistry A European J* **2012**, *18*, 5655–5666.
[4] Y. Huang, X. Zhang, X. Dong, X. Zhang, *Adv Synth Catal* **2020**, *362*, 782–788.
[5] J. M. Feilner, I. Plangger, K. Wurst, T. Magauer, *Chemistry A European J* **2021**, *27*, 12410–12421.
[6] Y. Liu, M. Cheng, Z. Zhang, Y. Zhong, *Preparation of N-Arylsulfonamide-N-β-D-Glucopyranosyl Diamide Compound as Antitumor Drug*, **2021**, CN113024617A.
[7] A. Boelke, L. D. Caspers, B. J. Nachtsheim, *Org. Lett.* **2017**, *19*, 5344–5347.
[8] E. C. Vatansever, K. Kılıç, M. S. Özer, G. Koza, N. Menges, M. Balci, *Tetrahedron Letters* **2015**, *56*, 5386–5389.
[9] Y. Hu, Z. Zhang, Y. Liu, W. Zhang, *Angewandte Chemie International Edition* **2021**, *60*, 16989–16993.
[10] Y.-M. Zhang, M.-L. Yuan, W.-P. Liu, J.-H. Xie, Q.-L. Zhou, *Org. Lett.* **2018**, *20*, 4486–4489.
[11] B. R. Kim, H.-G. Lee, S.-B. Kang, K.-J. Jung, G. H. Sung, J.-J. Kim, S.-G. Lee, Y.-J. Yoon, *Tetrahedron* **2013**, *69*, 10331–10336.
[12] C.-S. Li, C. K. Lau, M. Therien, P. Prasit, *PYRONES AS INHIBITORS OF CYCLOOXYGENASE-2*, **2002**, WO2002002547 A1.
[13] J. Zhang, Z. Miao, *Org. Biomol. Chem.* **2018**, *16*, 9461–9471.
[14] B. M. Trost, E. Gnanamani, *Org. Lett.* **2020**, *22*, 1675–1680.
[15] C.-N. Lo, C.-S. Hsu, *Journal of Polymer Science Part A: Polymer Chemistry* **2011**, *49*, 3355–3365.
[16] B. Sandmann, B. Happ, J. Vitz, R. M. Paulus, M. D. Hager, P. Burtscher, N. Moszner, U. S. Schubert, *Macro Chemistry & Physics* **2014**, *215*, 1603–1608.
[17] M. S. B. Wills, R. L. Danheiser, *J. Am. Chem. Soc.* **1998**, *120*, 9378–9379.
[18] S. Mukherjee, D. Kontokosta, A. Patil, S. Rallapalli, D. Lee, *J. Org. Chem.* **2009**, *74*, 9206–9209.
[19] J. P. Hopewell, J. E. D. Martins, T. C. Johnson, J. Godfrey, M. Wills, *Org. Biomol. Chem.* **2012**, *10*, 134–145.
[20] G. A. Brito, W. Jung, M. Yoo, M. J. Krische, *Angew Chem Int Ed* **2019**, *58*, 18803–18807.
[21] C. R. Kumar, C. Tsai, Y. Chao, J. Lee, *Chemistry A European J* **2011**, *17*, 8696–8703.
[22] A. B. Smith, Iii, P. A. Levenberg, J. Z. Suits, *Synthesis* **1986**, *1986*, 184–189.
[23] A. Shatskiy, T. Kivijärvi, H. Lundberg, F. Tinnis, H. Adolfsson, *ChemCatChem* **2015**, *7*, 3818–3821.
[24] Y. Xing, G. A. O'Doherty, *Org. Lett.* **2009**, *11*, 1107–1110.
[25] E. Piers, R. D. Tillyer, *Can. J. Chem.* **1996**, *74*, 2048–2063.
[26] S. S. Chandankar, S. Raghavan, *J. Org. Chem.* **2019**, *84*, 9584–9602.
[27] K. Liu, G. Wang, Z.-W. Zhang, Y.-Y. Shi, Z.-S. Ye, *Org. Lett.* **2022**, *24*, 6489–6493.
[28] H. Kohls, M. Anderson, J. Dickerhoff, K. Weisz, A. Córdova, P. Berglund, H. Brundiek, U. T. Bornscheuer, M. Höhne, *Adv Synth Catal* **2015**, *357*, 1808–1814.
[29] L. Anastasia, E. Negishi, *Org. Lett.* **2001**, *3*, 3111–3113.
[30] S. S. Chandankar, S. Raghavan, *J. Org. Chem.* **2019**, *84*, 9584–9602.

[31] a) F. Neese, *WIREs Comput Mol Sci* **2022**, *12*; b) F. Neese, *WIREs Comput Mol Sci* **2012**, *2*, 73
[32] S. Grimme, A. Hansen, S. Ehlert, J.-M. Mewes, *J. Chem. Phys.* **2021**, *154*, 64103.
[33] a) N. Mardirossian, M. Head-Gordon, *J. Chem. Phys.* **2016**, *144*, 214110; b) F. Weigend, R. Ahlrichs, *Phys. Chem. Chem. Phys.* **2005**, *7*, 3297.
[34] V. Barone, M. Cossi, *J. Phys. Chem. A* **1998**, *102*, 1995

■ NMR Spectra

The ^{1}H NMR (300 MHz) and $^{13}C\{^{1}H\}$ NMR (76 MHz) spectrum for **3** (using $CDCl_3$ as solvent)

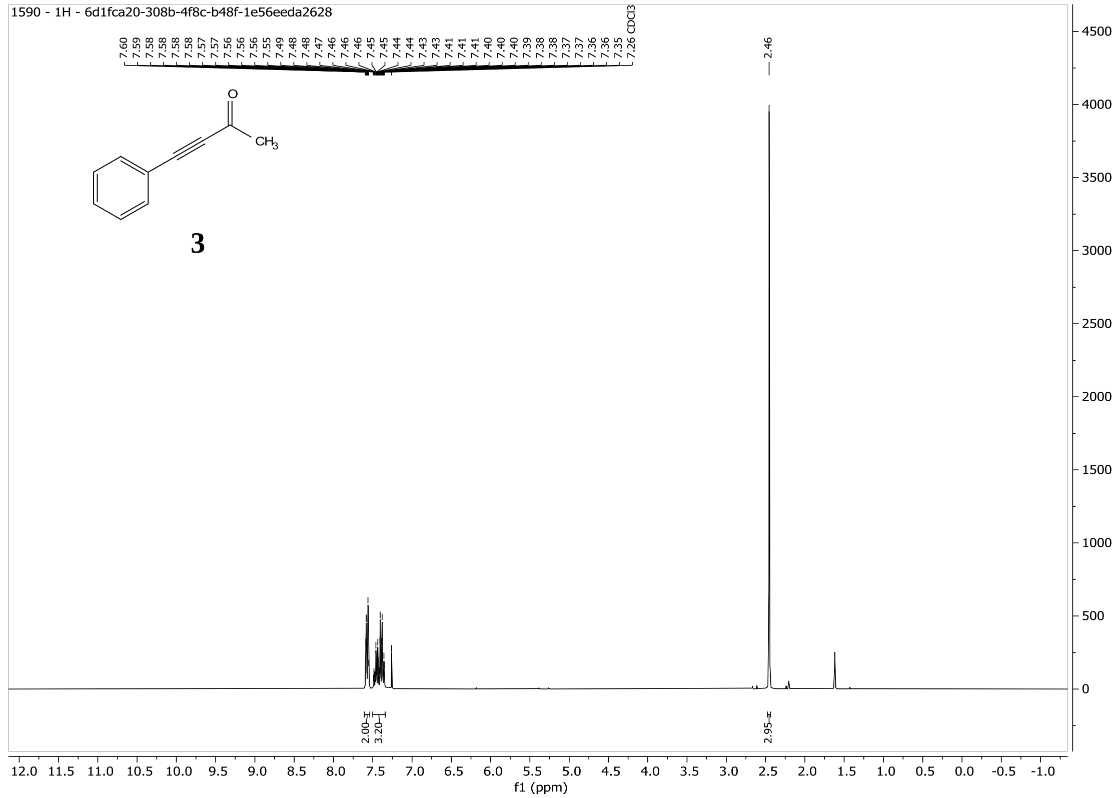

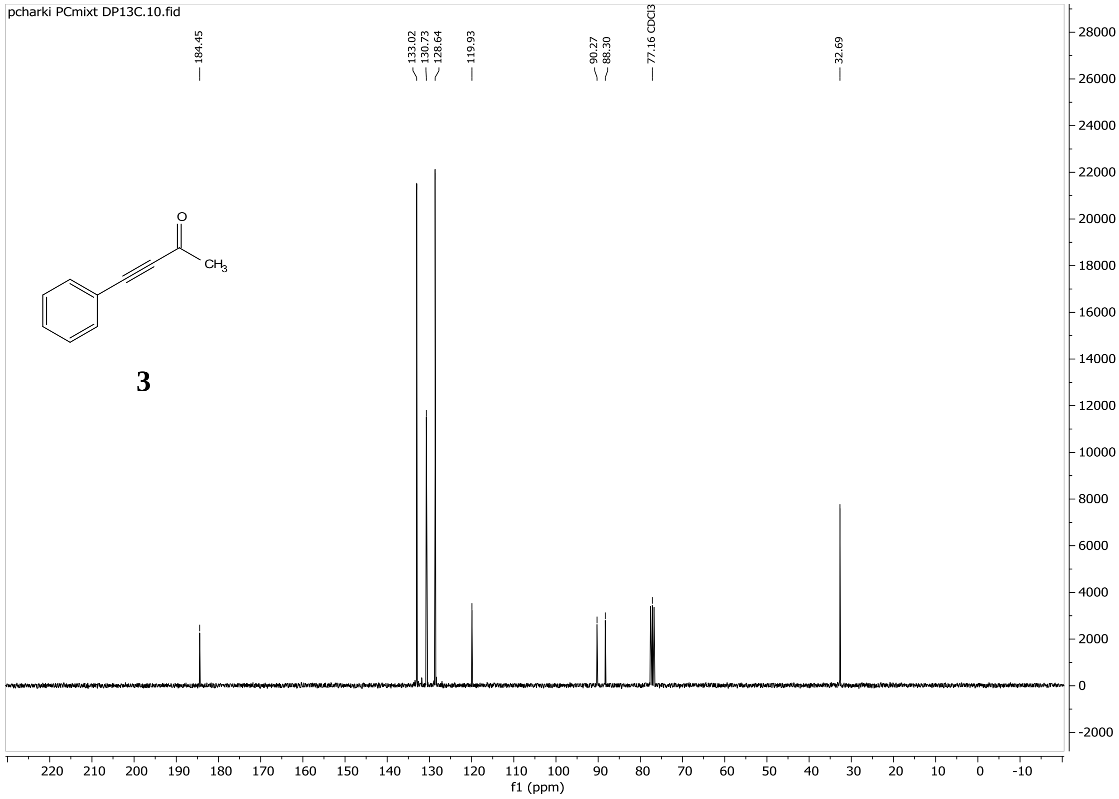

The ^{1}H NMR (300 MHz) and $^{13}C\{^{1}H\}$ NMR (76 MHz) spectrum for **5** (using $CDCl_3$ as solvent)

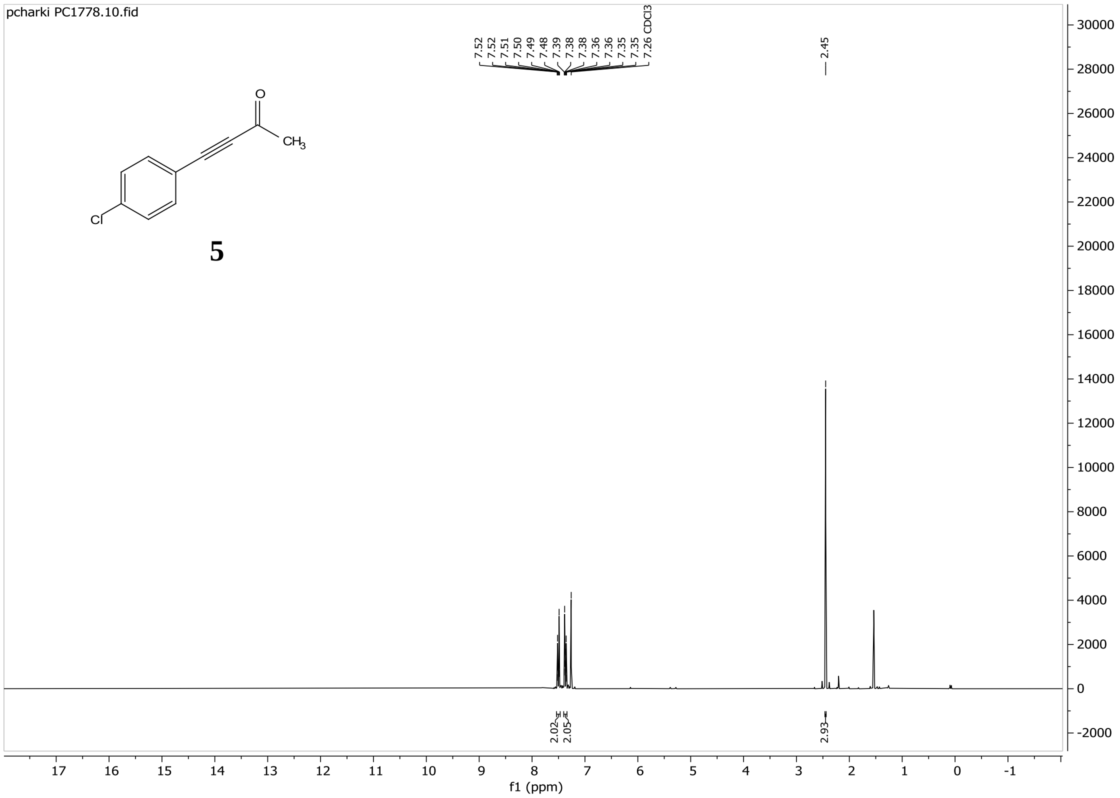

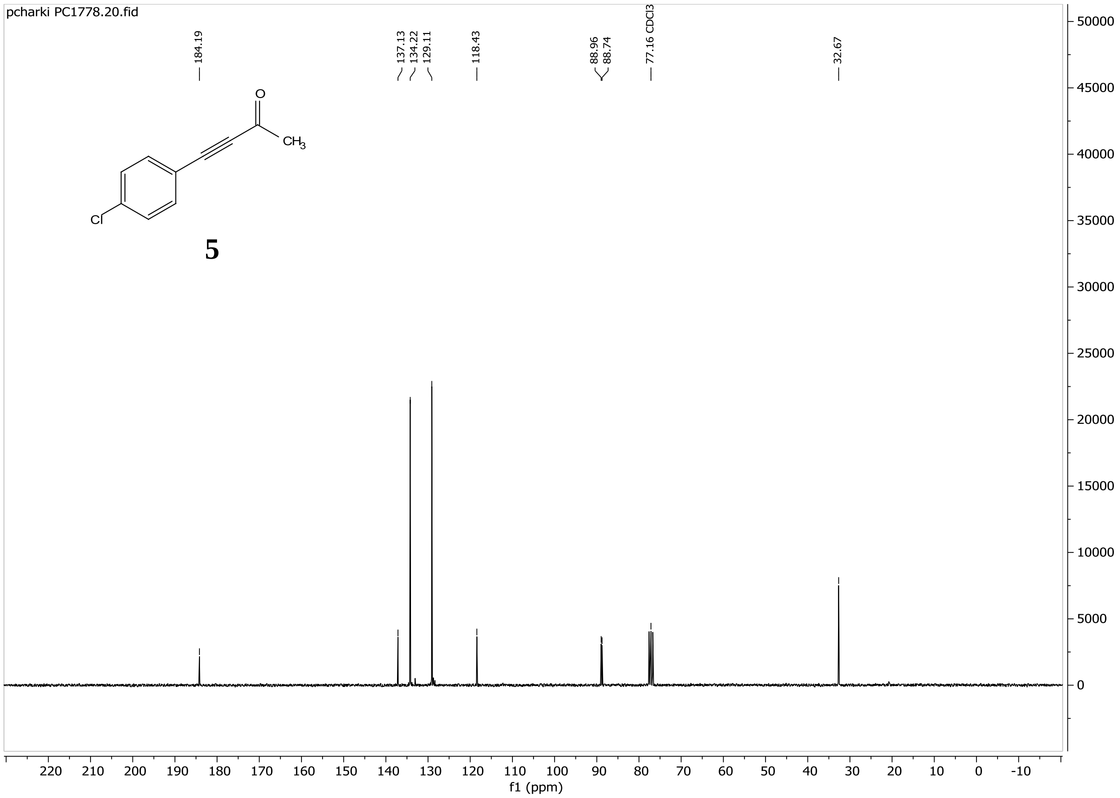

The ^{1}H NMR (300 MHz) and $^{13}C\{^{1}H\}$ NMR (76 MHz) spectrum for **6** (using $CDCl_3$ as solvent)

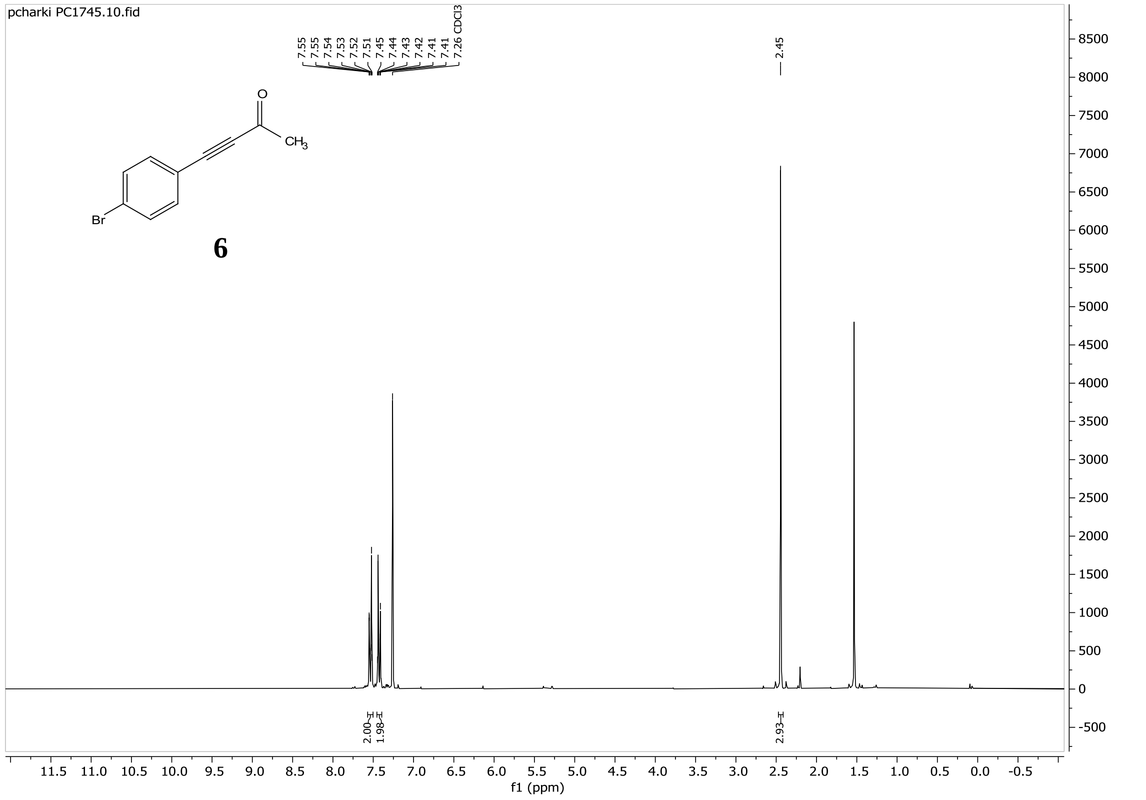

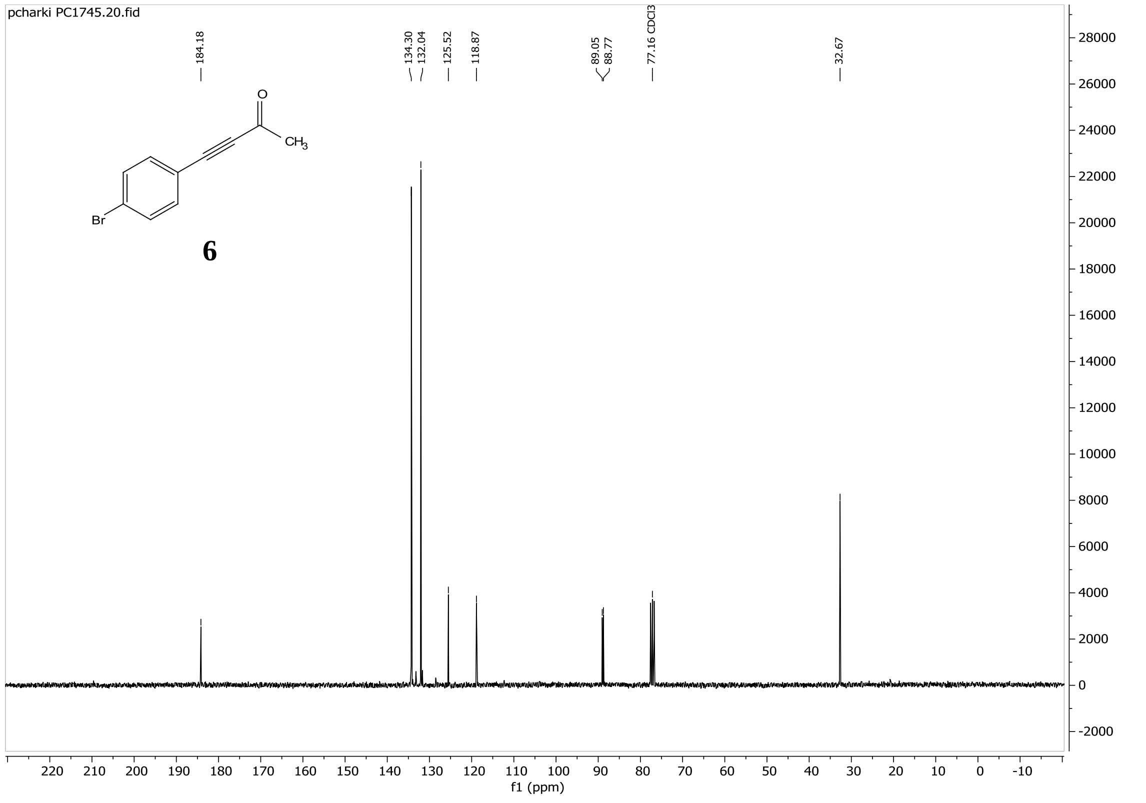

The ^{1}H NMR (300 MHz), $^{13}C\{^{1}H\}$ NMR (76 MHz) and ^{19}F NMR (282 MHz) spectrum for **7** (using $CDCl_3$ as solvent)

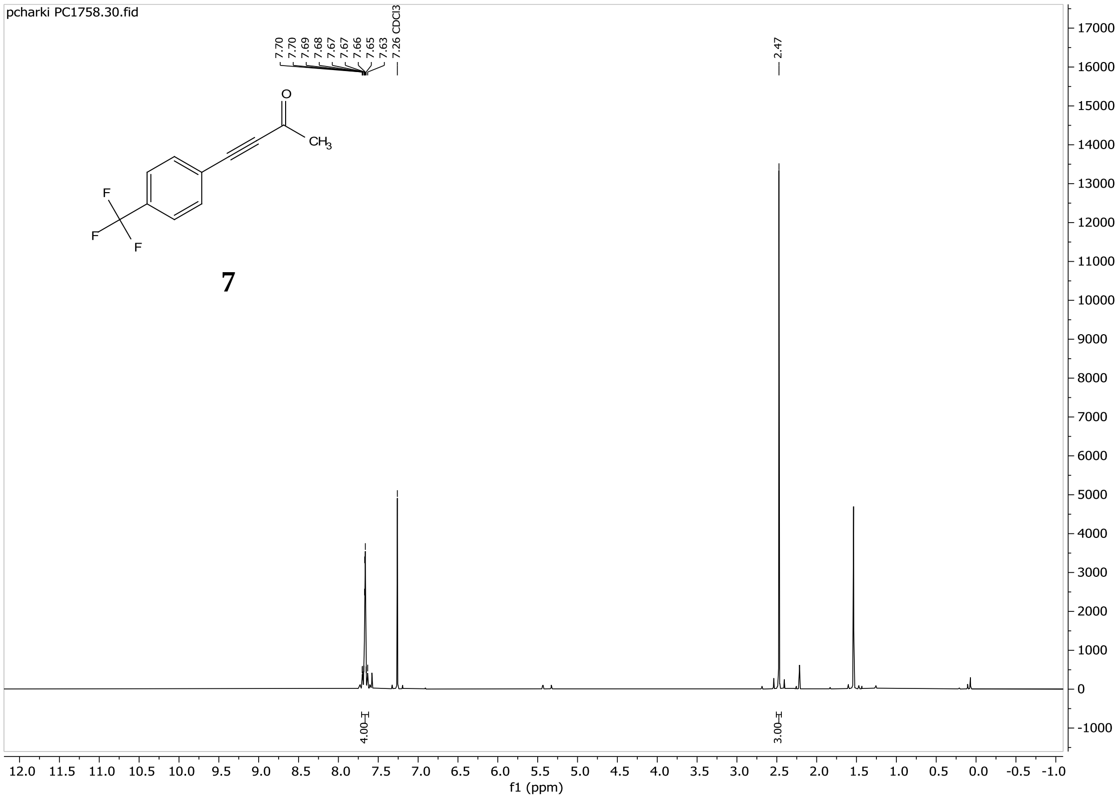

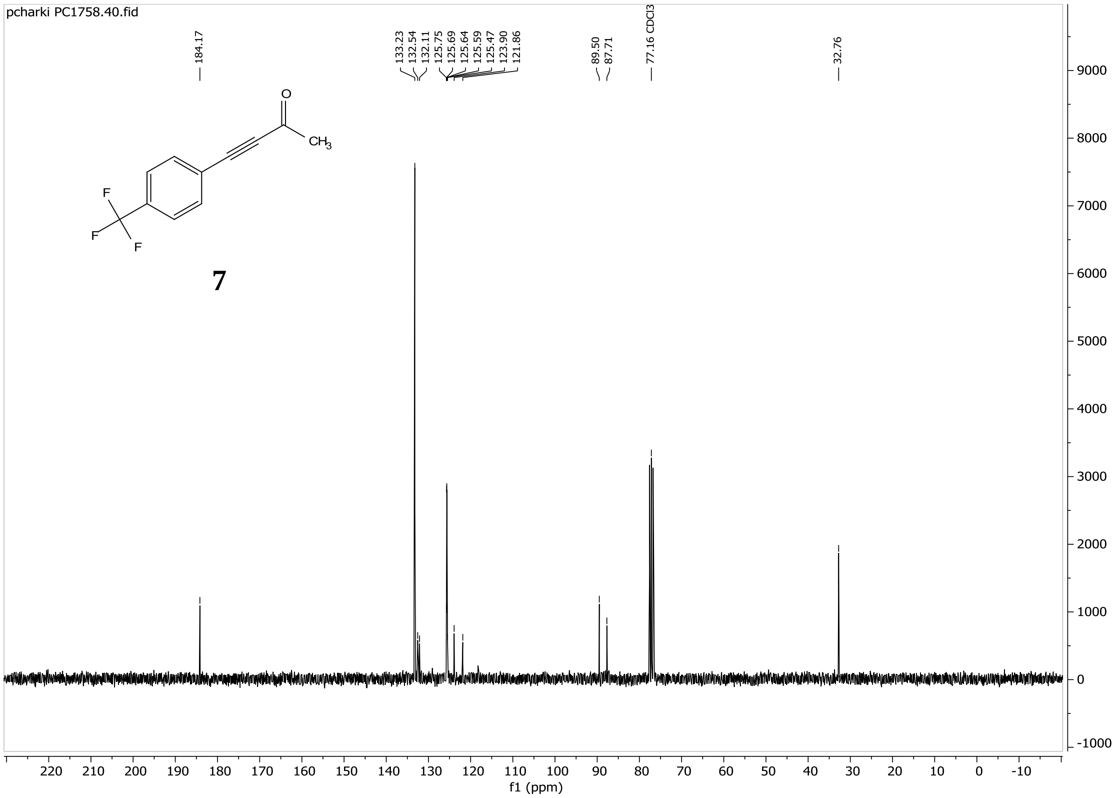

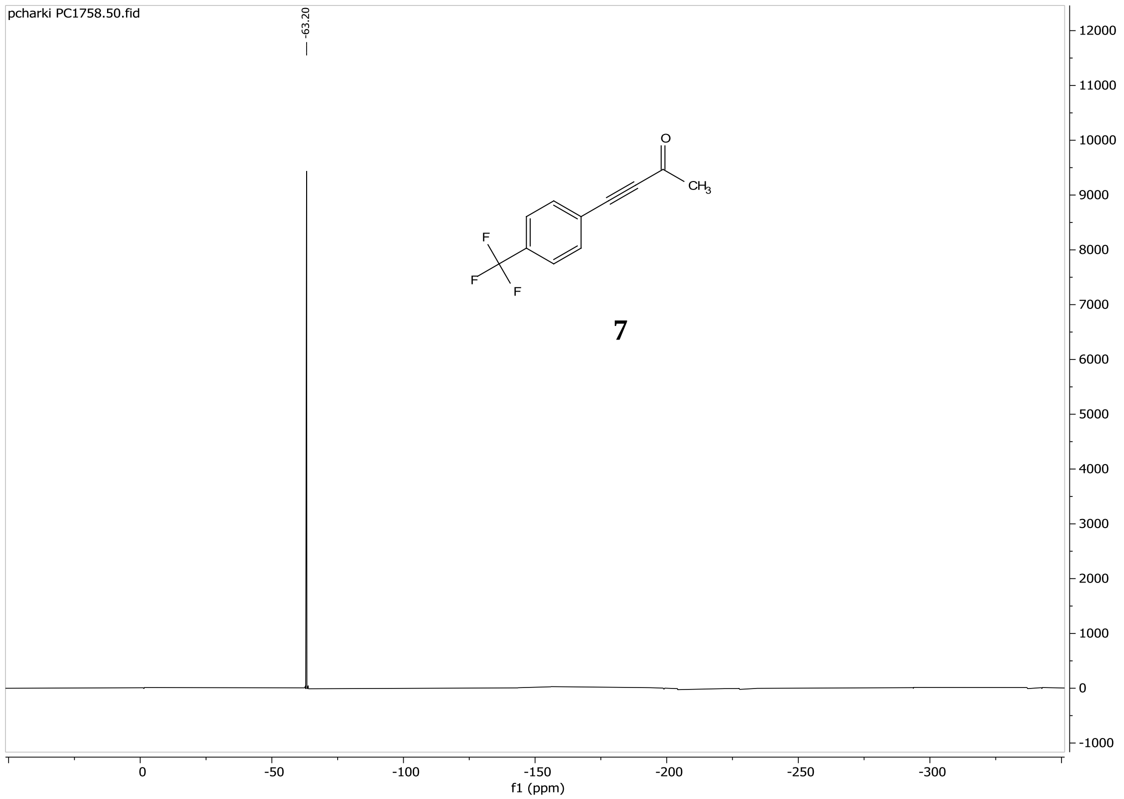
pcharki PC1758.50.fid
-63.20
O
CH3
F
F
F
7
12000
11000
10000
9000
8000
7000
6000
5000
4000
3000
2000
1000
0
-1000
0
-50
-100
-150
-200
-250
-300
f1 (ppm)

The ^{1}H NMR (300 MHz) and ^{13}C{^{1}H} NMR (76 MHz) spectrum for **8** (using $CDCl_3$ as solvent)

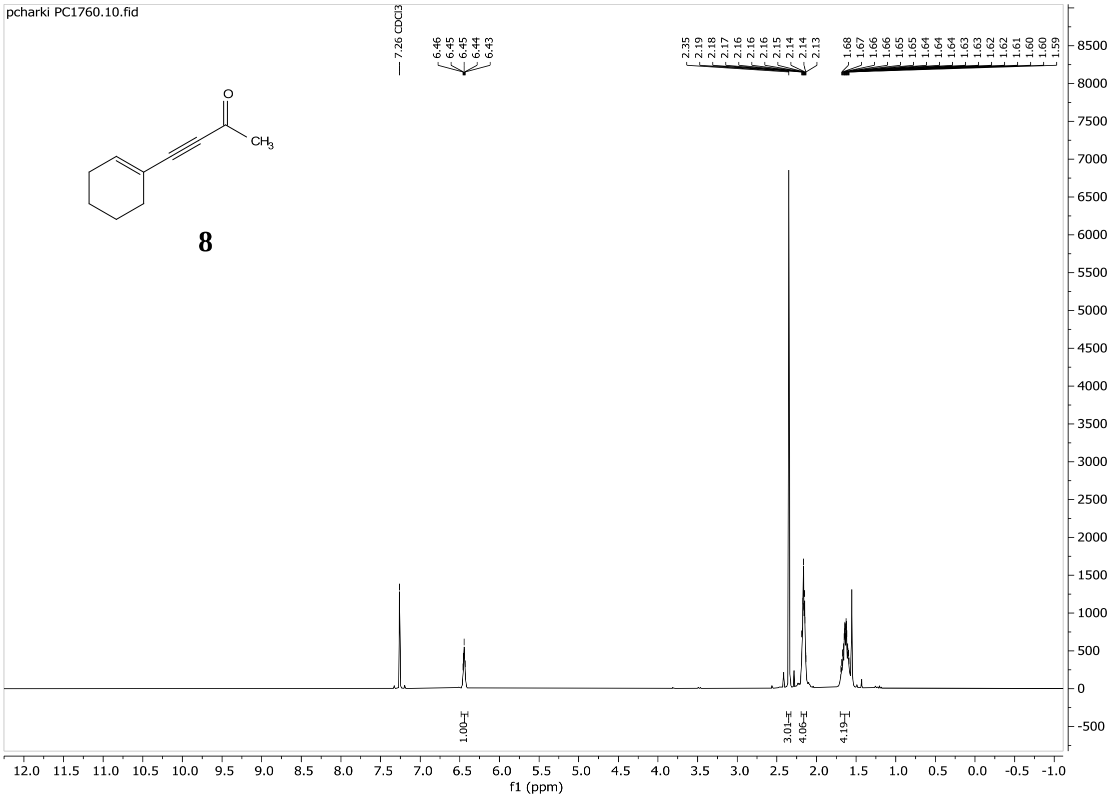

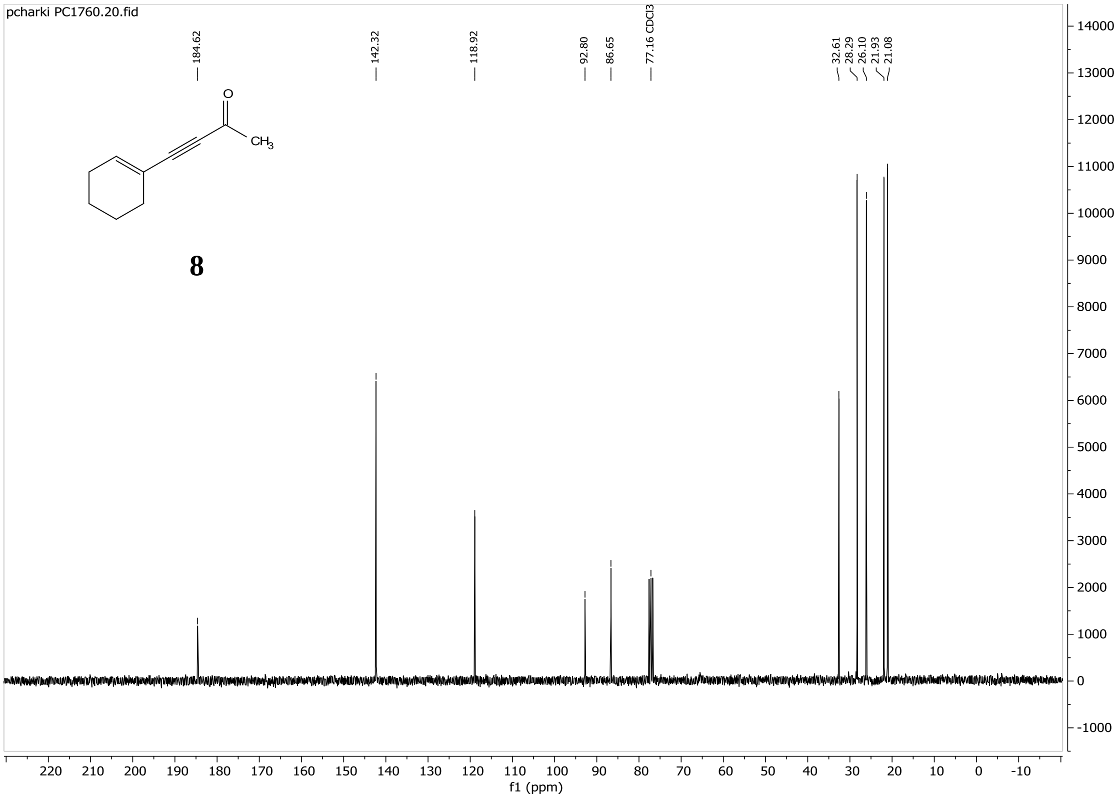

The ^{1}H NMR (300 MHz) and $^{13}C\{^{1}H\}$ NMR (76 MHz) spectrum for **9** (using $CDCl_3$ as solvent)

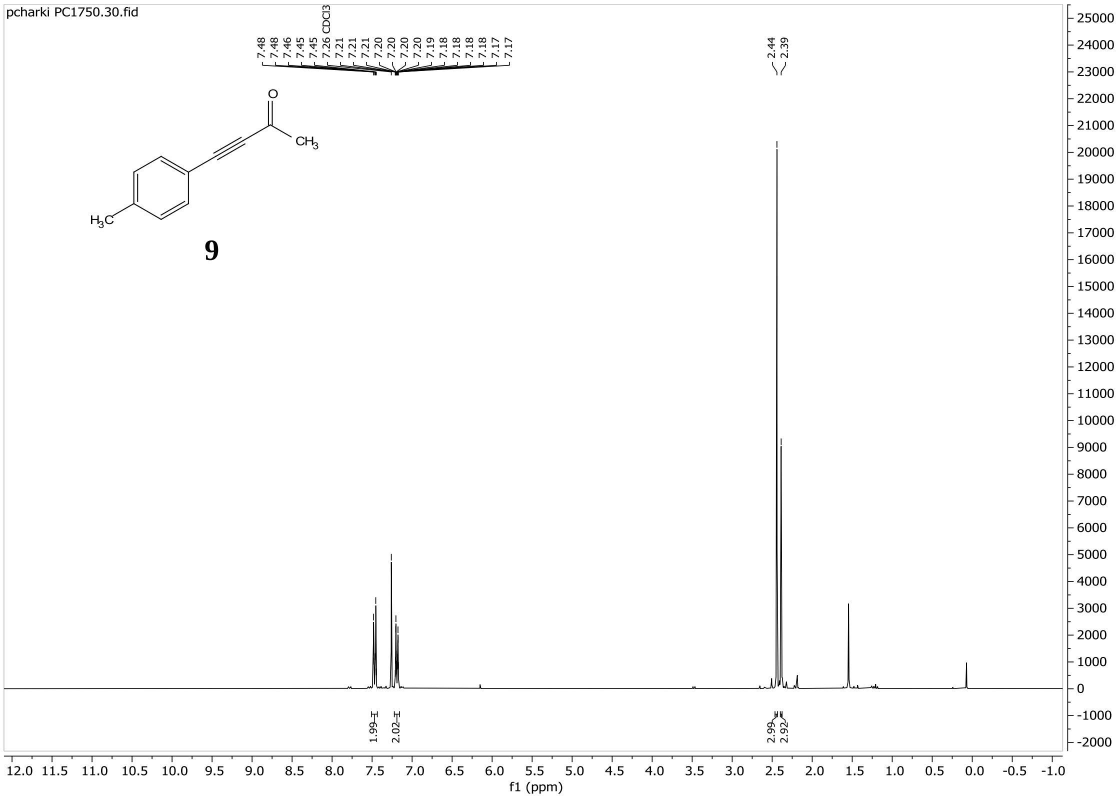

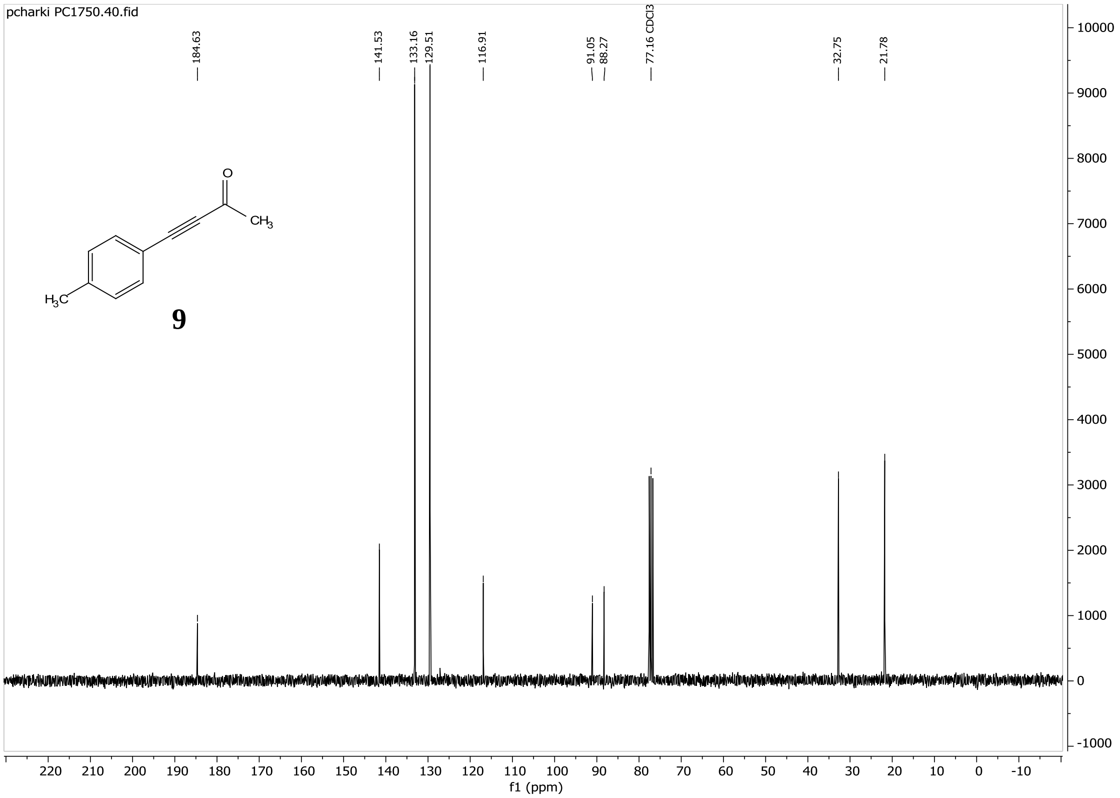

The ^{1}H NMR (300 MHz) and $^{13}C\{^{1}H\}$ NMR (76 MHz) spectrum for **10** (using $CDCl_3$ as solvent)

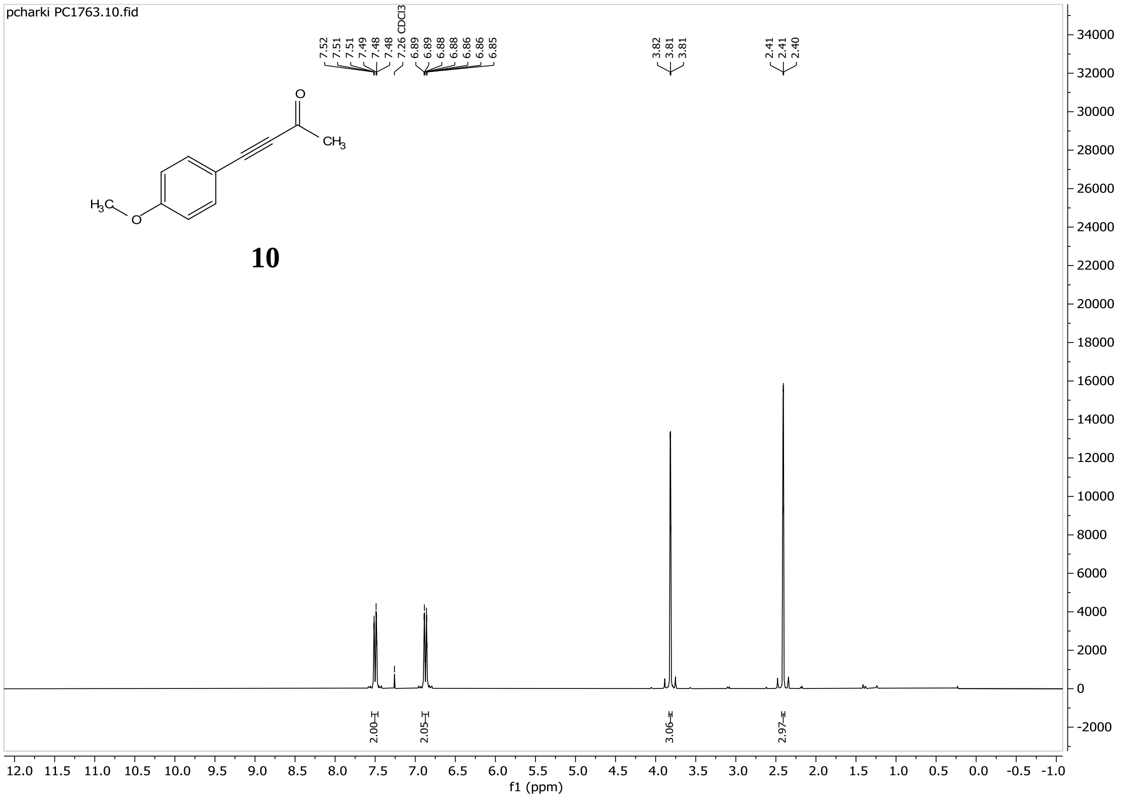

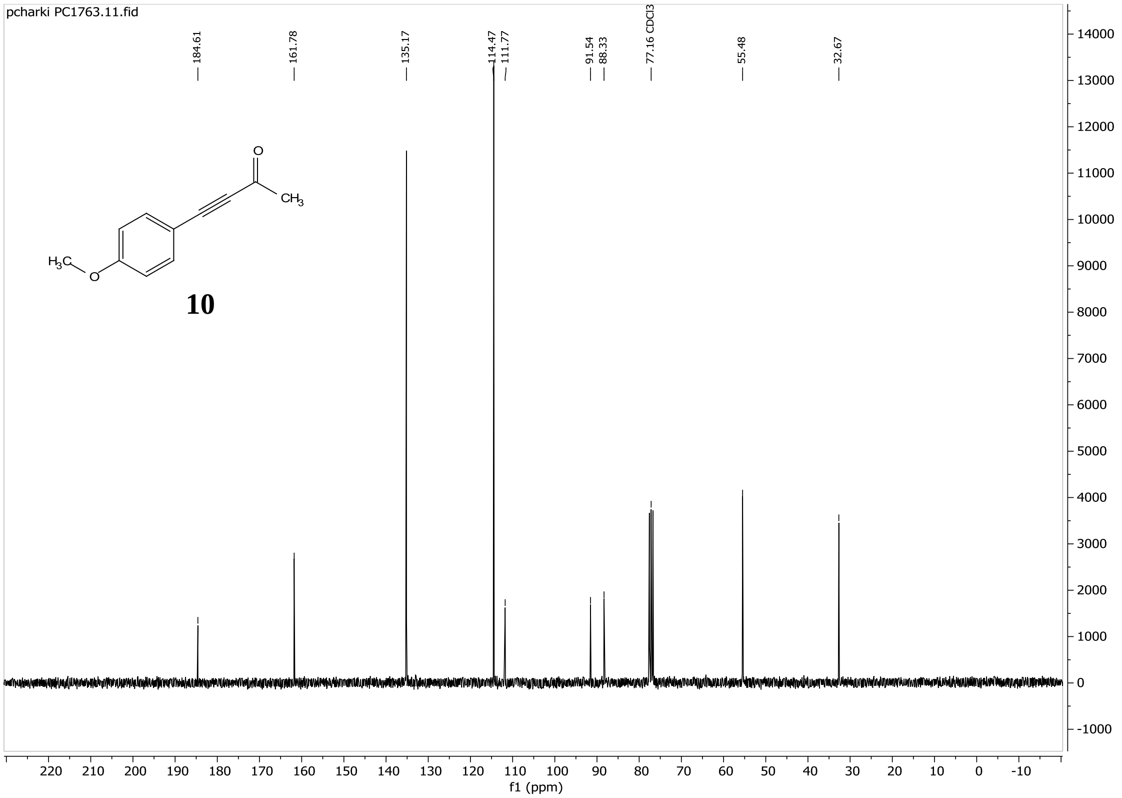

The ^{1}H NMR (300 MHz) and $^{13}C\{^{1}H\}$ NMR (76 MHz) spectrum for **11** (using $CDCl_3$ as solvent)

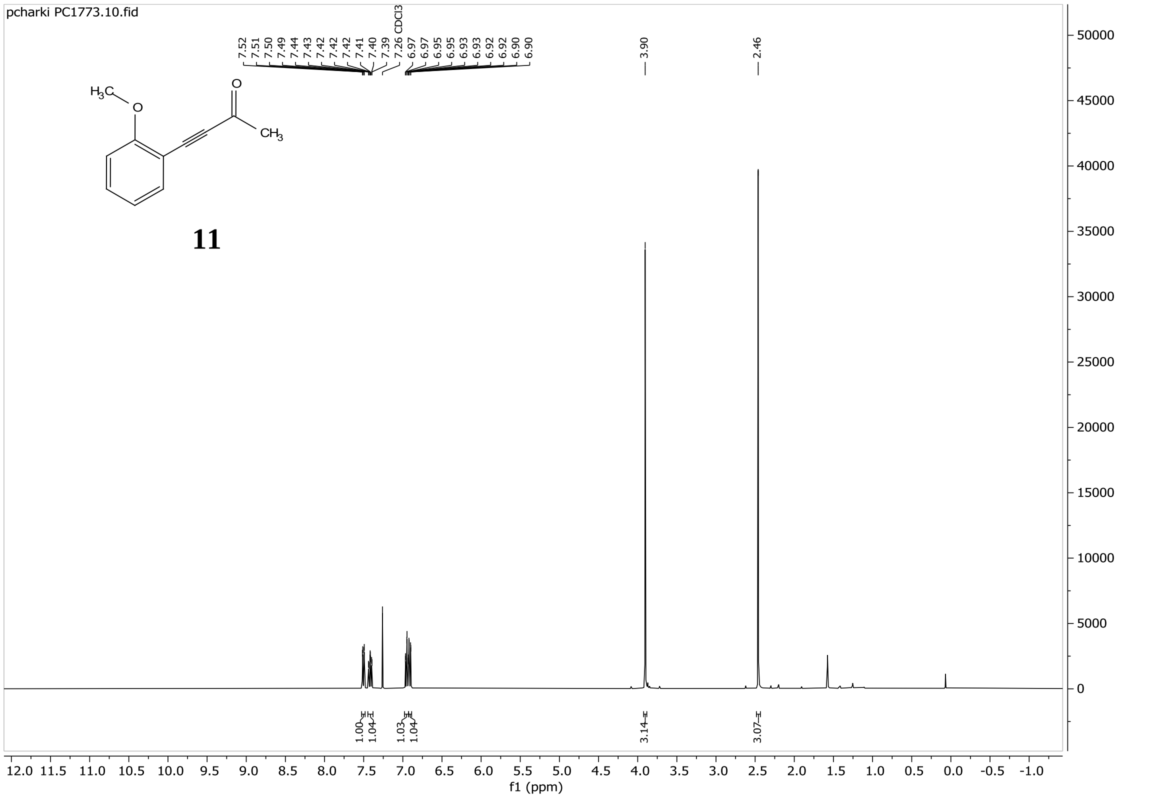

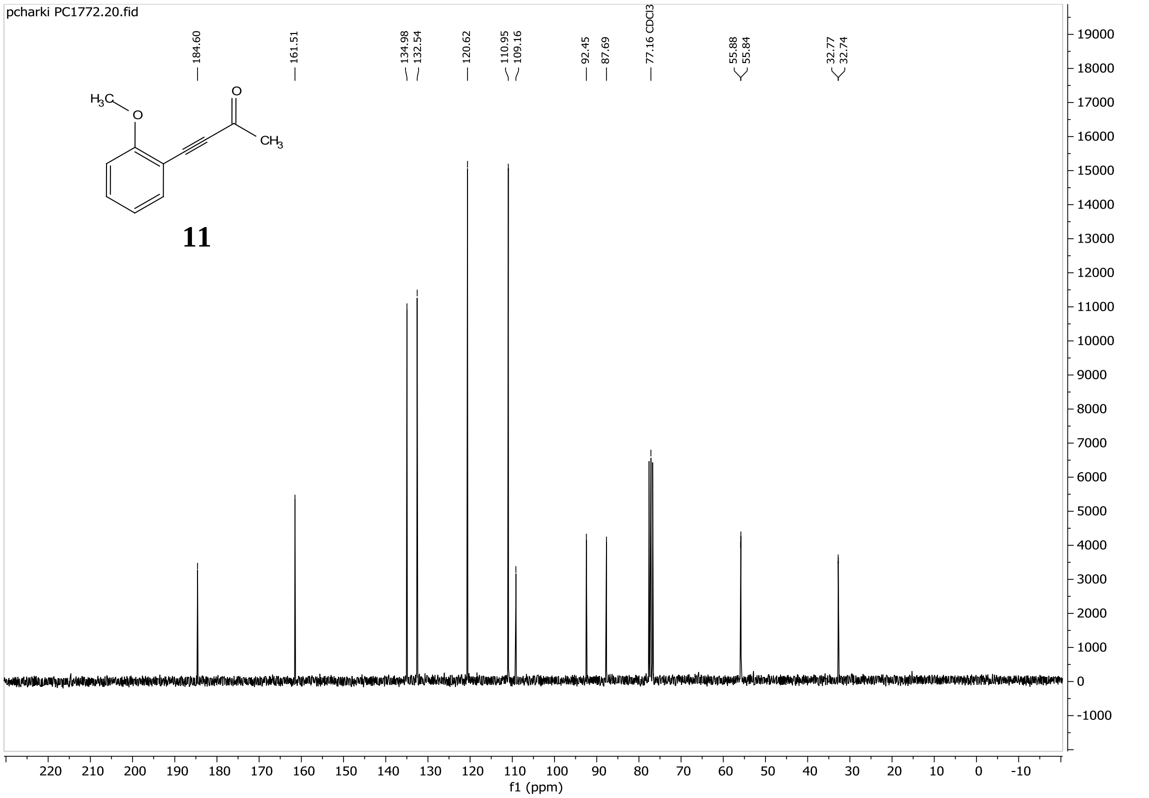

The ^{1}H NMR (300 MHz) and $^{13}C\{^{1}H\}$ NMR (76 MHz) spectrum for **12** (using $CDCl_3$ as solvent)

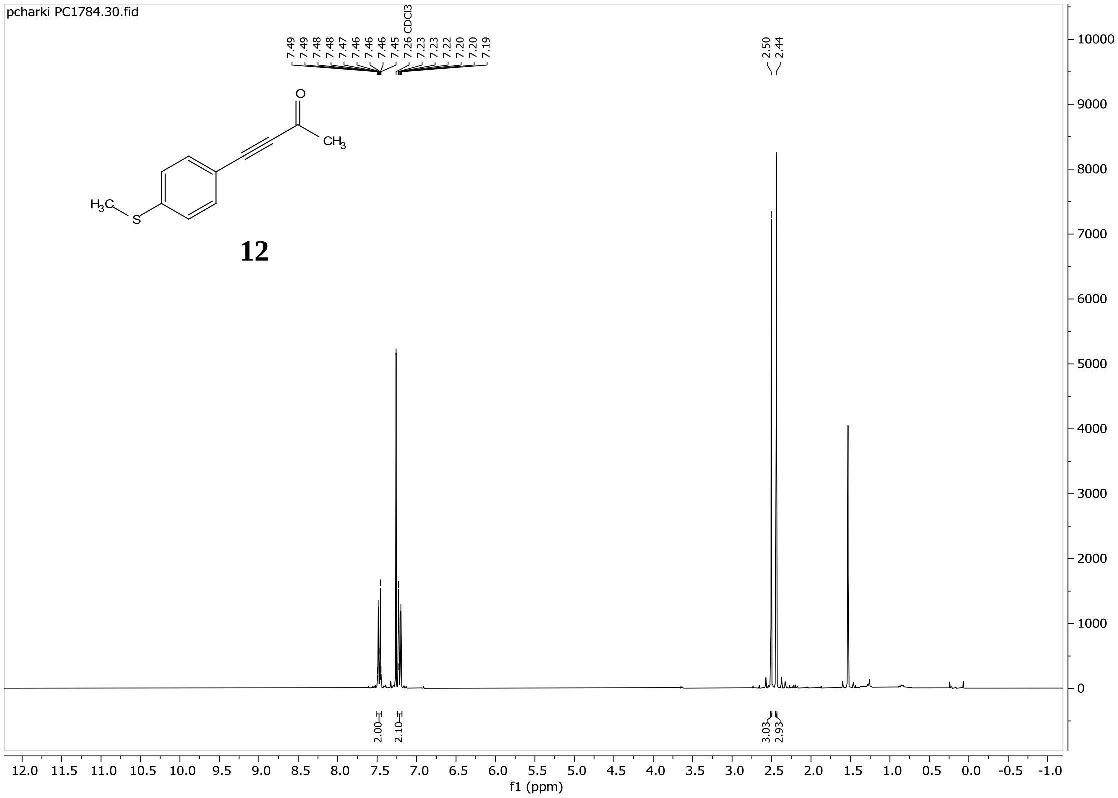

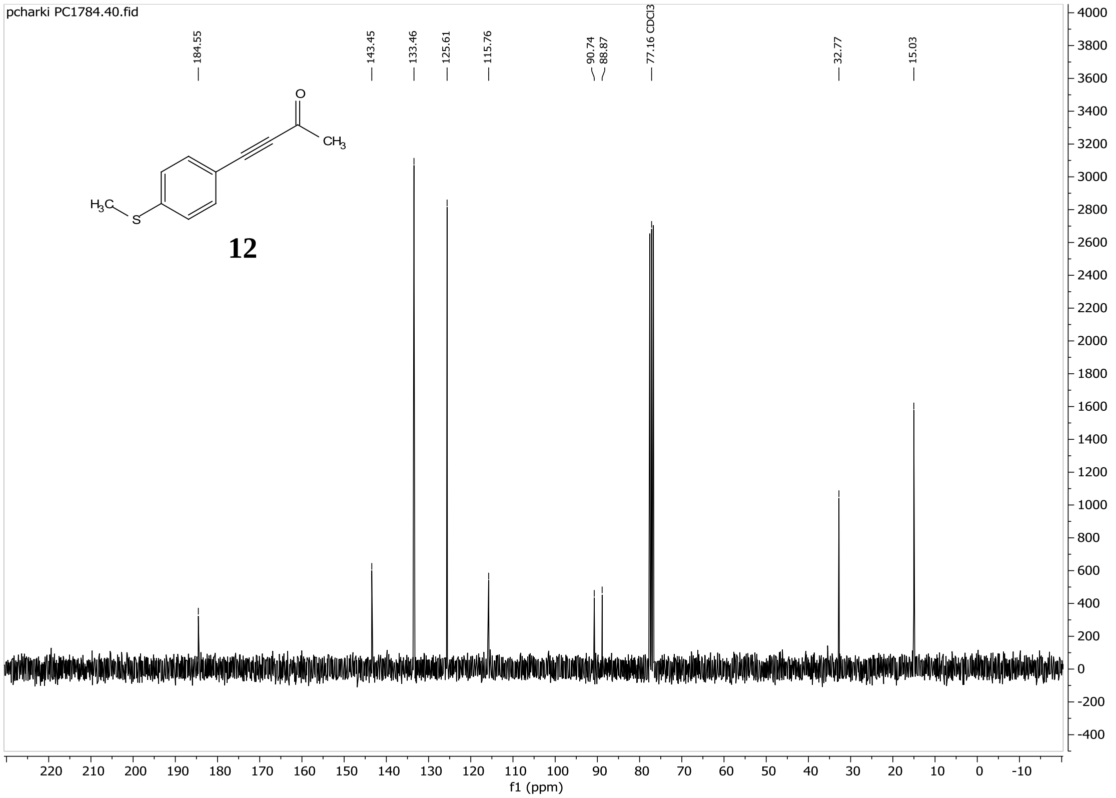

The ^{1}H NMR (300 MHz) and $^{13}C\{^{1}H\}$ NMR (76 MHz) spectrum for **13** (using $CDCl_3$ as solvent)

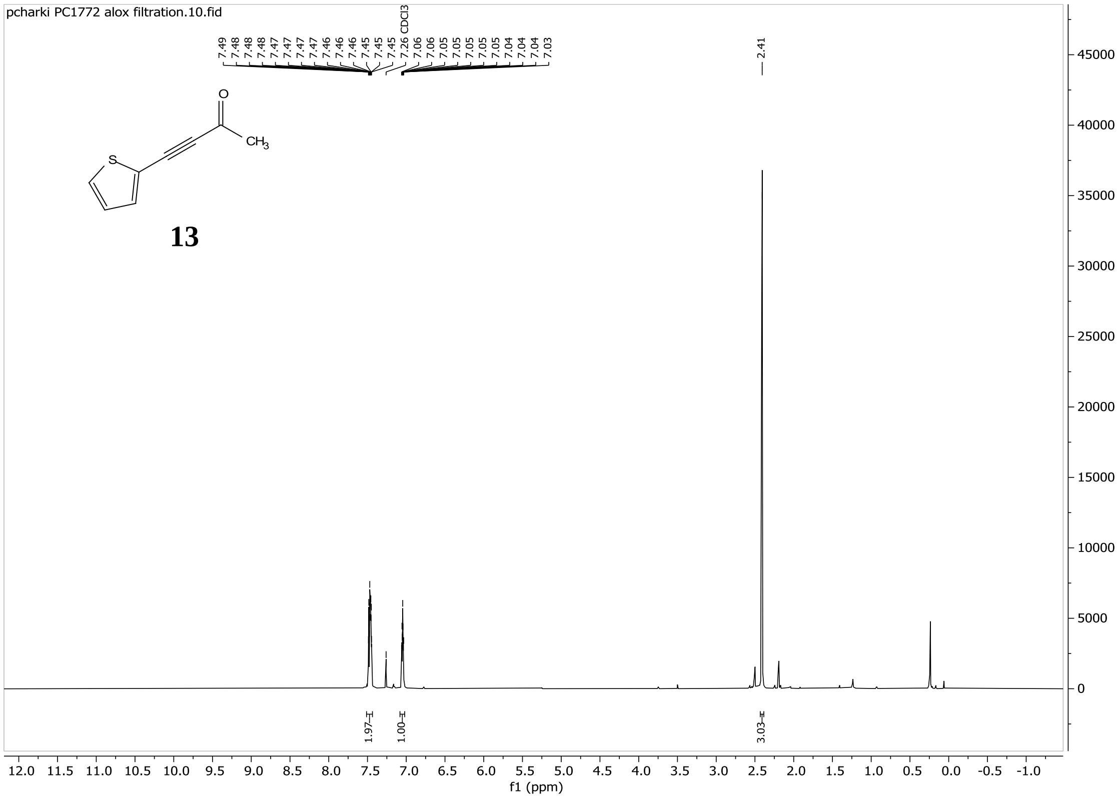

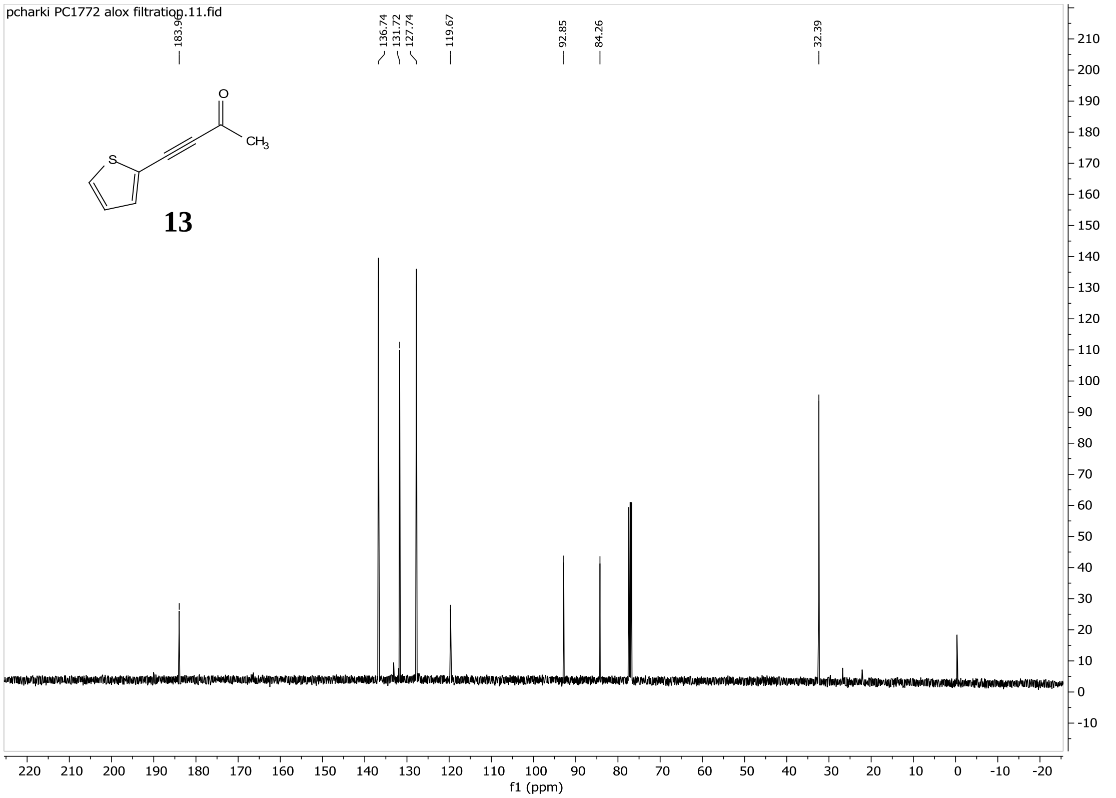

The ^{1}H NMR (300 MHz) and $^{13}C\{^{1}H\}$ NMR (76 MHz) spectrum for **14** (using $CDCl_3$ as solvent)

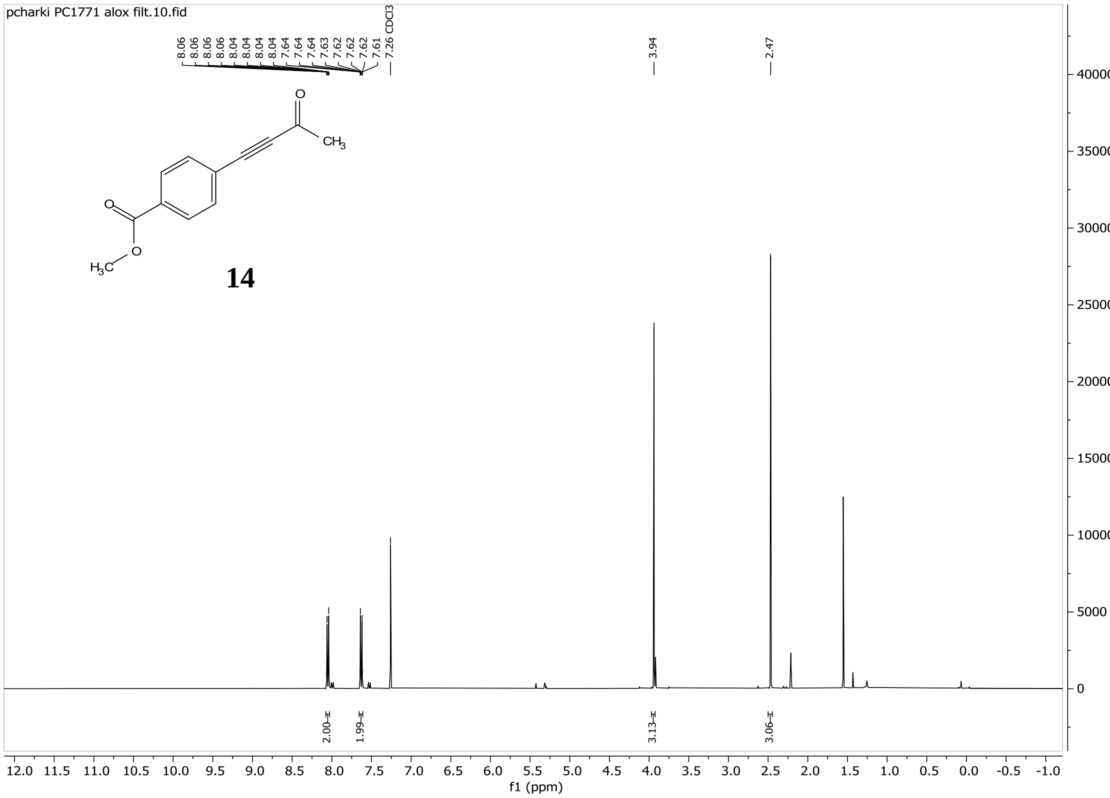

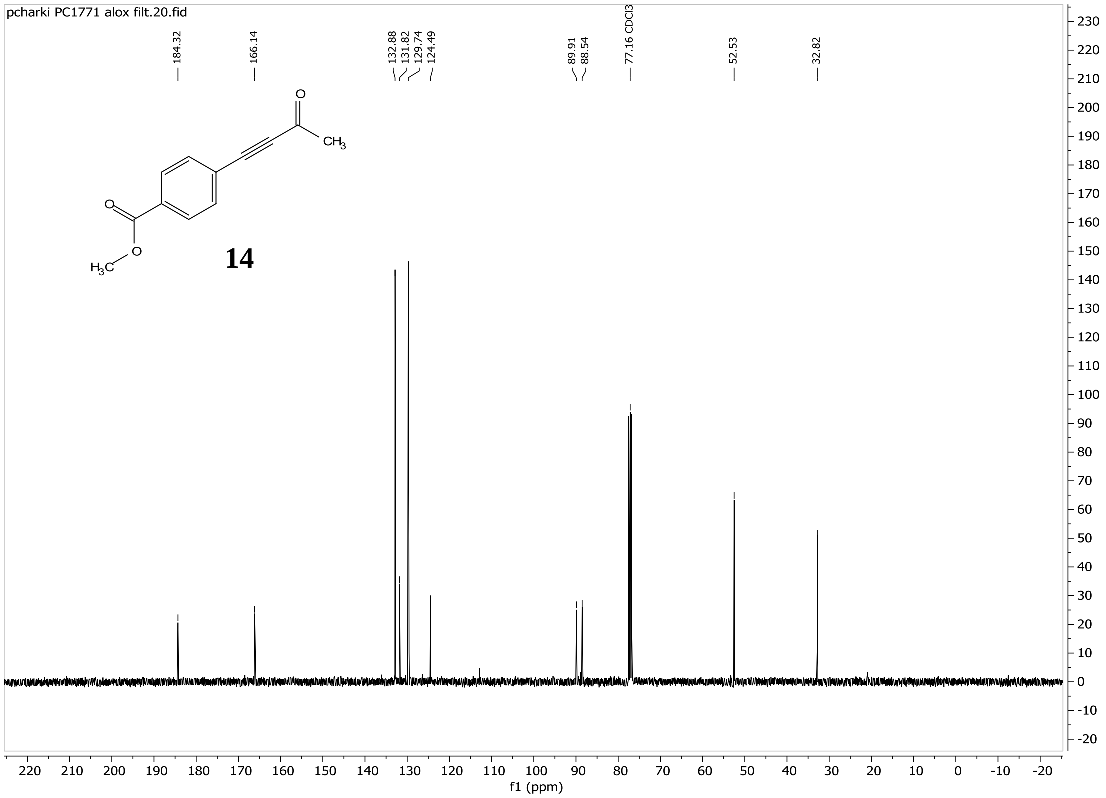

The ^{1}H NMR (300 MHz) and $^{13}C\{^{1}H\}$ NMR (76 MHz) spectrum for **15** (using $CDCl_3$ as solvent)

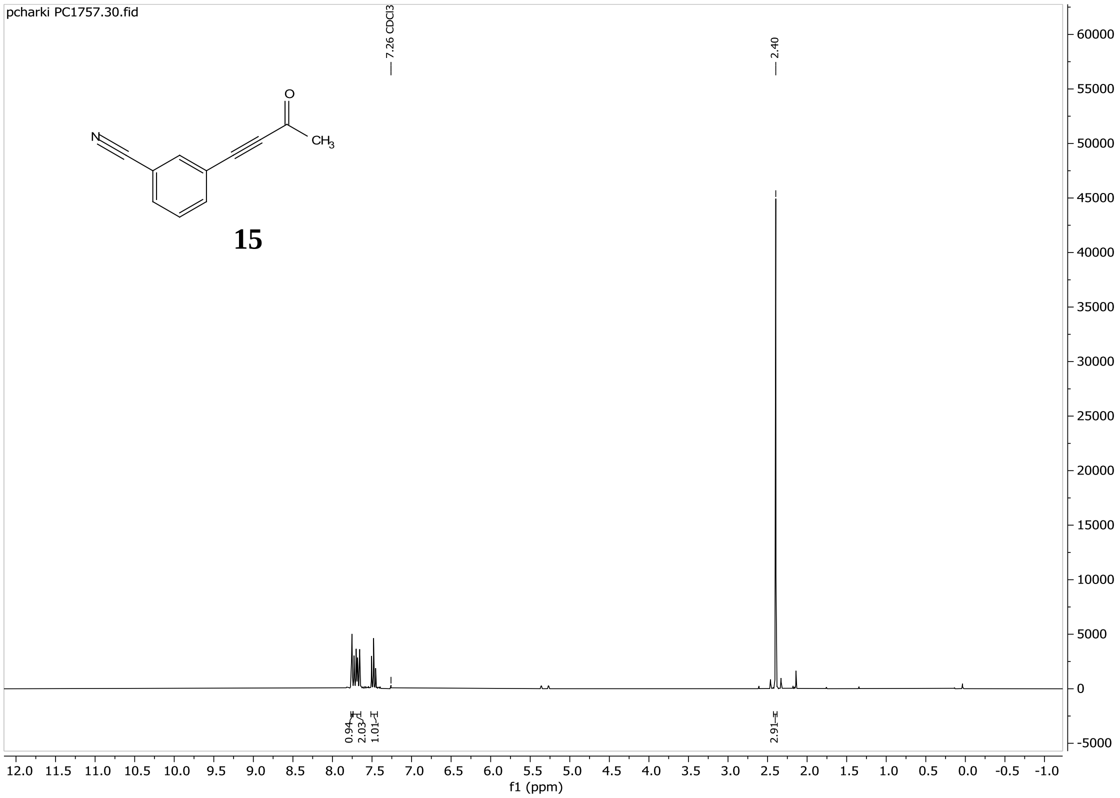

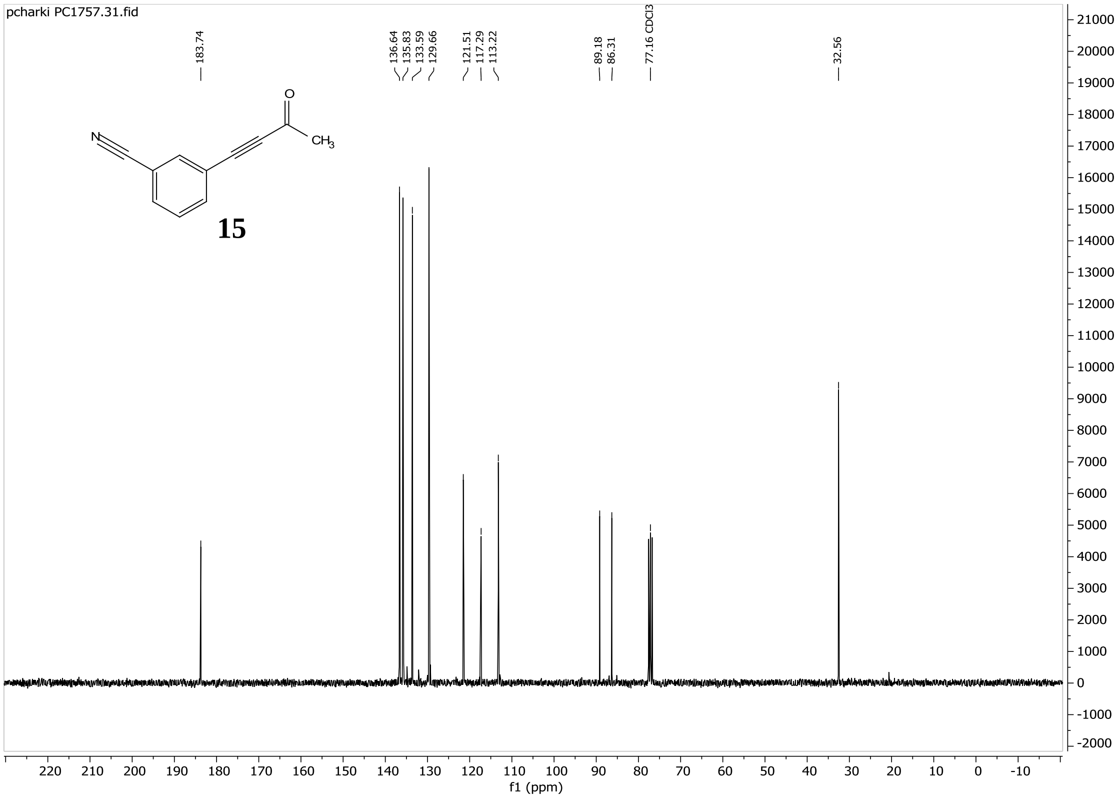

The ^{1}H NMR (300 MHz), $^{13}C\{^{1}H\}$ NMR (76 MHz) and ^{11}B NMR (96 MHz) spectrum for **16** (using $CDCl_3$ as solvent)

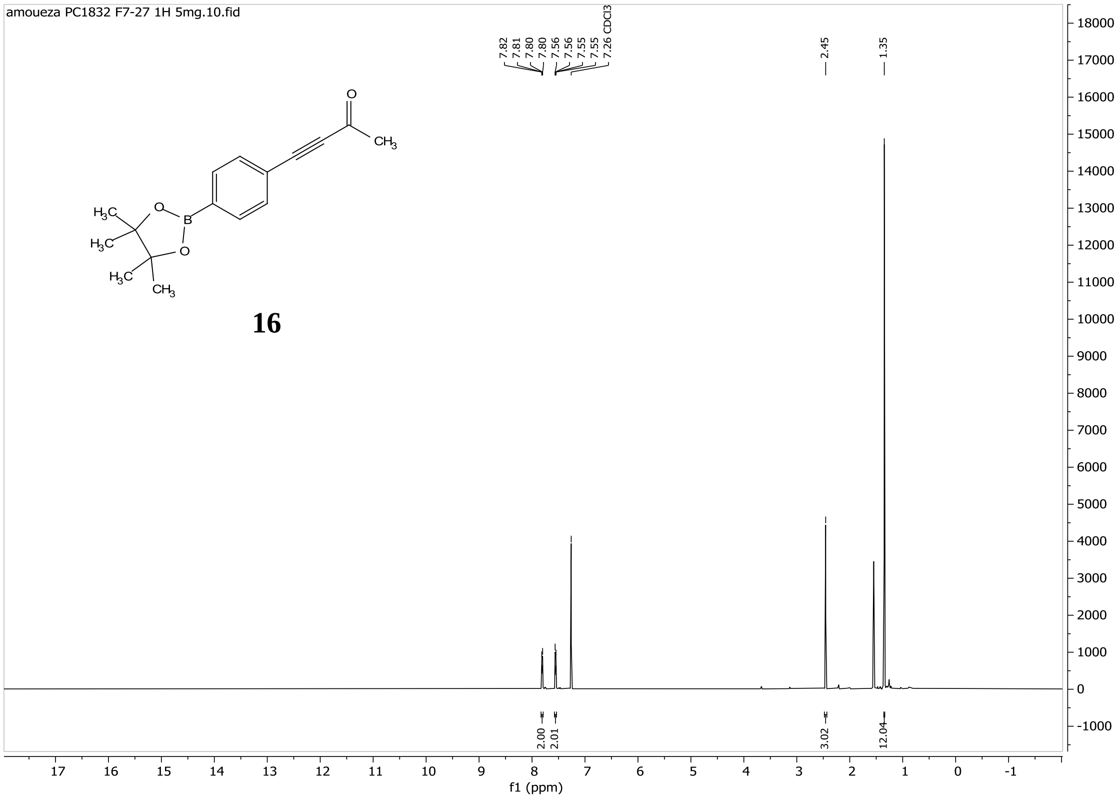

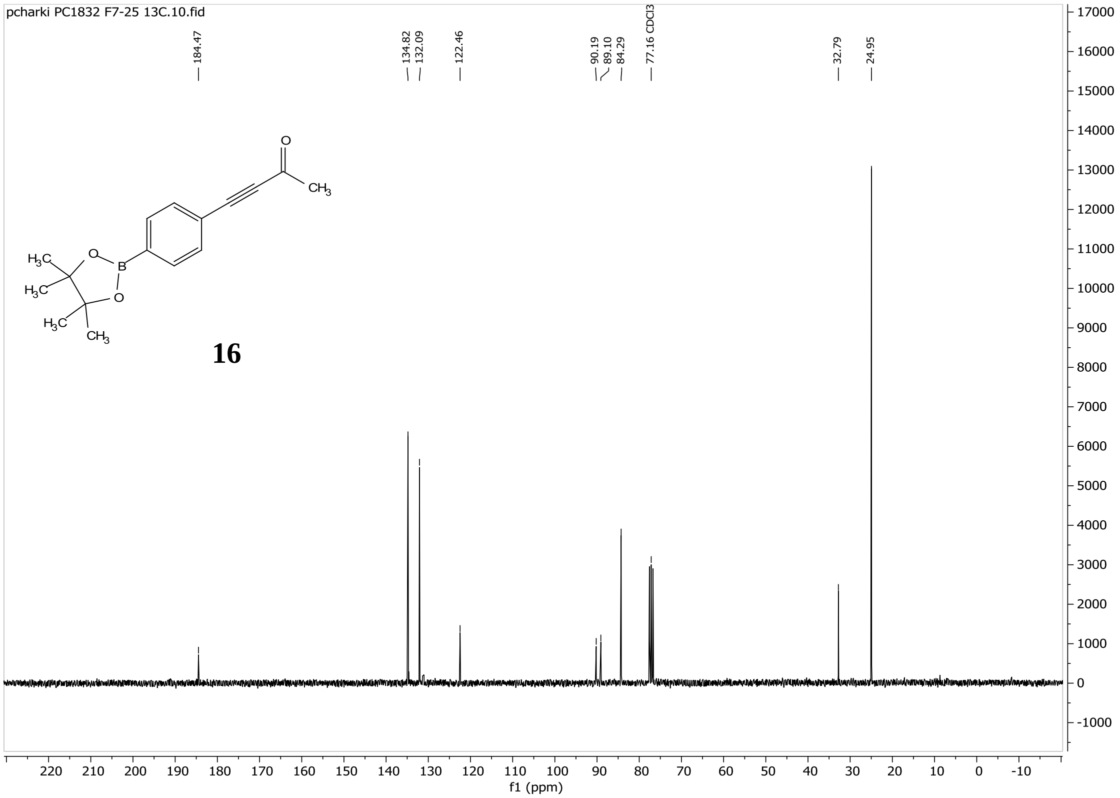

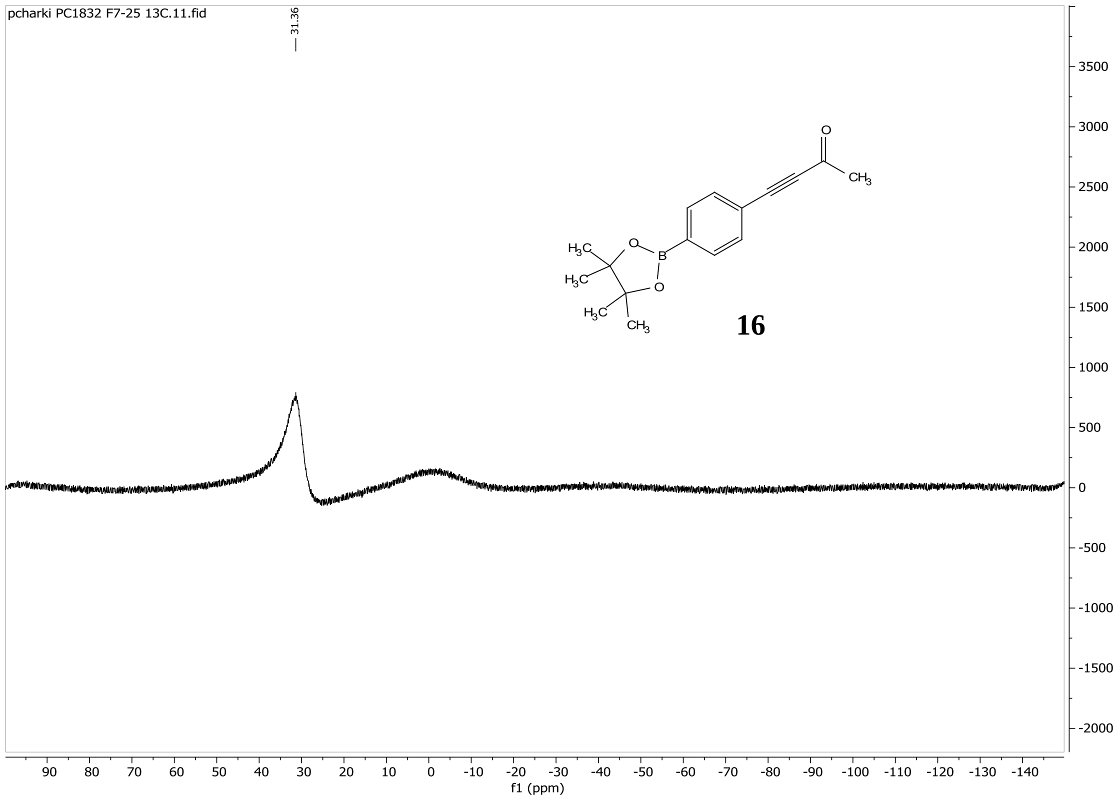

16

The ^{1}H NMR (300 MHz), ^{13}C{^{1}H} NMR (76 MHz) and ^{11}B NMR (96 MHz) spectrum for **51** (using $CDCl_3$ as solvent)

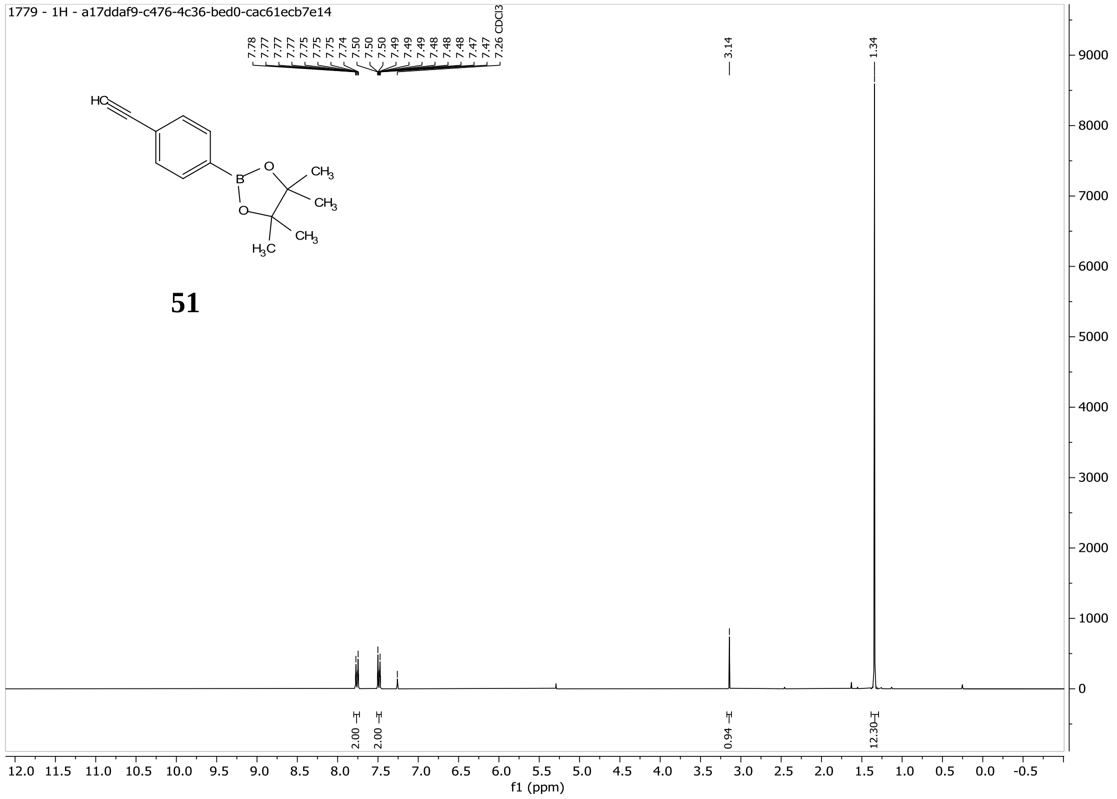

51

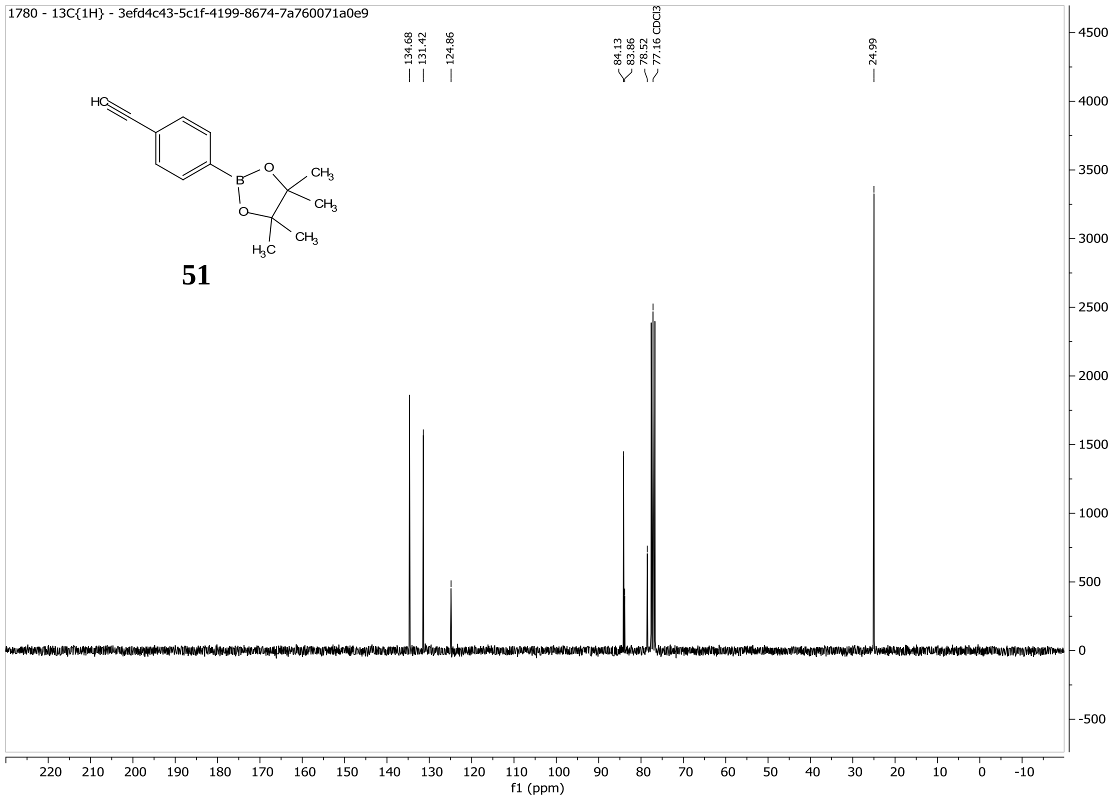
1780 - 13C{1H} - 3efd4c43-5c1f-4199-8674-7a760071a0e9
134.68
131.42
124.86
84.13
83.86
78.52
77.16 CDCl3
24.99
HC
B
O
CH3
H3C
f1 (ppm)

51

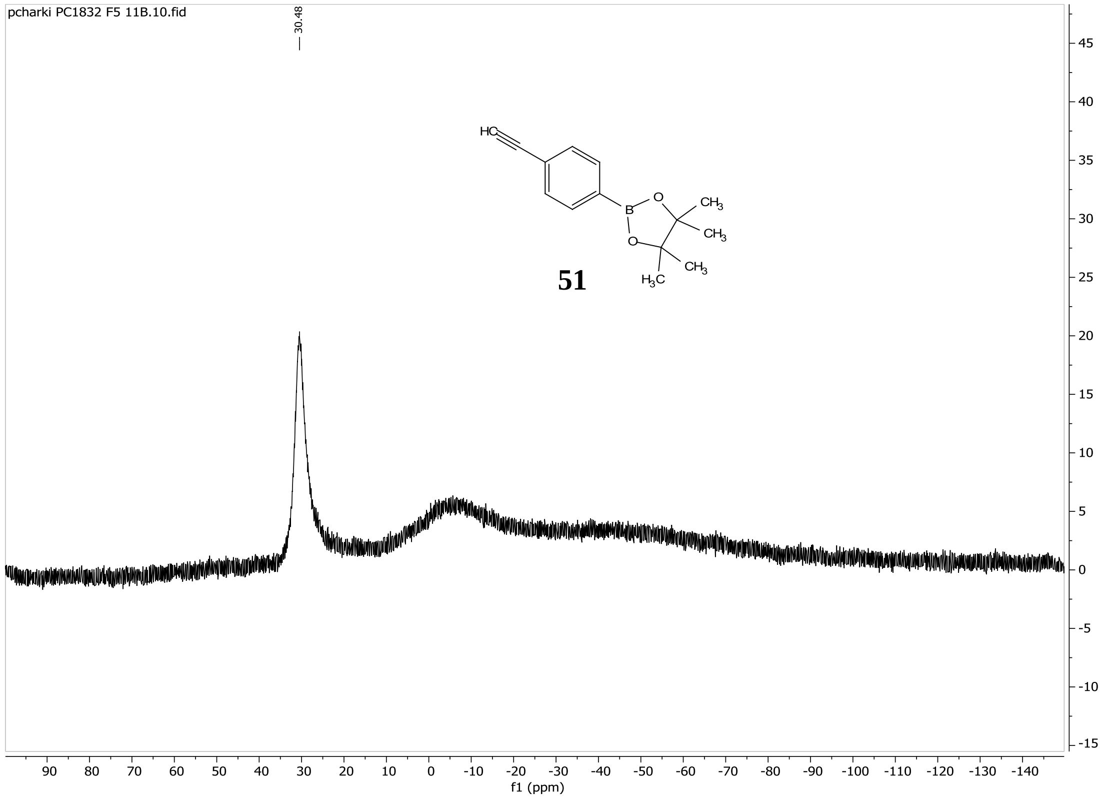
pcharki PC1832 F5 11B.10.fid
30.48
HC
B
O
CH3
H3C
f1 (ppm)

51

The ^{1}H NMR (300 MHz) and $^{13}C\{^{1}H\}$ NMR (76 MHz) spectrum for **17** (using $CDCl_3$ as solvent)

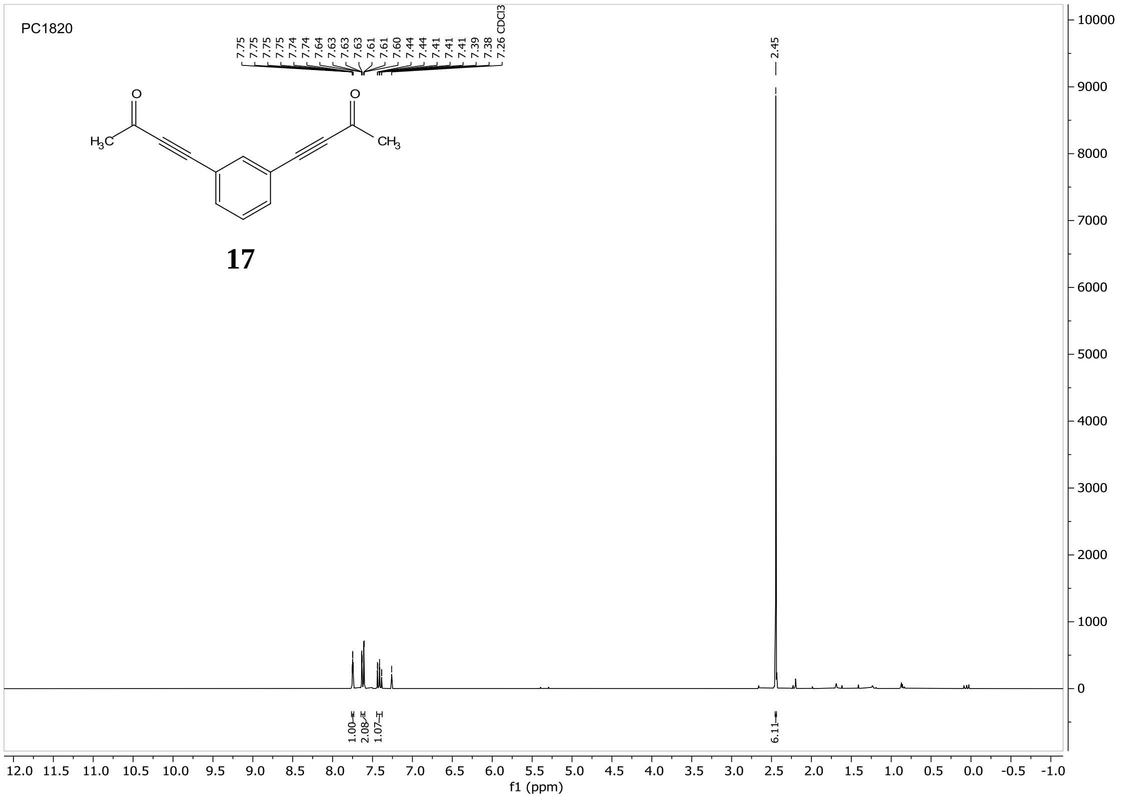

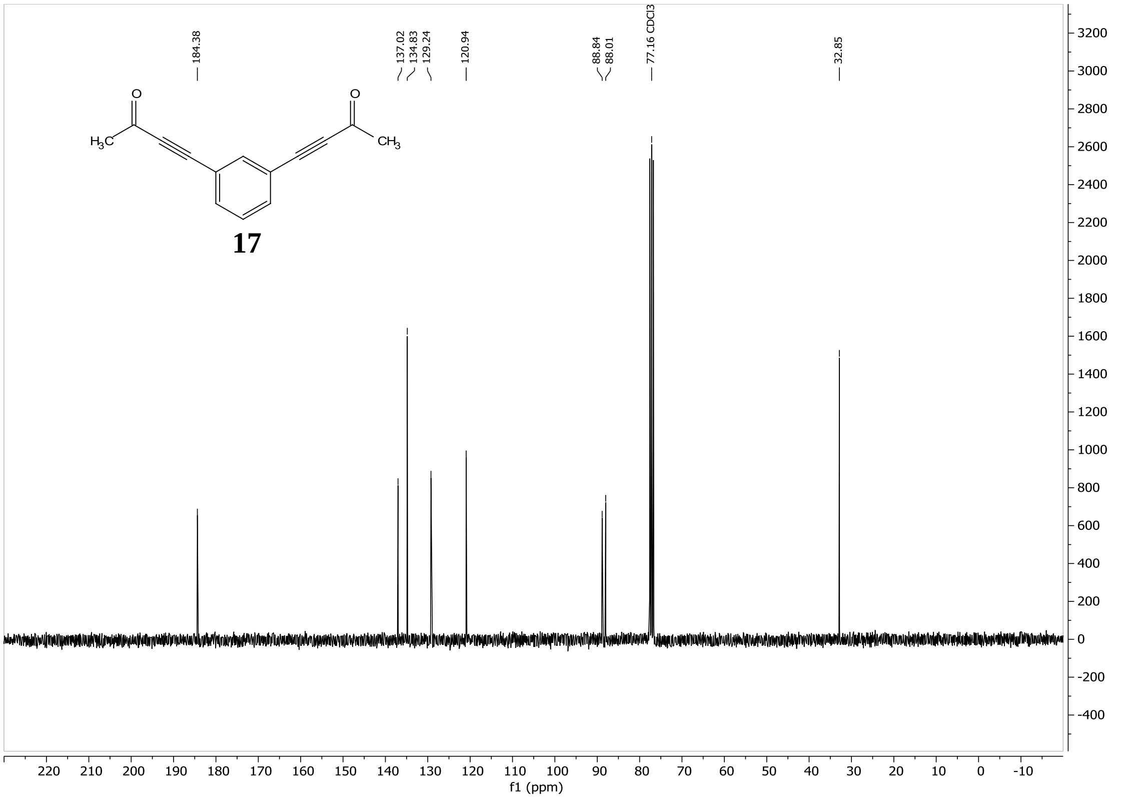

The ^{1}H NMR (300 MHz) and $^{13}C\{^{1}H\}$ NMR (76 MHz) spectrum for **18** (using $CDCl_3$ as solvent)

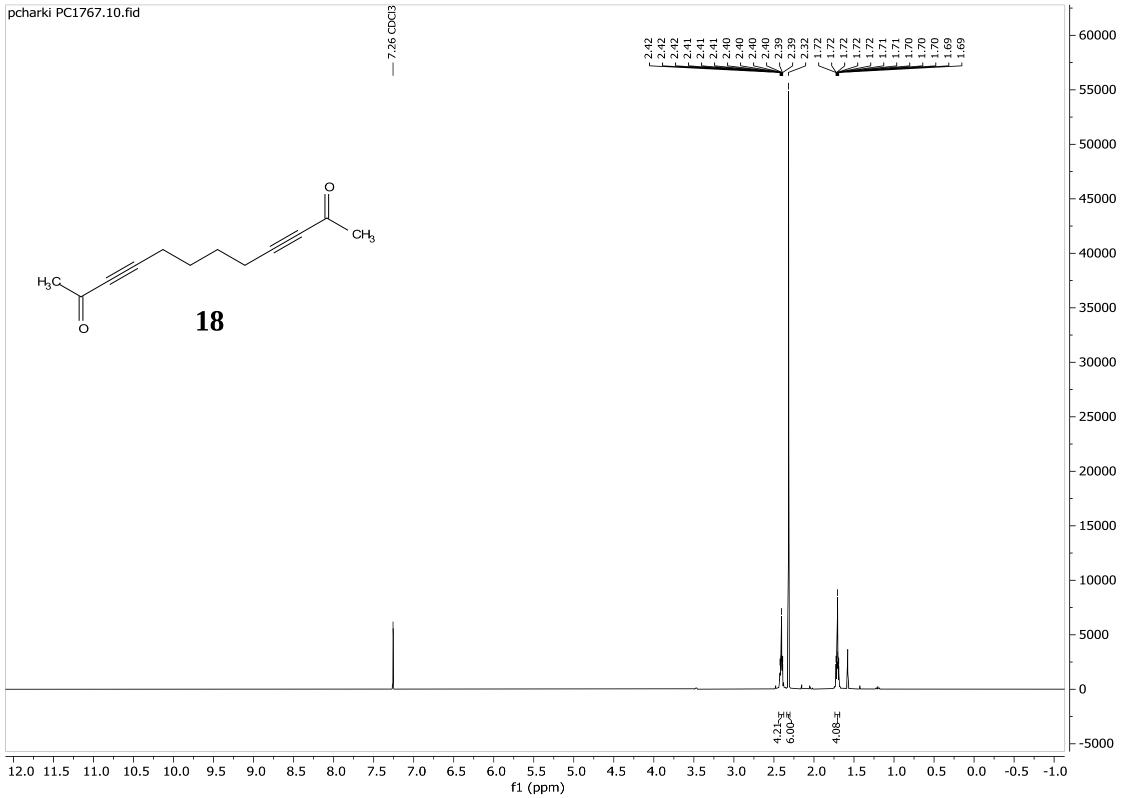

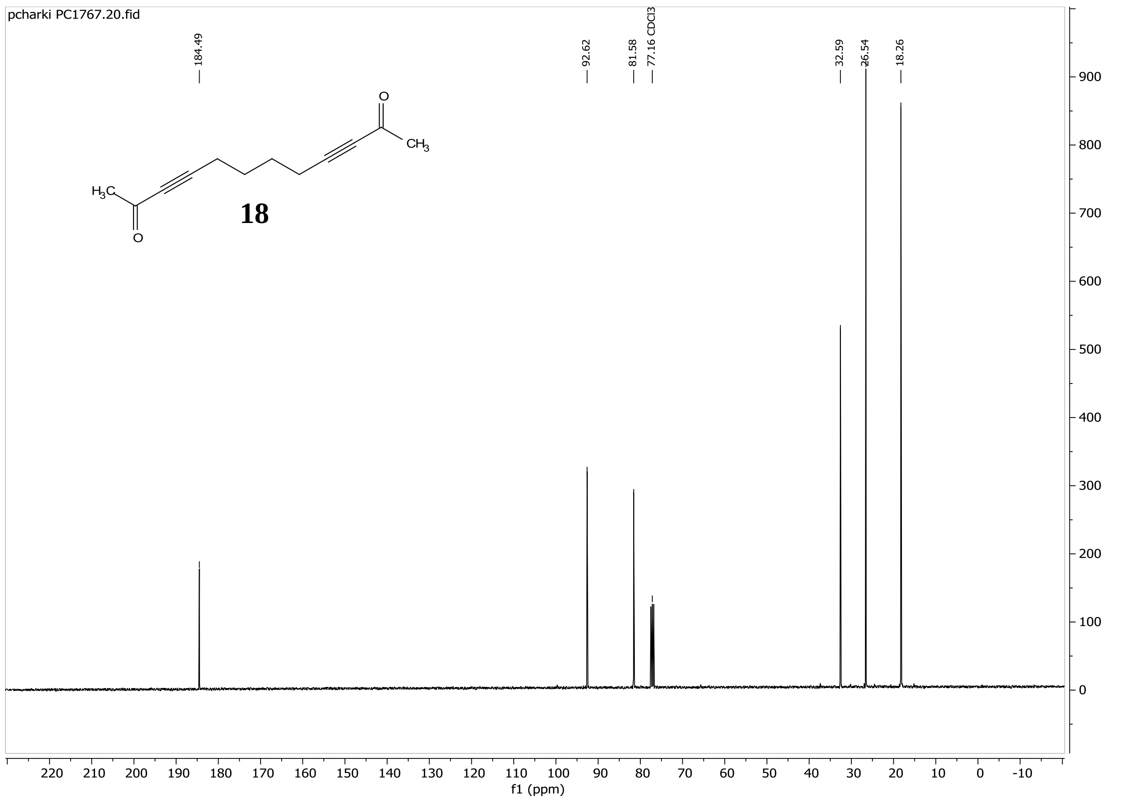

The ^{1}H NMR (300 MHz) and $^{13}C\{^{1}H\}$ NMR (76 MHz) spectrum for **19** (using $CDCl_3$ as solvent)

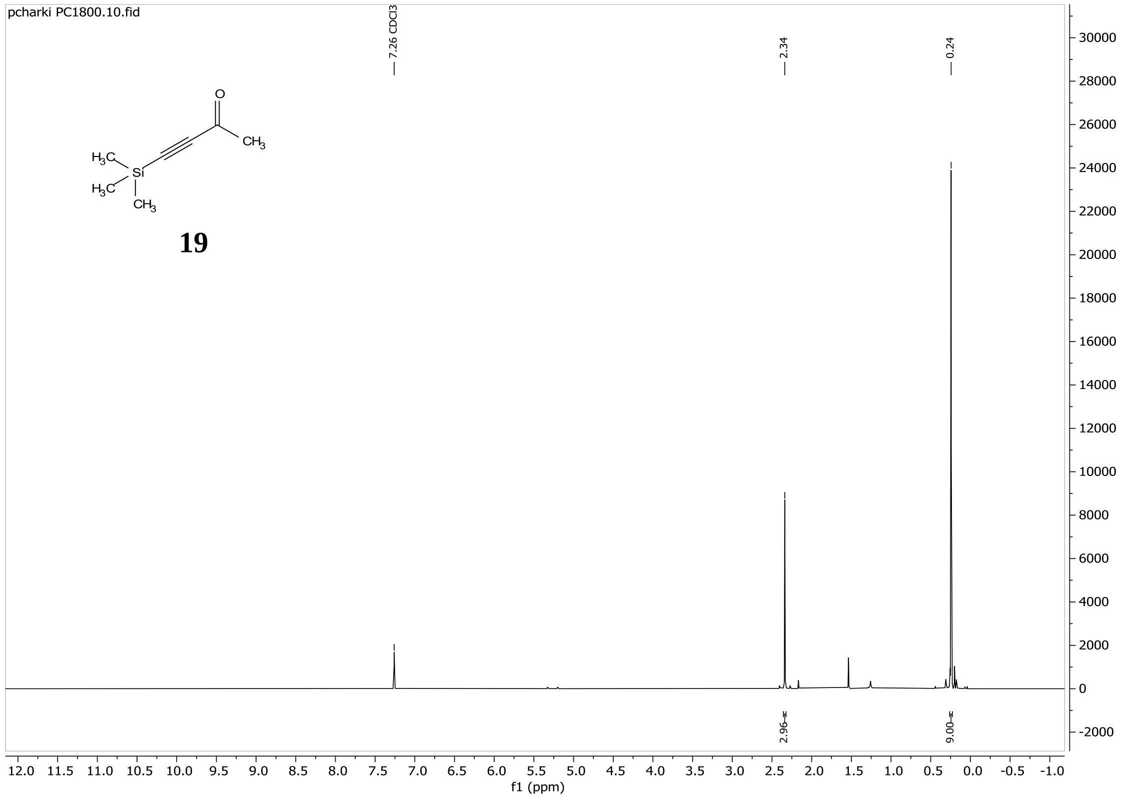

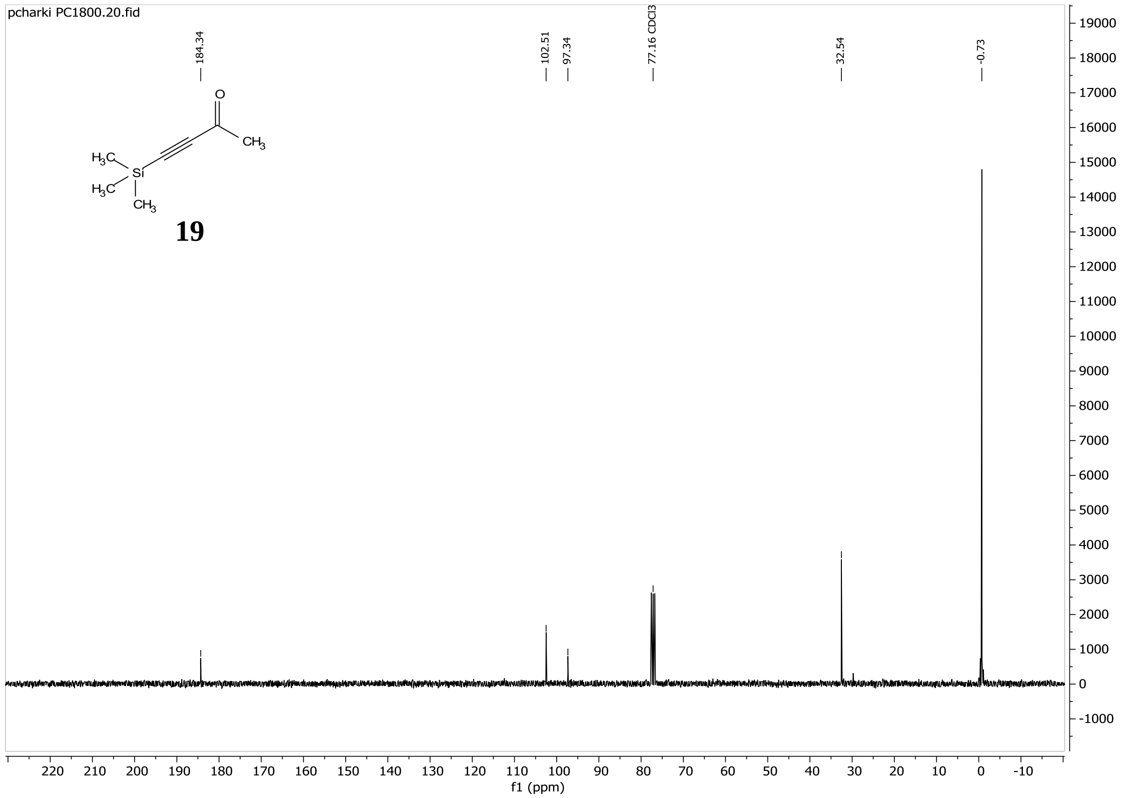

The ^{1}H NMR (300 MHz) and $^{13}C\{^{1}H\}$ NMR (76 MHz) spectrum for **20** (using $CDCl_3$ as solvent)

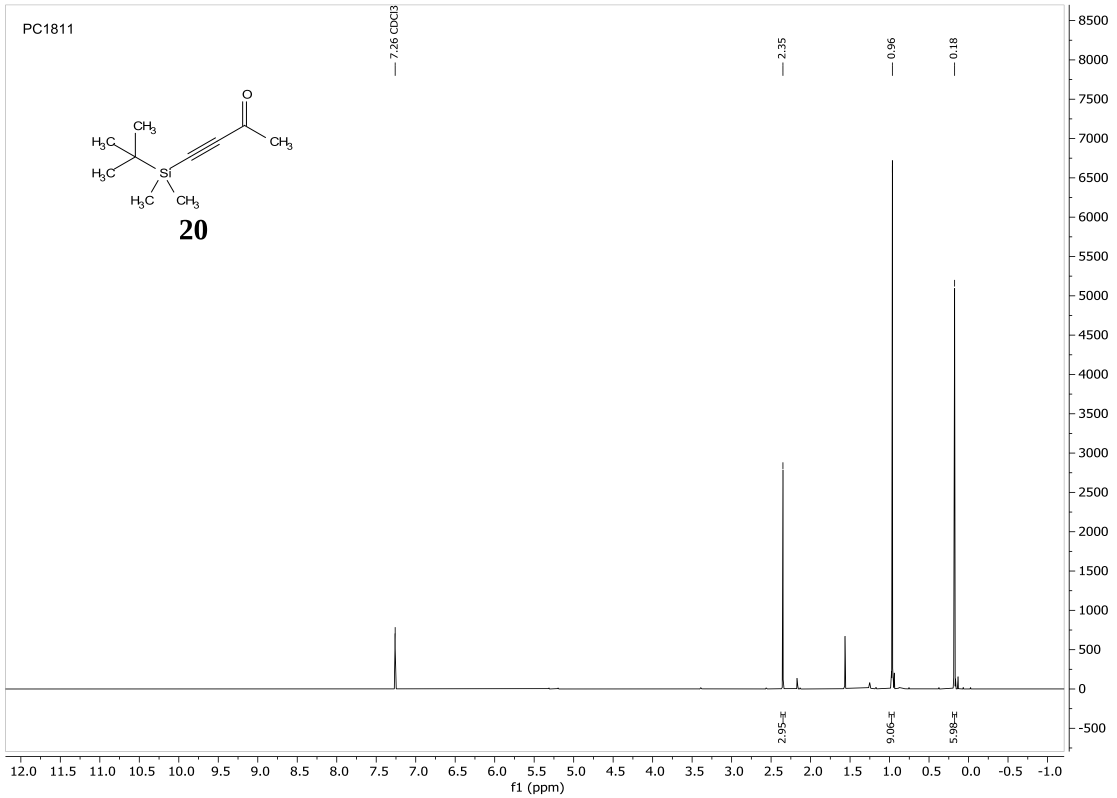

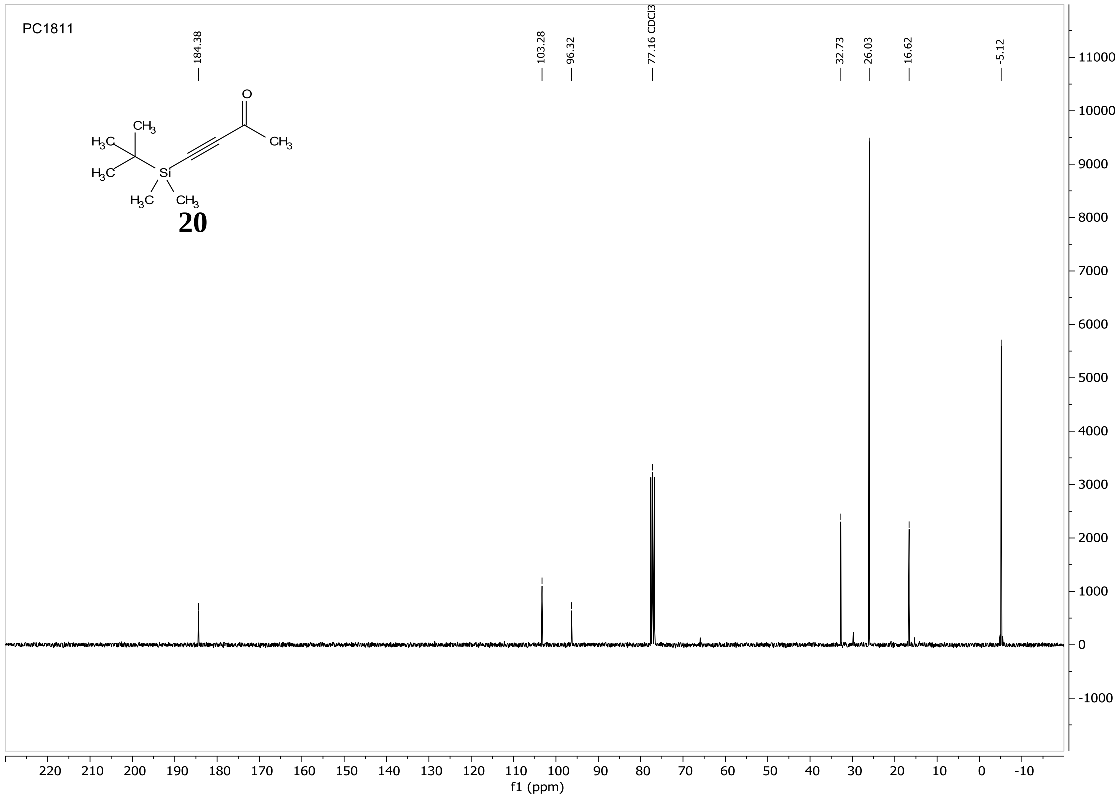

The ^{1}H NMR (300 MHz) and ^{13}C{^{1}H} NMR (76 MHz) spectrum for **21** (using $CDCl_3$ as solvent)

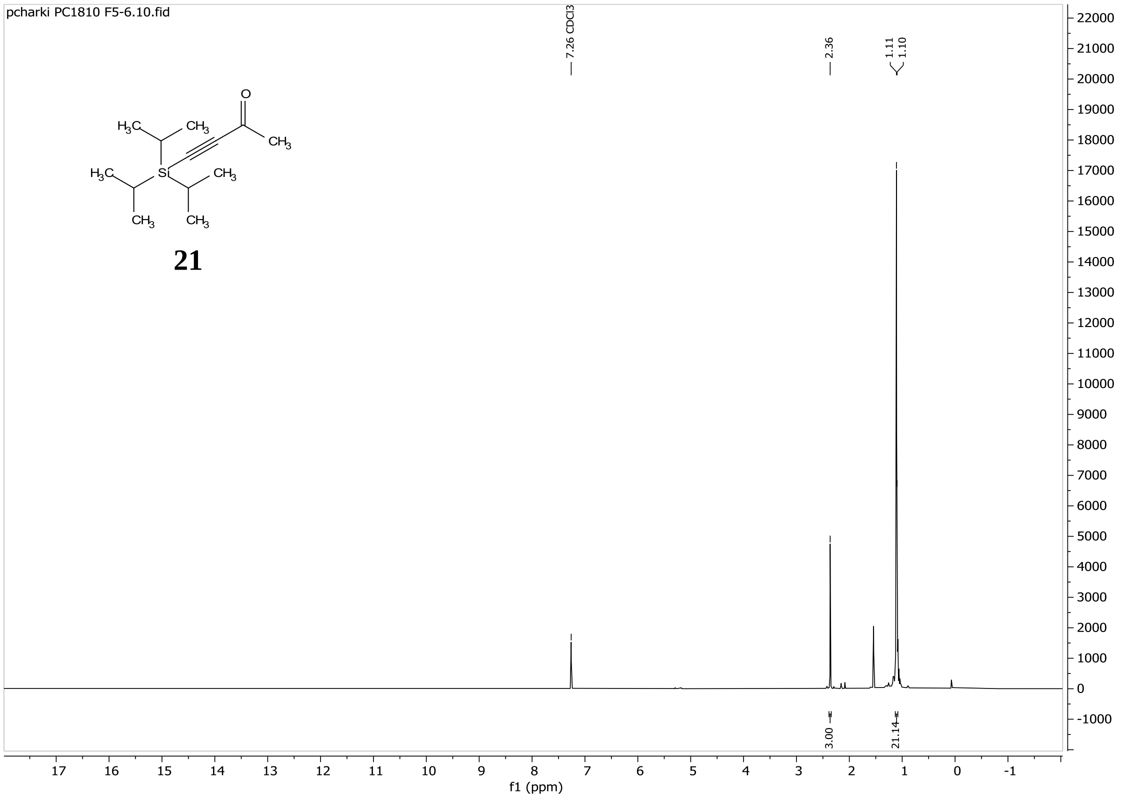

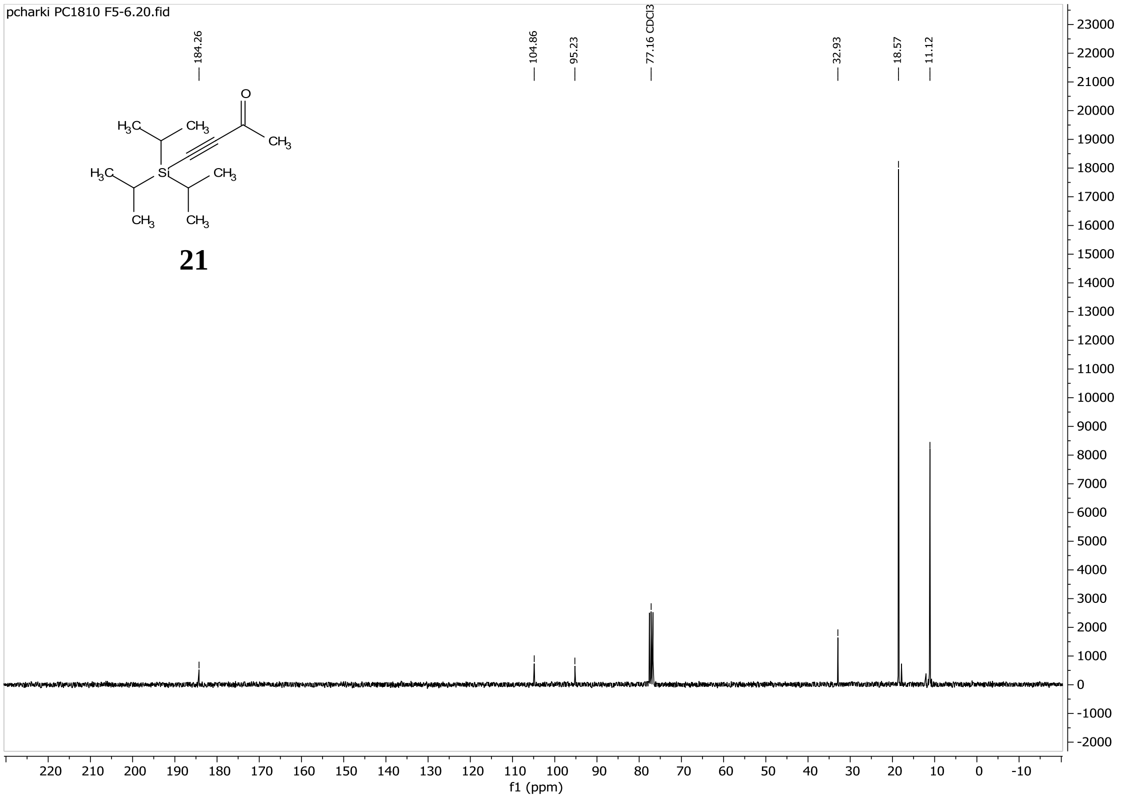

The ^{1}H NMR (300 MHz) and ^{13}C{^{1}H} NMR (76 MHz) spectrum for **22** (using $CDCl_3$ as solvent)

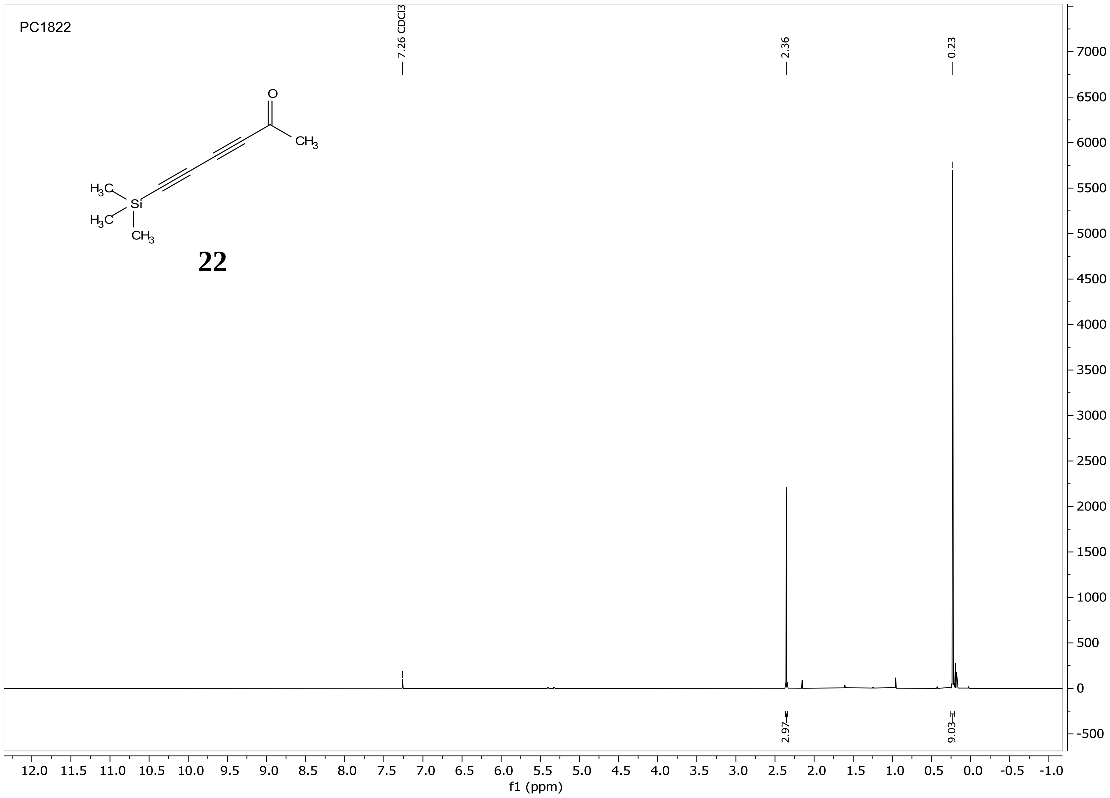

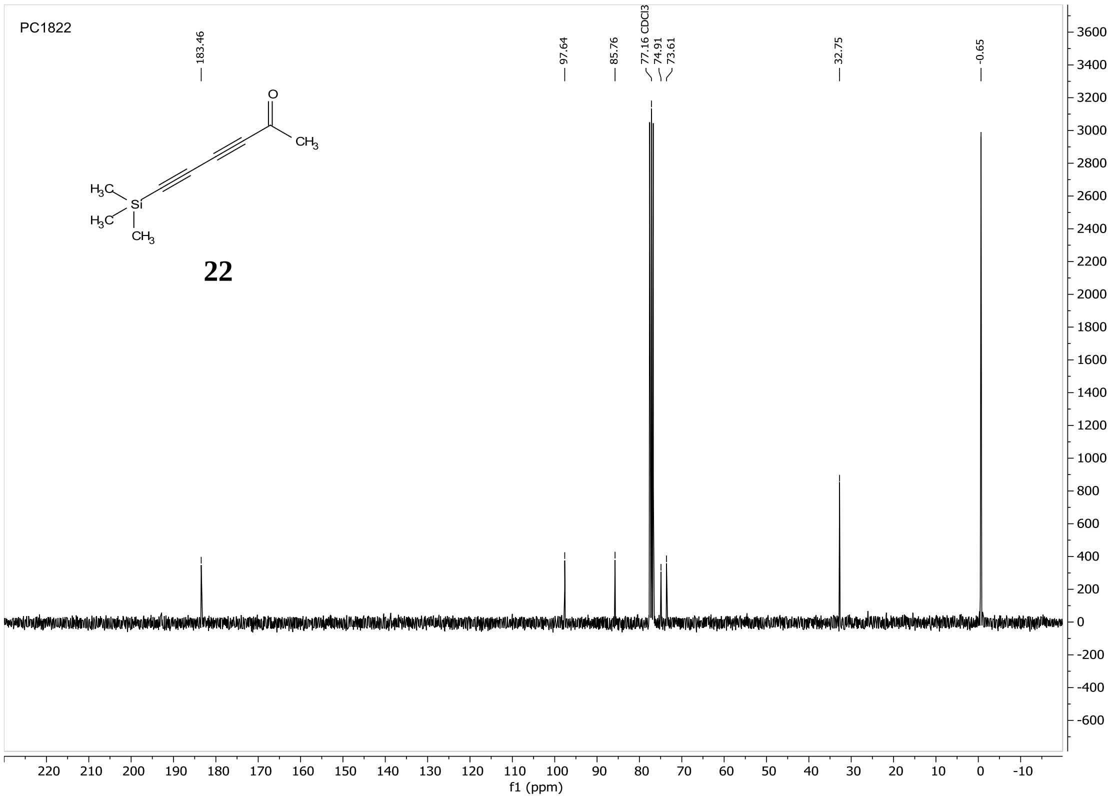

The ^{1}H NMR (300 MHz) and $^{13}C\{^{1}H\}$ NMR (76 MHz) spectrum for **23** (using $CDCl_3$ as solvent)

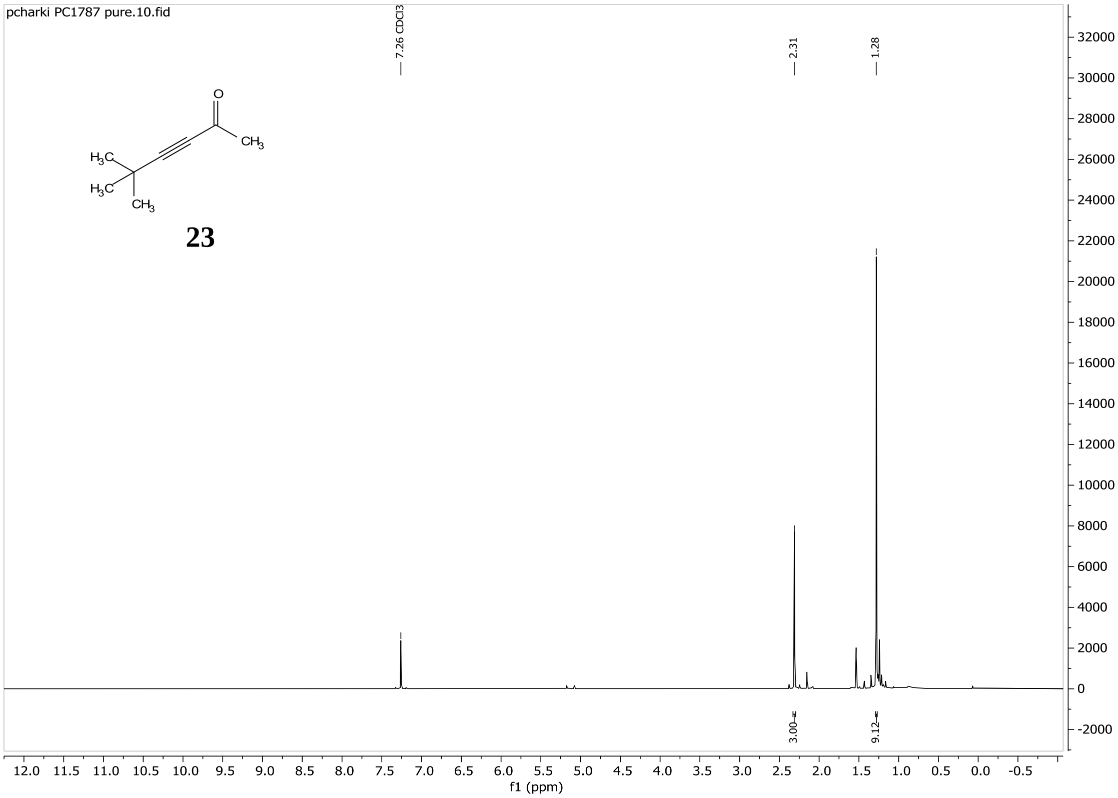

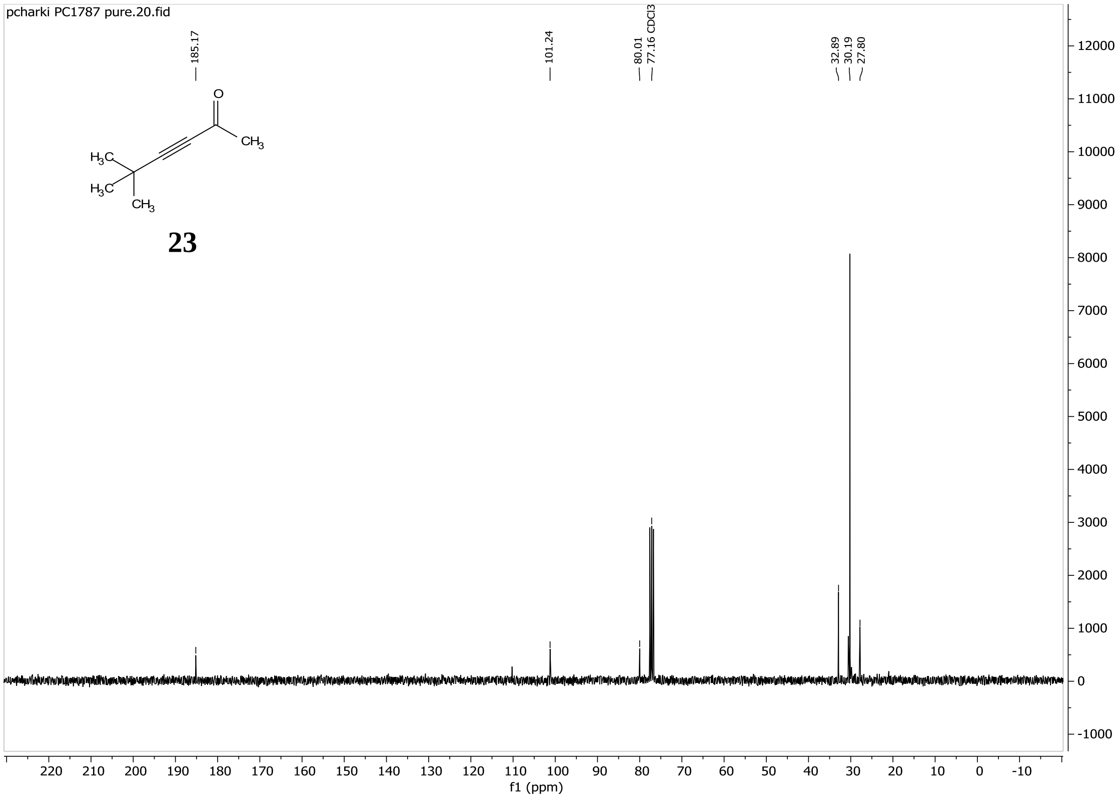

The ^{1}H NMR (300 MHz) and ^{13}C{^{1}H} NMR (76 MHz) spectrum for **24** (using $CDCl_3$ as solvent)

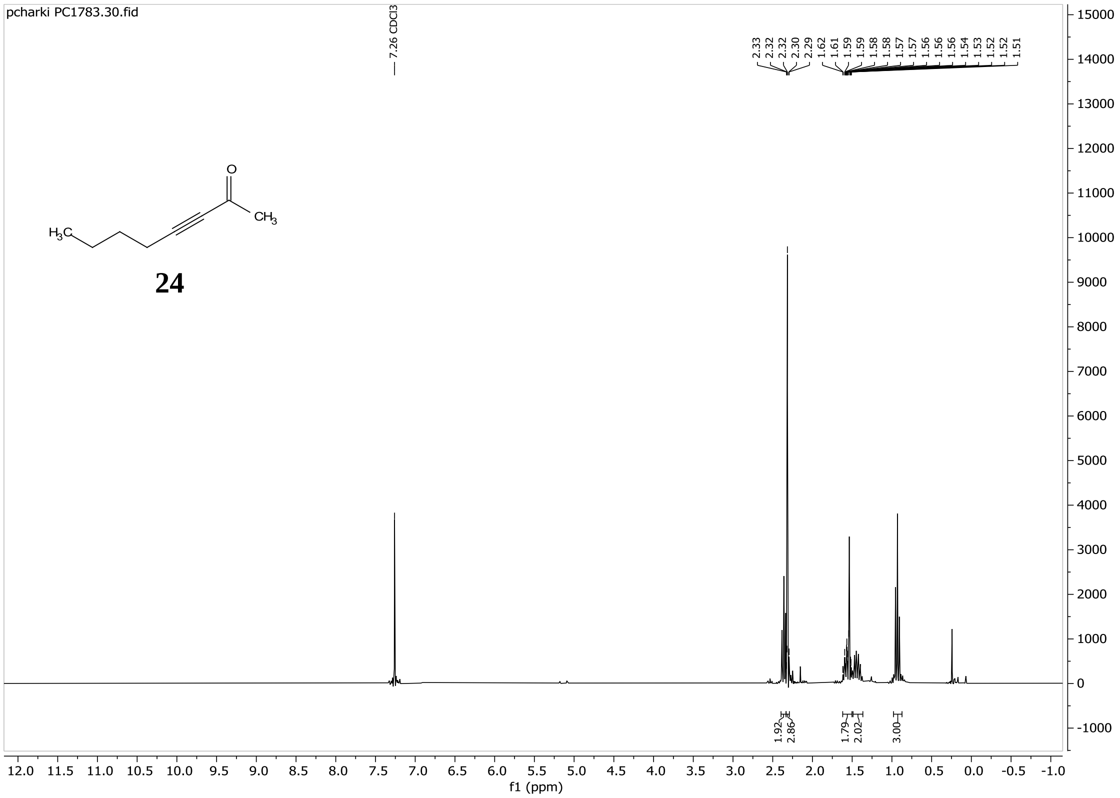

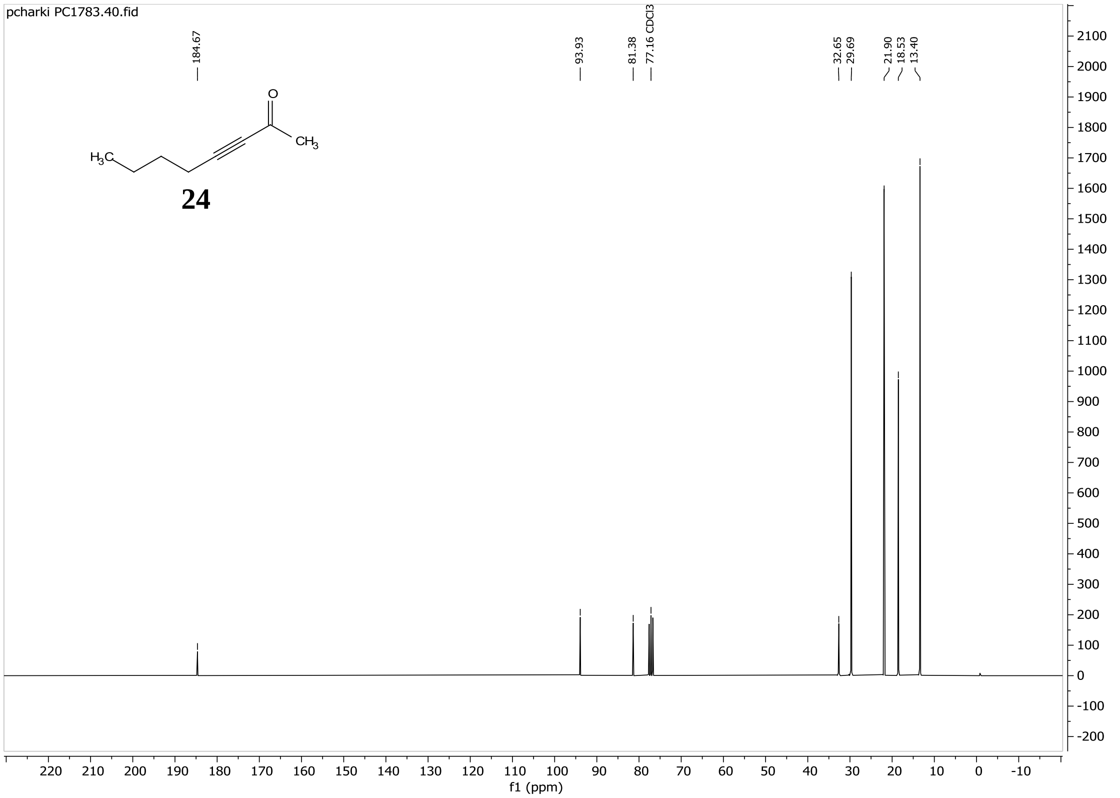

The ^{1}H NMR (300 MHz) and $^{13}C\{^{1}H\}$ NMR (76 MHz) spectrum for **25** (using $CDCl_3$ as solvent)

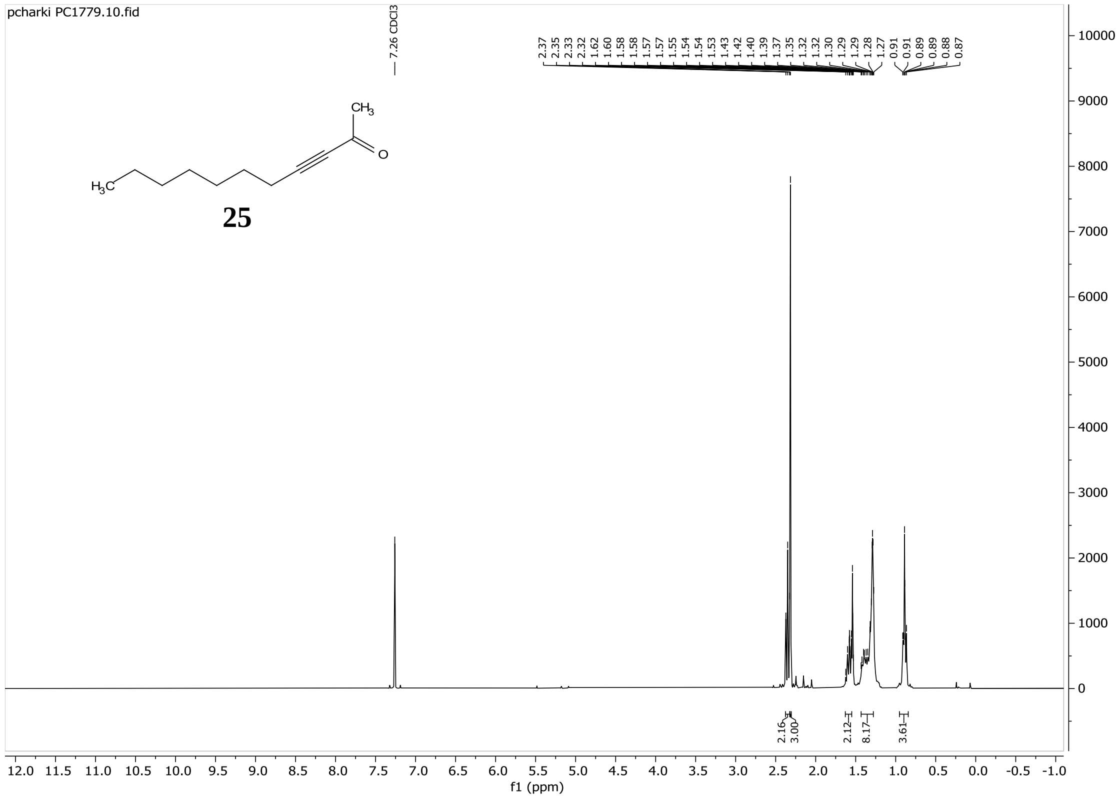

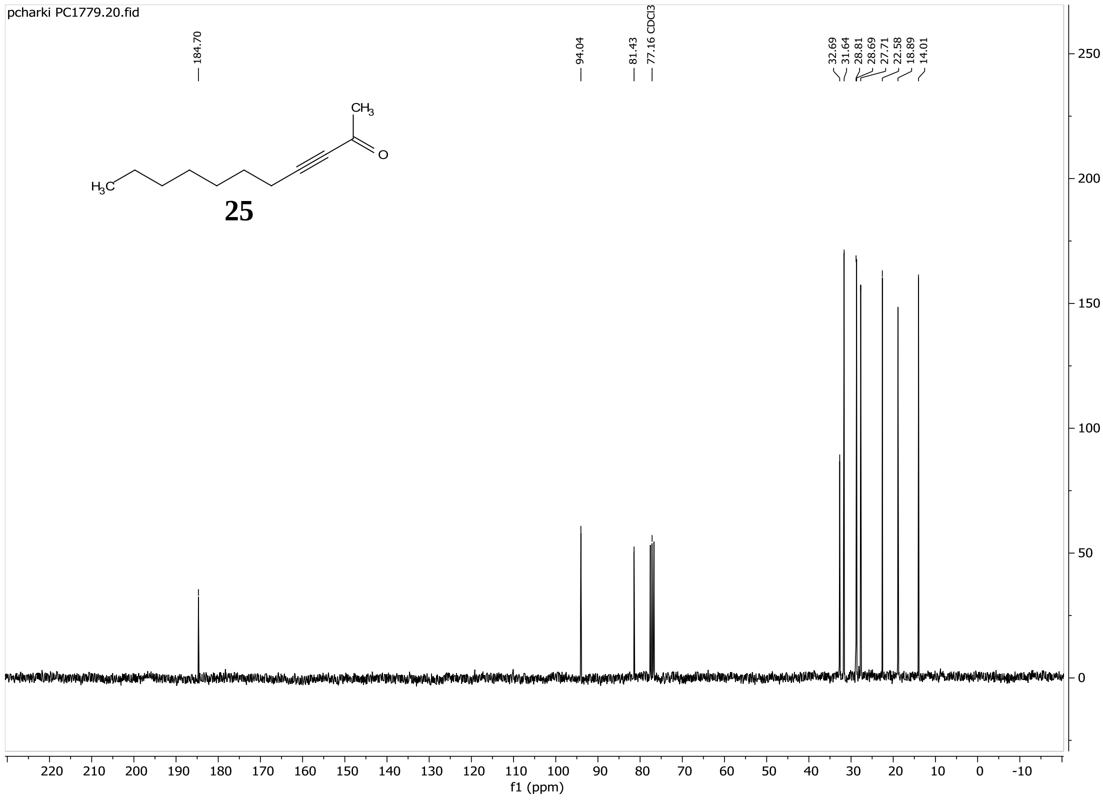

The ^{1}H NMR (300 MHz) and $^{13}C\{^{1}H\}$ NMR (76 MHz) spectrum for **26** (using $CDCl_3$ as solvent)

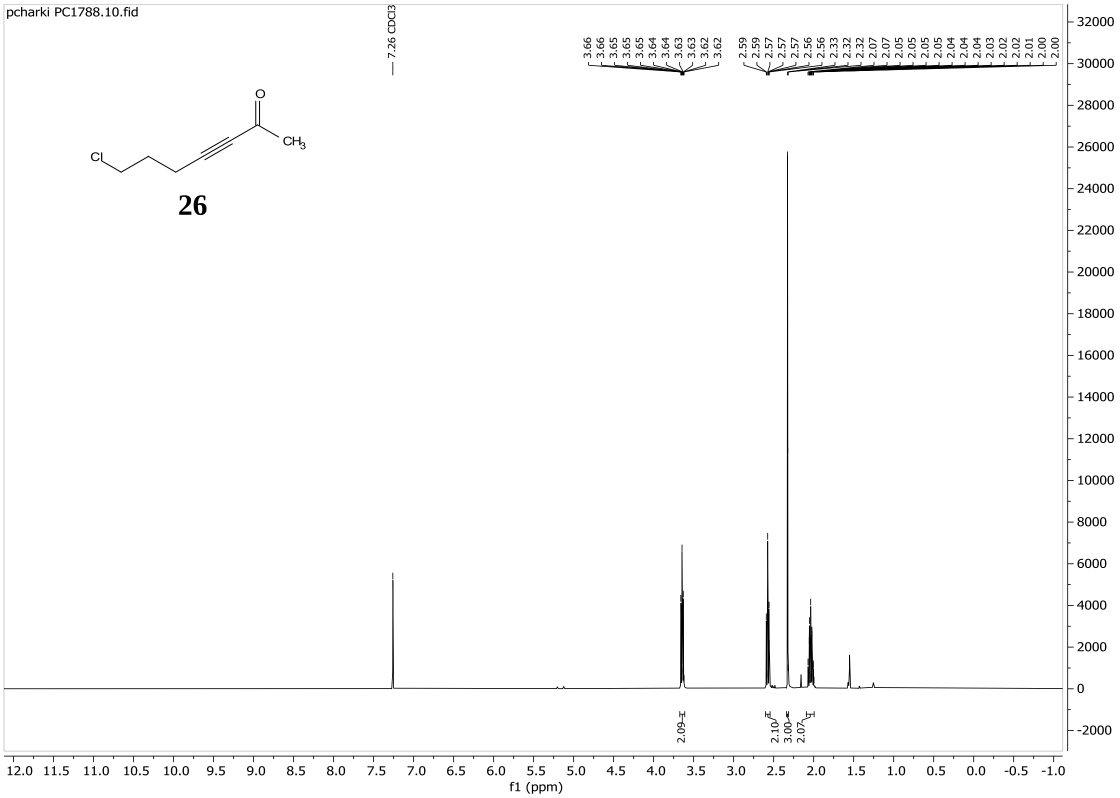

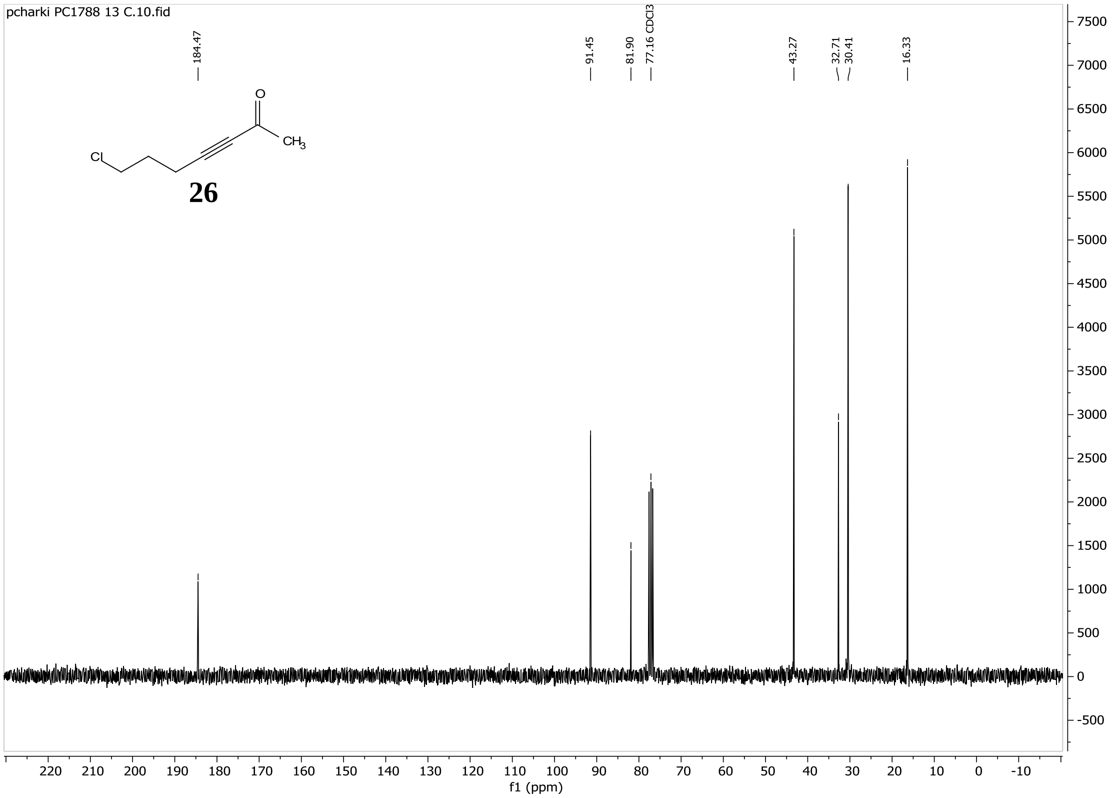

The ^{1}H NMR (300 MHz) and $^{13}C\{^{1}H\}$ NMR (76 MHz) spectrum for **27** (using $CDCl_3$ as solvent)

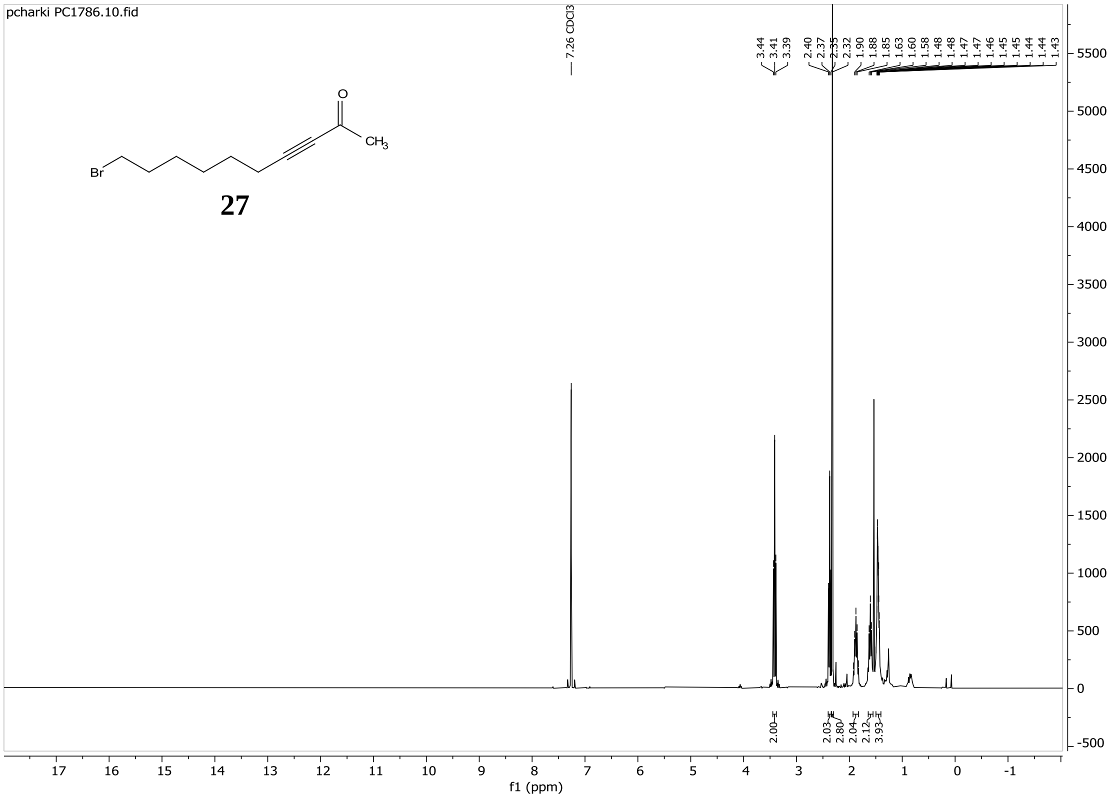

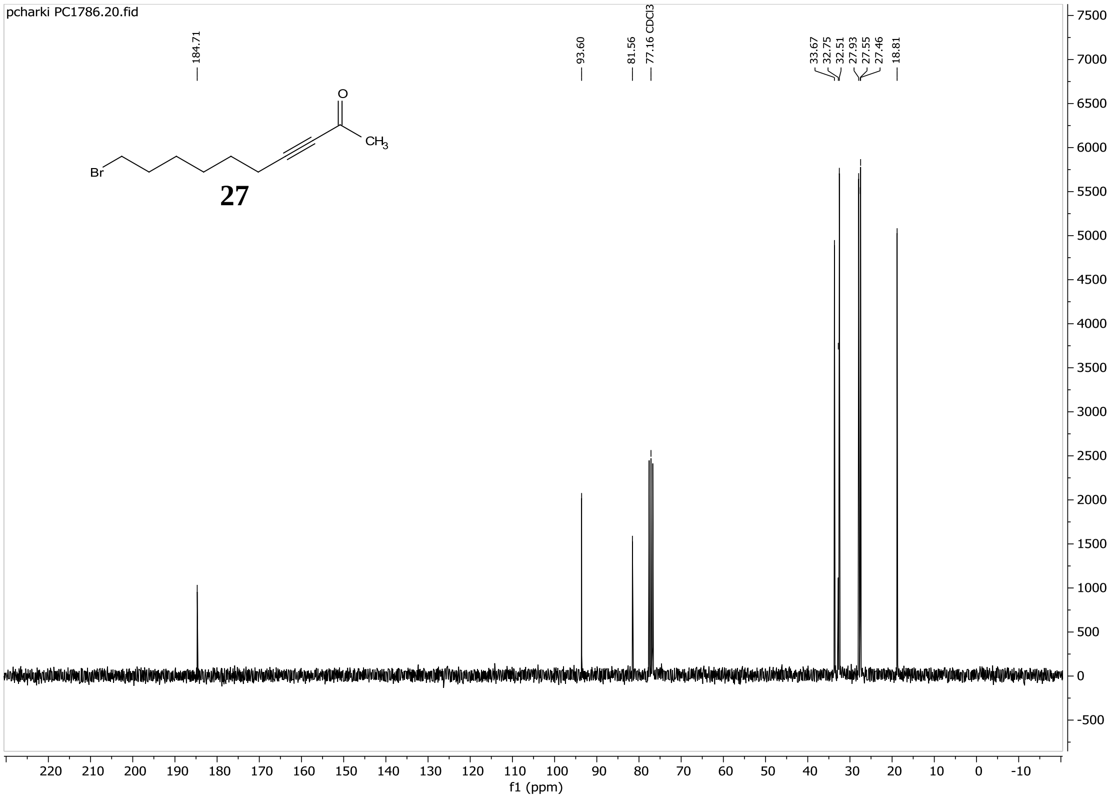

The ^{1}H NMR (300 MHz) and ^{13}C{^{1}H} NMR (76 MHz) spectrum for **28** (using $CDCl_3$ as solvent)

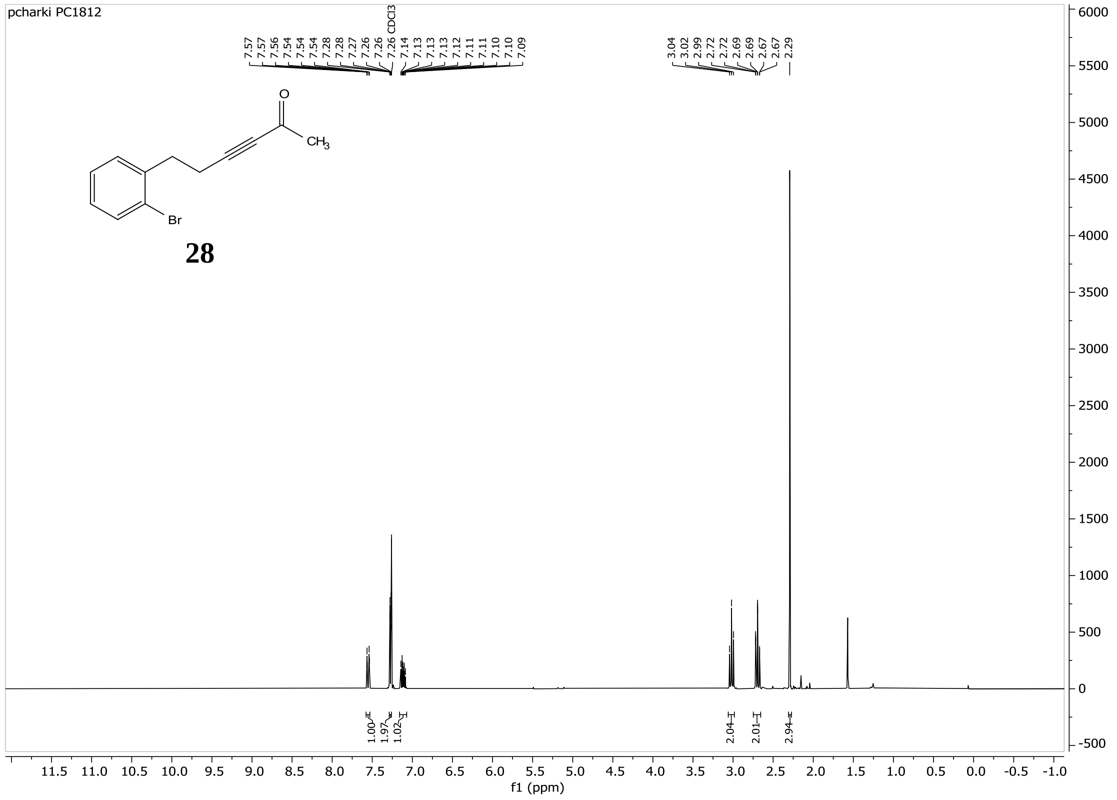

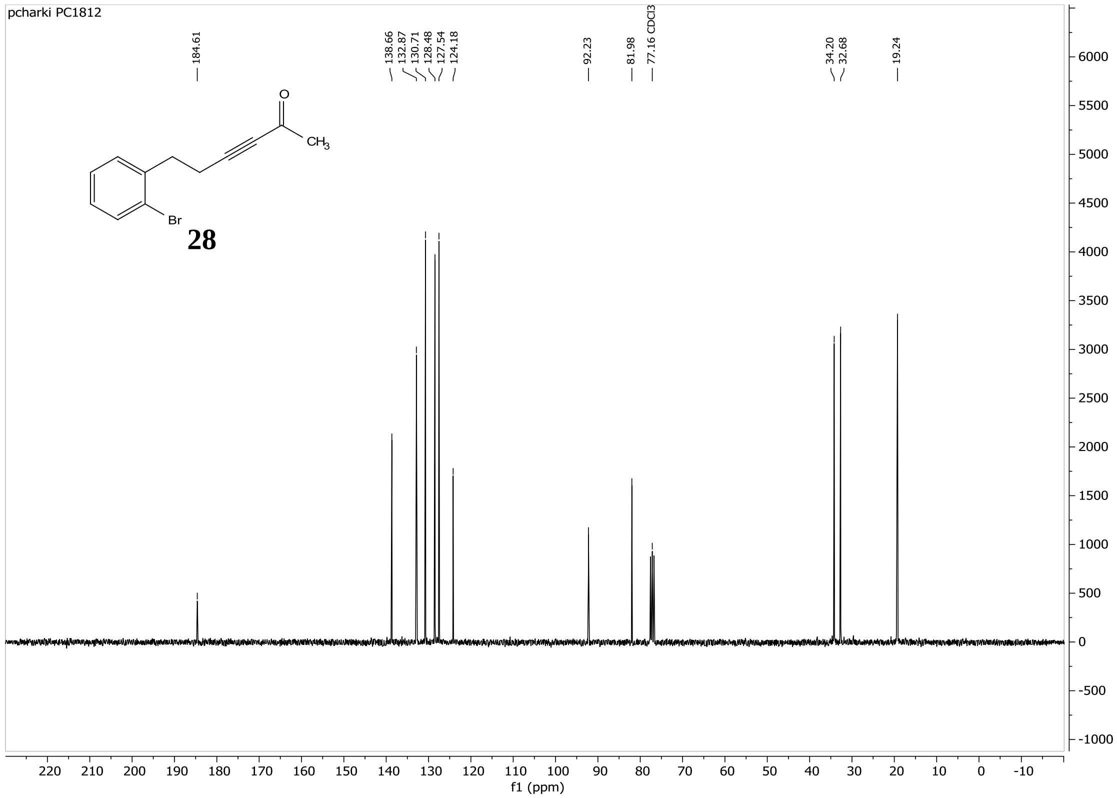

The ^{1}H NMR (300 MHz) and ^{13}C{^{1}H} NMR (76 MHz) spectrum for **29** (using $CDCl_3$ as solvent)

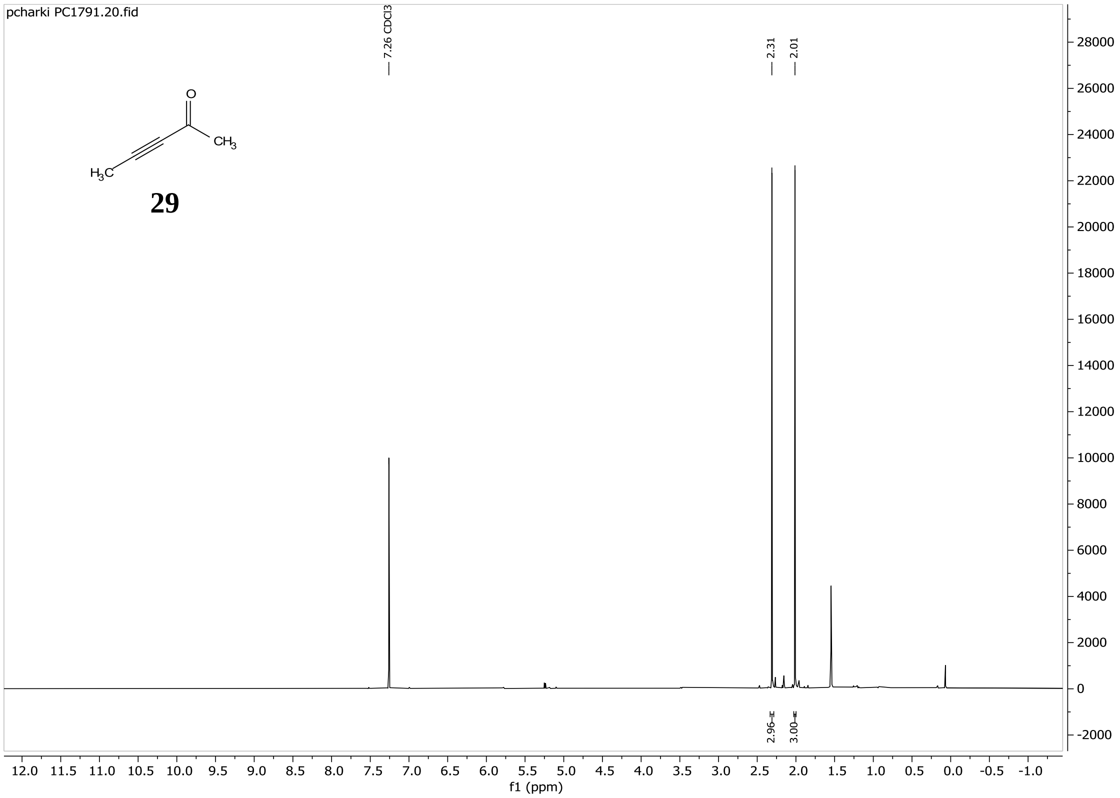

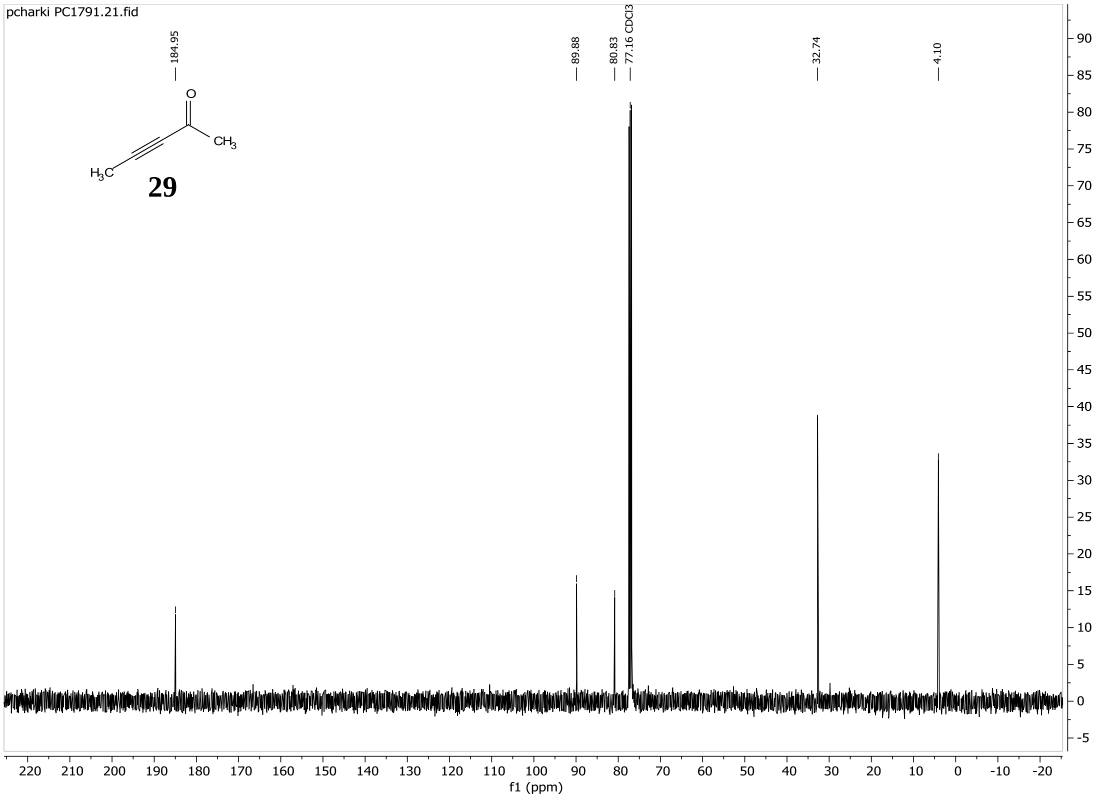

The ^{1}H NMR (300 MHz) and $^{13}C\{^{1}H\}$ NMR (76 MHz) spectrum for **30** (using $CDCl_3$ as solvent)

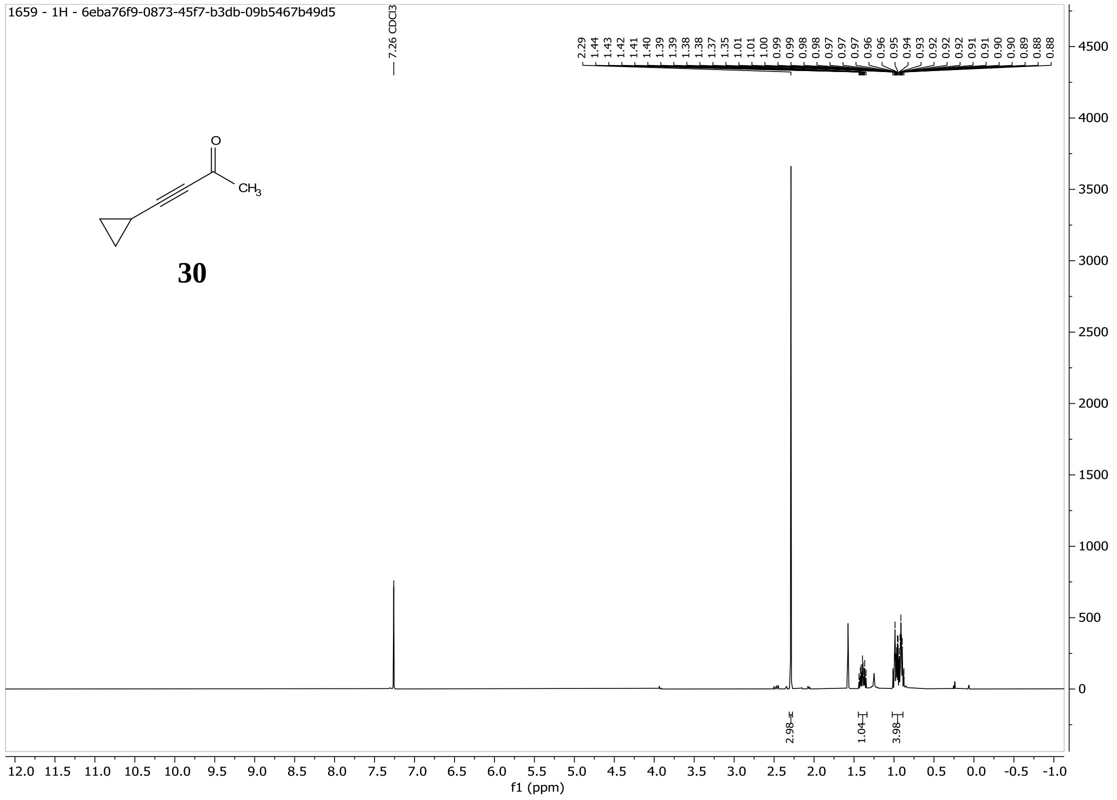

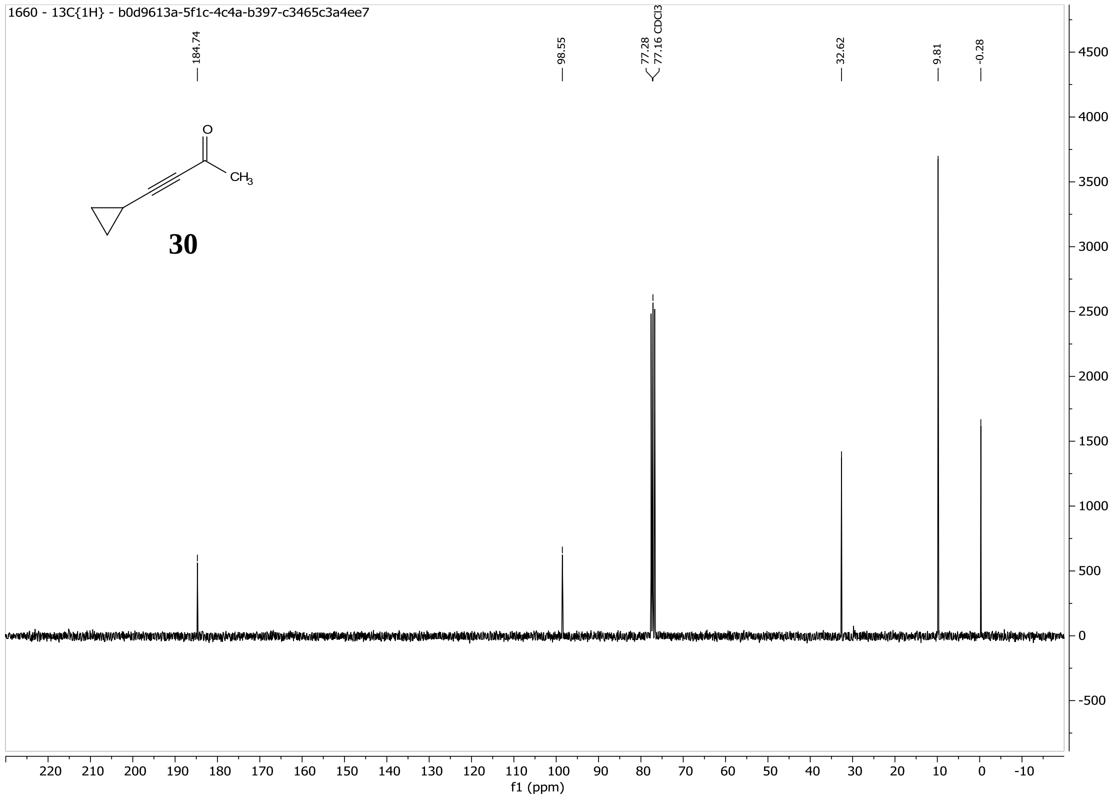

The ^{1}H NMR (300 MHz) and $^{13}C\{^{1}H\}$ NMR (76 MHz) spectrum for **31** (using $CDCl_3$ as solvent)

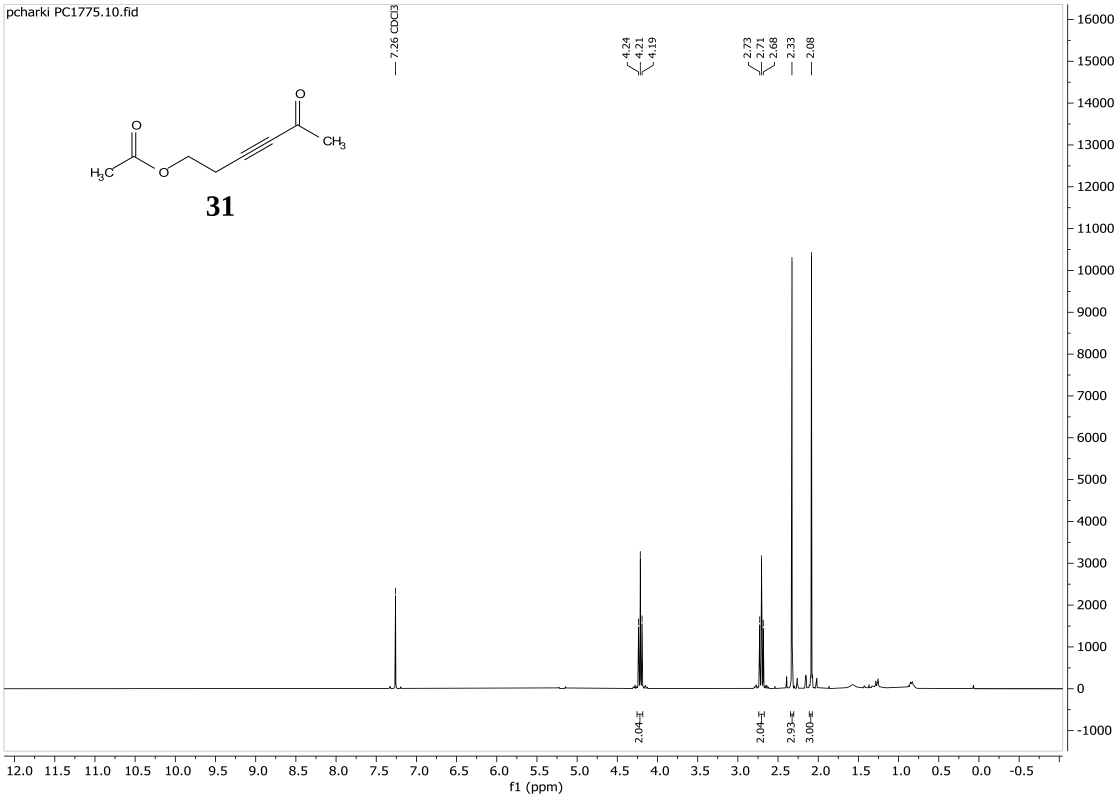

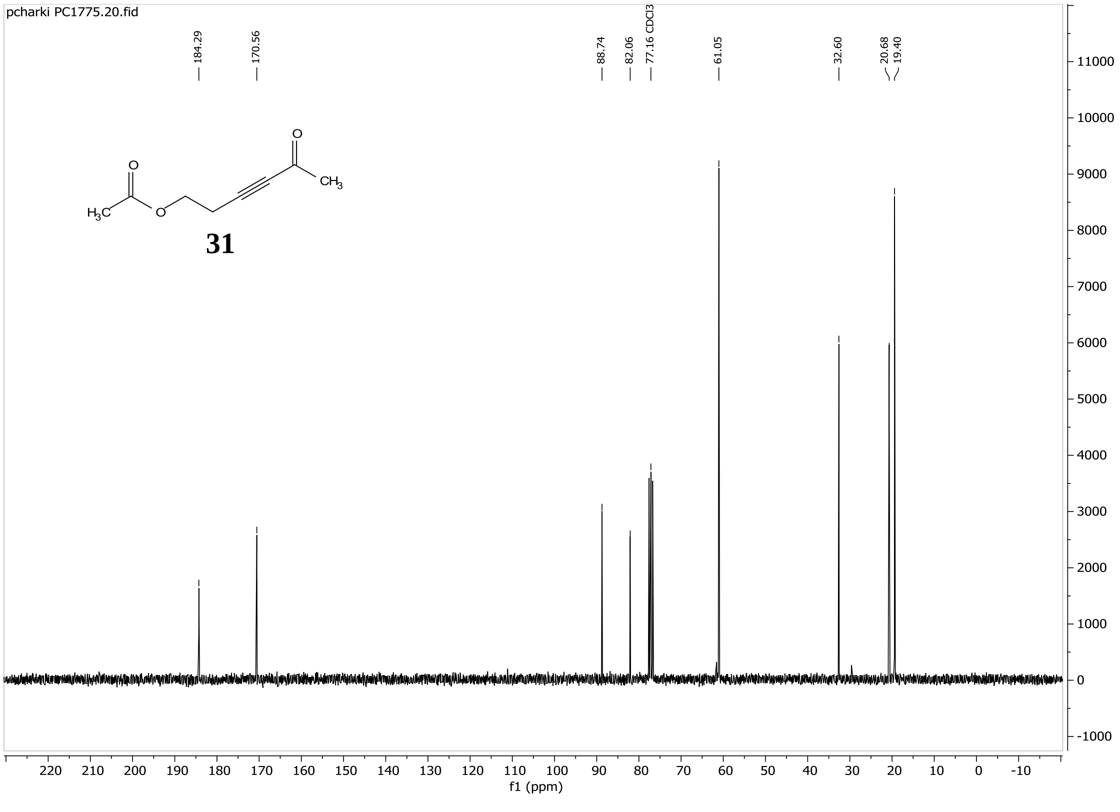

The ^{1}H NMR (300 MHz) and $^{13}C\{^{1}H\}$ NMR (76 MHz) spectrum for **36** (using $CDCl_3$ as solvent)

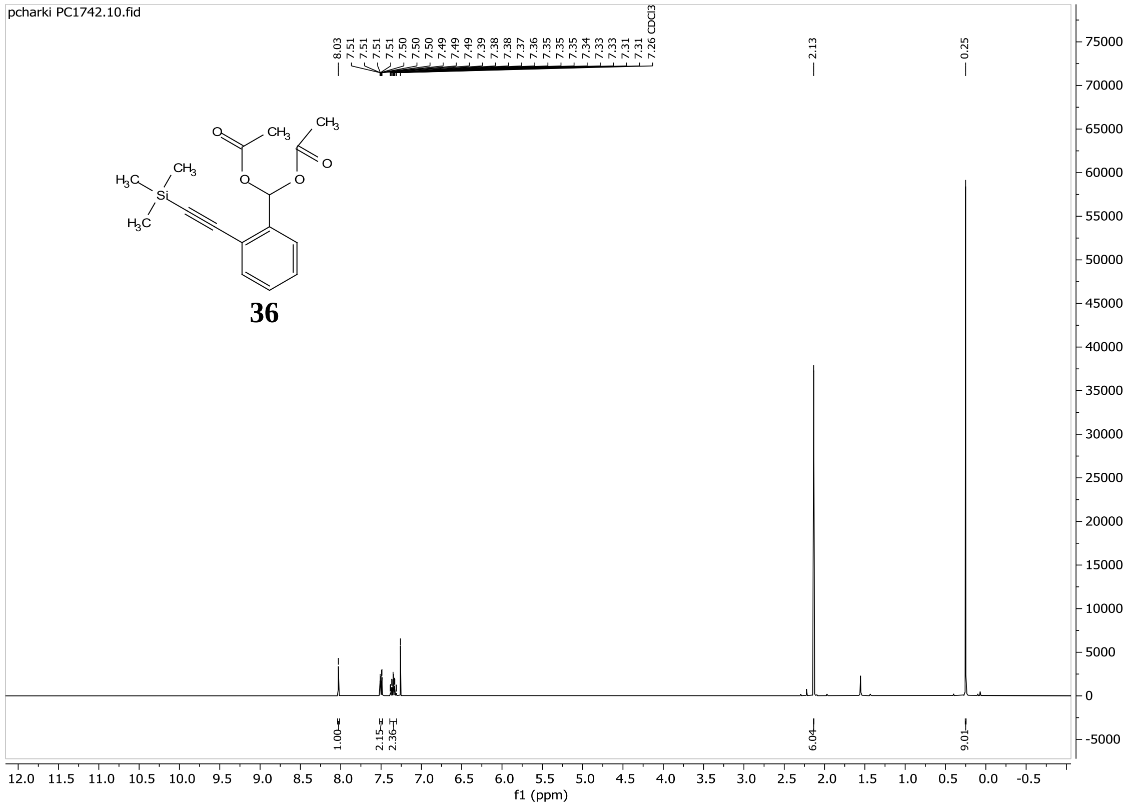

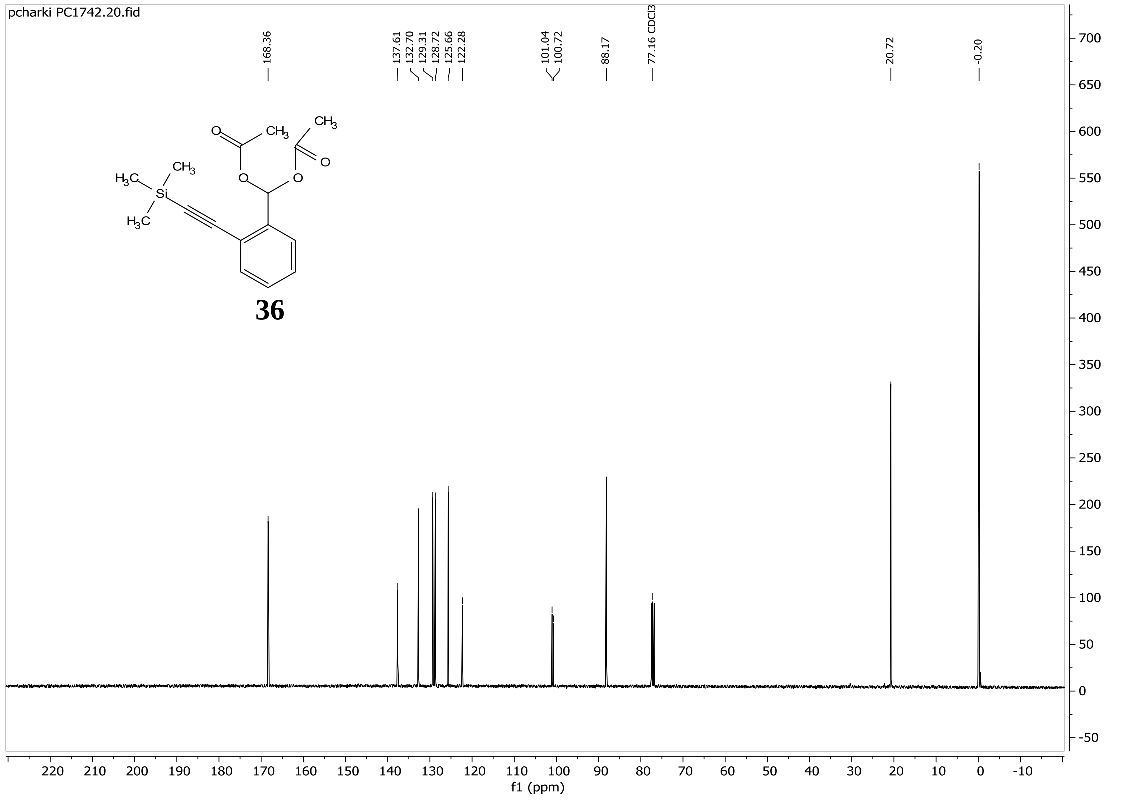

The ^{1}H NMR (300 MHz) and $^{13}C\{^{1}H\}$ NMR (76 MHz) spectrum for **42** (using $CDCl_3$ as solvent)

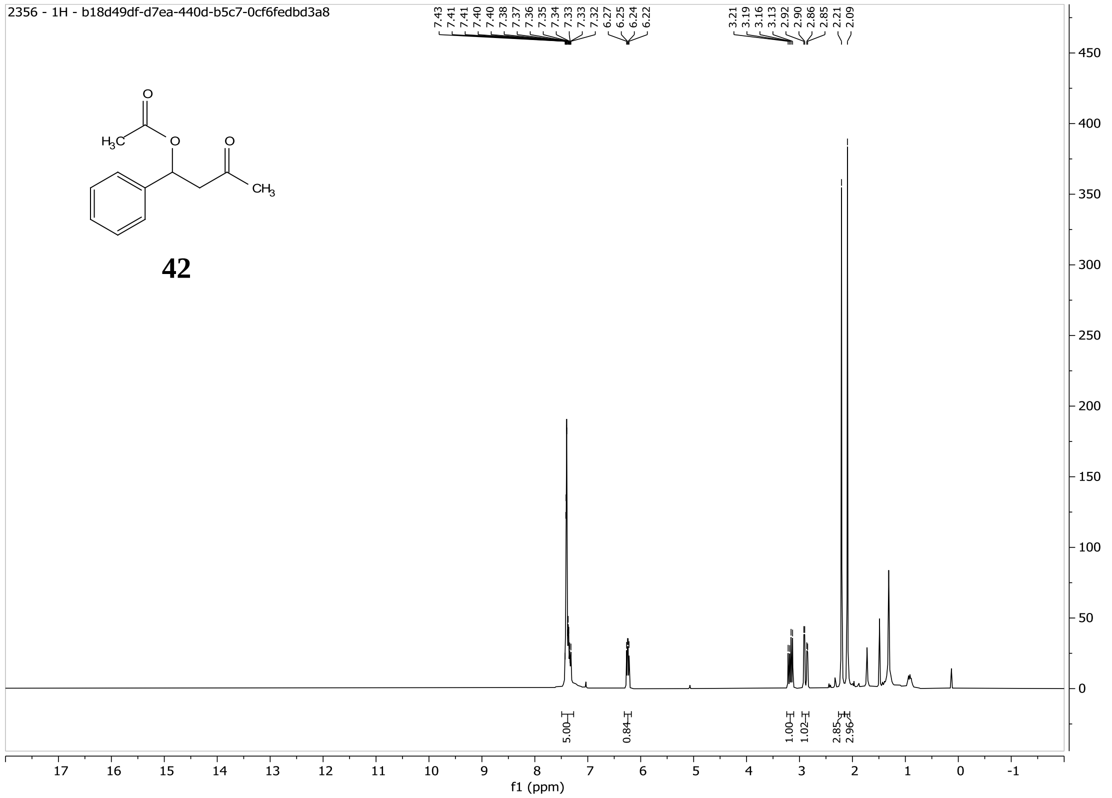

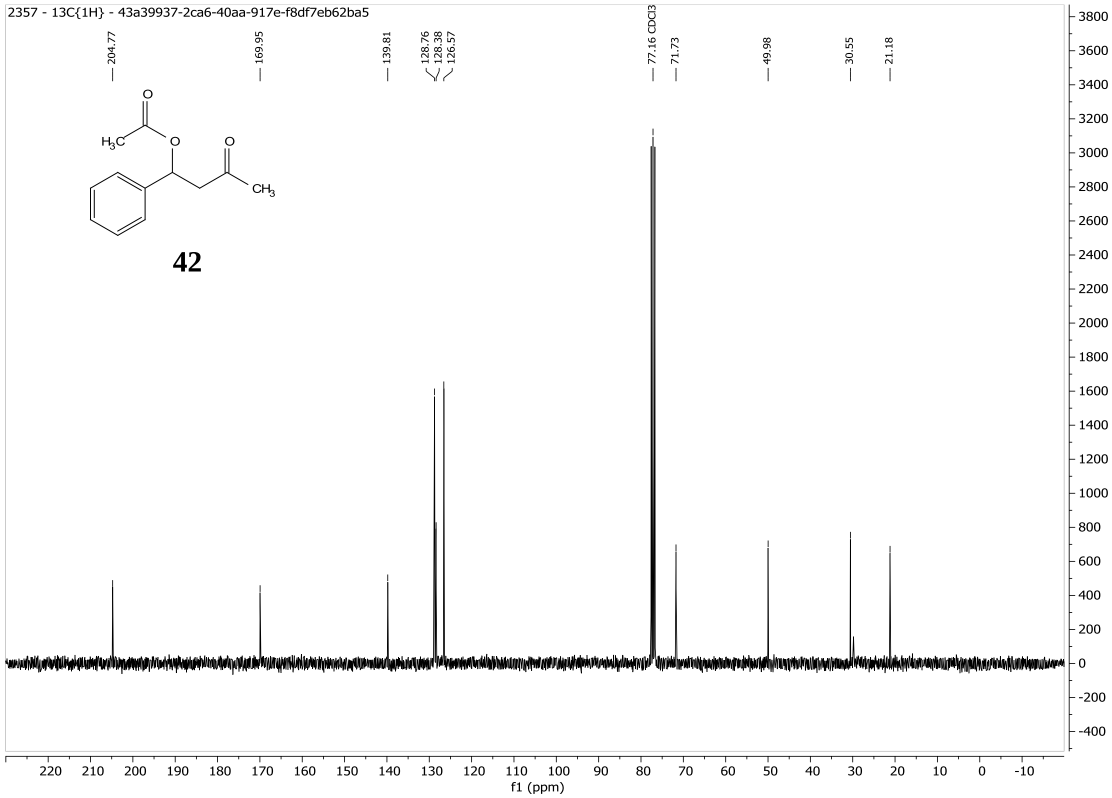

The ^{1}H NMR (300 MHz) and $^{13}C\{^{1}H\}$ NMR (76 MHz) spectrum for **43** (using $CDCl_3$ as solvent)

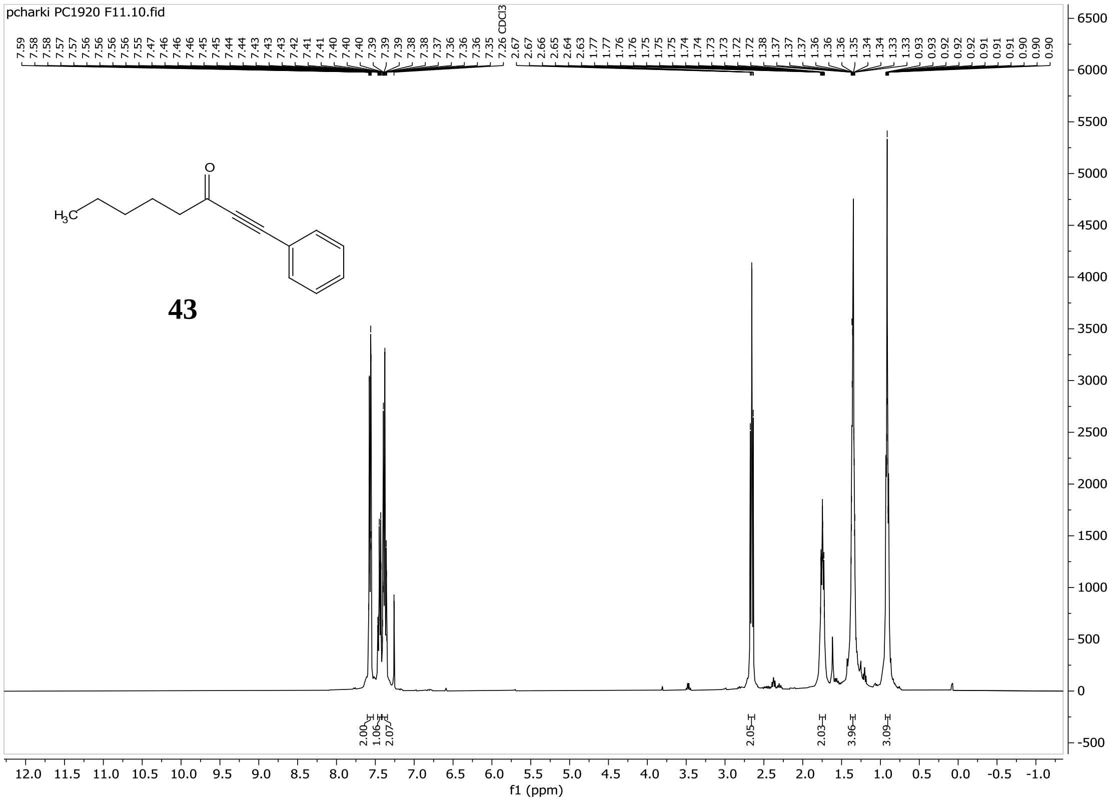

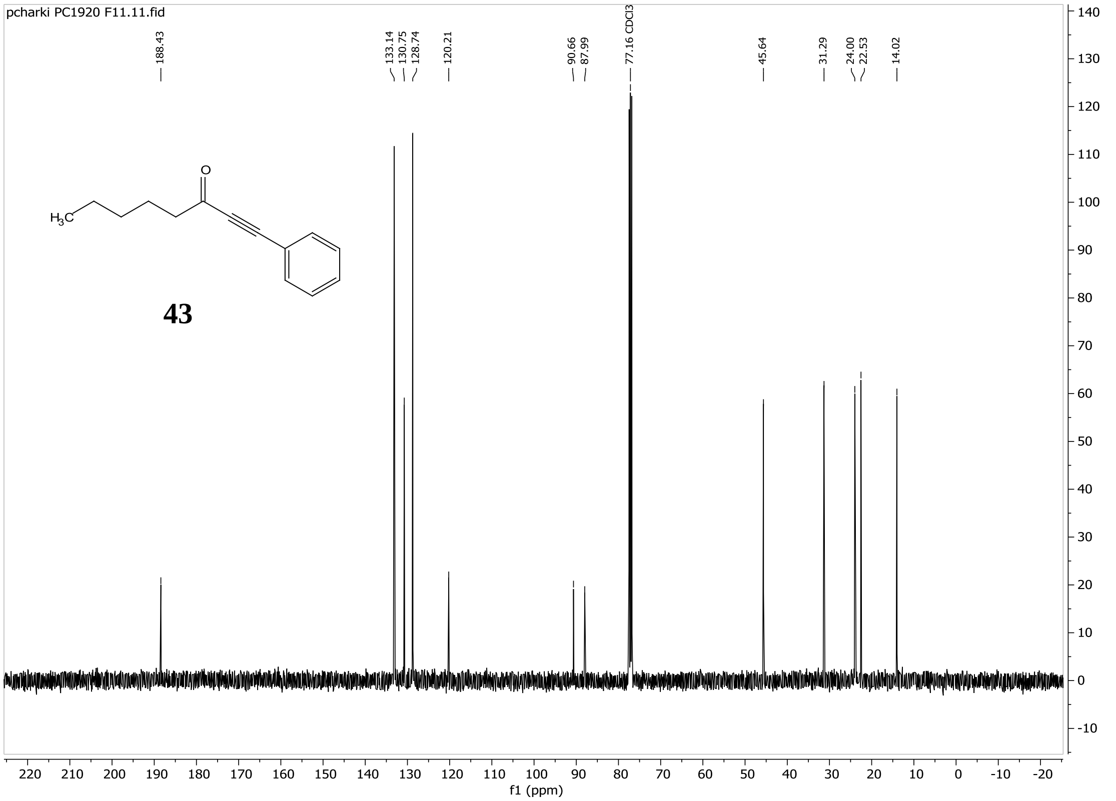

The ^{1}H NMR (300 MHz) and $^{13}C\{^{1}H\}$ NMR (76 MHz) spectrum for **44** (using $CDCl_3$ as solvent)

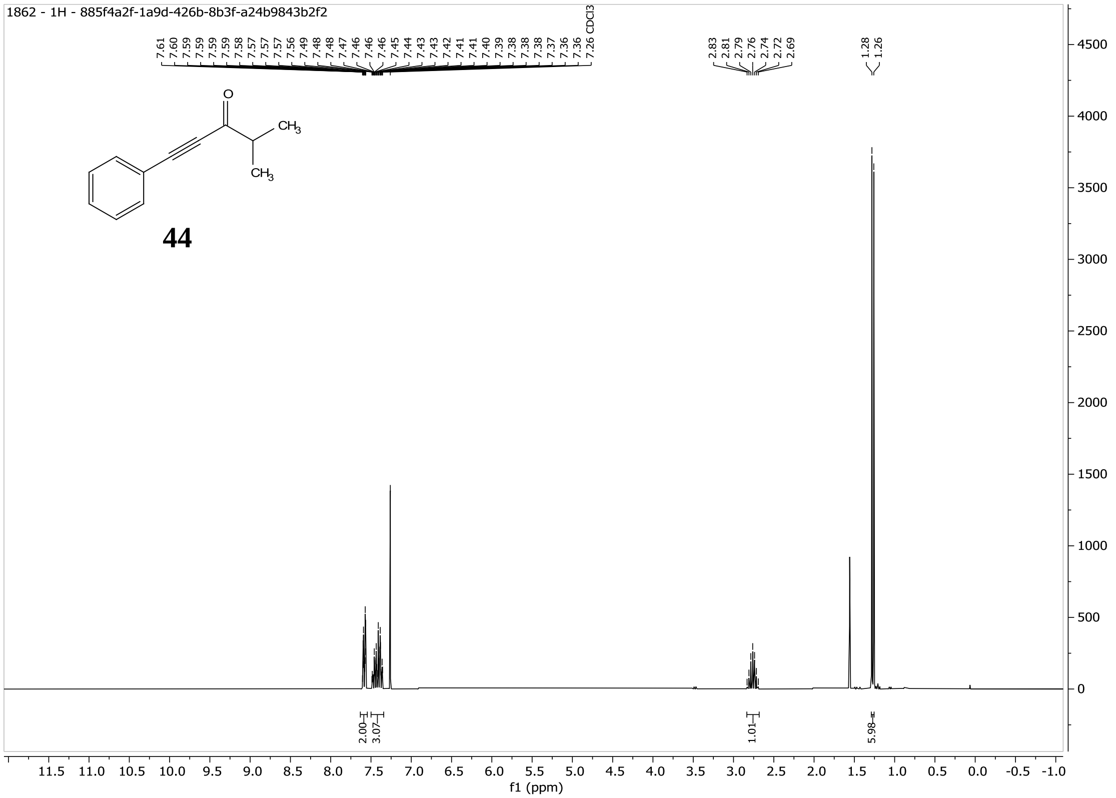

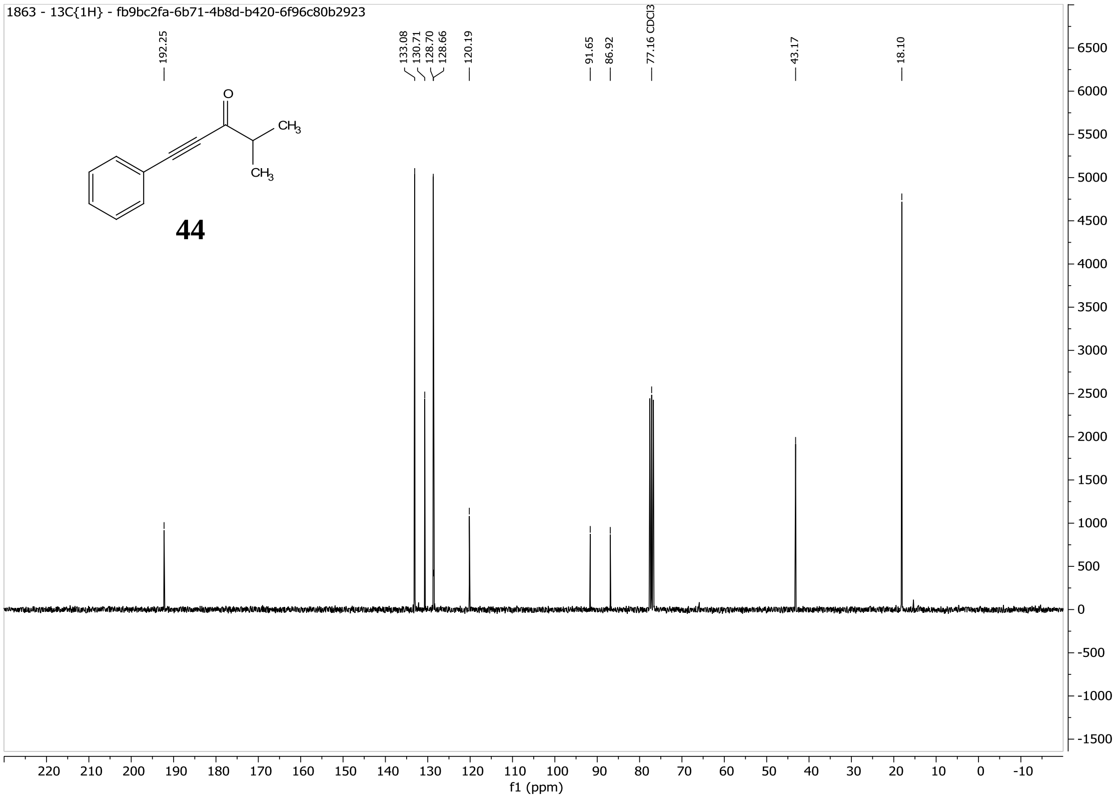

Supplement: Supplementary file 1 — Supplementary Material [file OPEN-15-e202500402-s001.pdf]
